# Synthesis of Side-Chain Liquid Crystalline Polyacrylates with Bridged Stilbene Mesogens

**DOI:** 10.3390/molecules29215220

**Published:** 2024-11-04

**Authors:** Gen-ichi Konishi, Yuki Sawatari, Riki Iwai, Takuya Tanaka, Yoshimichi Shimomura, Masatoshi Tokita

**Affiliations:** 1 Department of Chemical Science and Engineering, Institute of Science Tokyo, Tokyo 152-8552, Japan; 2 Department of Polymer Chemistry, Tokyo Institute of Technology, Tokyo 152-8552, Japan

**Keywords:** side-chain liquid crystalline polymer, nematic liquid crystal, polyacrylate, π-conjugated mesogen, birefringence, aggregation-induced emission

## Abstract

In recent years, π-conjugated liquid crystalline molecules with optoelectronic functionalities have garnered considerable attention, and integrating these molecules into side-chain liquid crystalline polymers (SCLCPs) holds potential for developing devices that are operational near room temperature. However, it is difficult to design SCLCPs with excellent processability because liquid crystalline mesogens are rigid rods, have low solubility in organic solvents, and have a high isotropization temperature. Recently, we developed near-room-temperature π-conjugated nematic liquid crystals based on “bridged stilbene”. In this work, we synthesized a polyacrylate SCLCP incorporating a bridged stilbene that exhibited a nematic phase near room temperature and could maintain liquid crystallinity for more than three months. We conducted a thorough phase structure analysis and evaluated the optical properties. The birefringence values of the resulting polymers were higher than those of the corresponding monomers because of the enhanced order parameters due to the polymer effect. In addition, the synthesized polymers inherited mesogen-derived AIE properties, with high quantum yields (*Φ*_fl_ = 0.14–0.35) in the solid state. It is noteworthy that the maximum fluorescence wavelength exhibited a redshift of greater than 27 nm as a consequence of film formation. Thus, several unique characteristics of the SCLCPs are unattainable with small molecular systems.

## 1. Introduction

Liquid crystal polymers are widely used in industrial applications due to their excellent processability and favorable material properties [1,2,3,4,5,6,7,8,9,10,11,12,13,14]. Of particular interest are side-chain liquid crystal polymers (SCLCPs) [15,16,17,18,19,20,21,22,23,24,25], in which mesogenic units are attached to the side chains of linear polymers such as polyacrylate and polymethacrylate. SCLCPs exhibit liquid crystalline phases over a broader temperature range than small molecule liquid crystals and mimic the behavior of small molecule liquid crystals above the glass transition temperature (*T*_g_). This liquid crystalline nature allows for the molecular orientation to be controlled by external fields such as mechanical stress, electric fields, and magnetic fields [1,2,3,4,5,6,7,8,9,10,11,12,13,14]. In addition, the molecular orientation of LC polymers can be controlled using anisotropic surfaces (as in this study) and photoalignment (e.g., [16]). Additionally, SCLCPs can form nanostructures through higher order smectic and columnar phases [26,27,28,29]. Below *T*_g_, the liquid crystal phase that exists above *T*_g_ can be vitrified, retaining its anisotropic properties. Among liquid crystal phases, the nematic (N) phase is the most fluid, is highly responsive to external fields, and is suitable for large-area applications [30]. These properties, unique to the N phase, distinguish it from other liquid crystal phases and extend its range of applications beyond traditional uses, including displays [31,32] and polarizing films, into advanced optical materials. The properties exhibited by SCLCPs are highly attractive for the development of materials that leverage the unique optical, luminescent, and electronic properties of π-conjugated systems [33,34,35,36,37,38,39,40,41,42,43,44,45,46]. In particular, SCLCPs with liquid crystalline organic semiconductors in the side chains can be one of the most powerful tools for fabricating film-like devices with bulk arrays of organic semiconductors. In other words, it is effective for the realization of advanced materials based on functional liquid crystalline π-conjugated molecules, such as displays and molecular electronics. However, π-conjugated molecules with functional groups typically have high melting points, and SCLCPs incorporating these as mesogens often struggle to exhibit the nematic (N) phase or only exhibit it at elevated temperatures above 100 °C [45]. In addition, the introduction of a long-chain alkyl group into the mesogen lowers the isotropization temperature but tends to produce a smectic phase with high crystallinity and low operability [47]. As a result, it is challenging to achieve room temperature N-phase behavior in SCLCPs that feature π-conjugated mesogens with photo/electronic functionality.

Recently, we developed a novel aggregation-induced emission luminogen (AIEgen) [48,49,50,51,52,53,54], “seven-membered bridged stilbene”, based on 4-phenyl stilbene (**PST**) with a seven-membered ring structure, specifically 3,8-diphenyl-6,7-dihydro-5*H*-benzo[7]annulene (**DPB[7]**) [55,56,57]. We reported that **DPB[7]**, when modified with appropriate alkyl tails at both ends, exhibits the N phase around room temperature [58]. This bridged stilbene liquid crystal (LC) possesses the unique ability to maintain the N phase over a wide temperature range while exhibiting luminescent properties. Such π-conjugated liquid crystals with low isotropization temperatures have the potential to overcome various limitations of conventional SCLCPs. Using this LC, we have designed a π-conjugated SCLCP that exhibits the N phase at room temperature.

In this study, we synthesized polyacrylate and polymethacrylate SCLCP with the **DPB[7]** mesogen, which exhibits a nematic phase at room temperature. We analyzed the detailed phase structure and evaluated the birefringence and photophysical properties. Our findings revealed several unique properties of SCLCPs that are not achievable with small molecule systems.

## 2. Results and Discussion

### 2.1. Synthesis of Monomers and Polymers

**Scheme 1:**
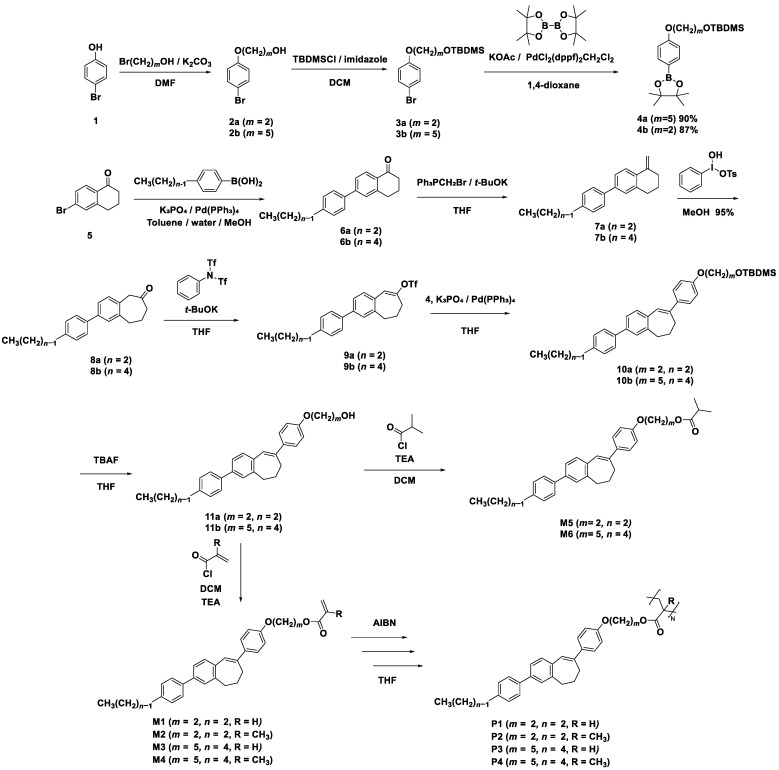
Synthesis of acrylic monomers (**M1** and **M3**), methacrylic monomers (**M2** and **M4)**, and model compounds of monomer (**M5** and **M6**) with **DPB[7]** skeleton and flexible chains (*m* + 1, *n*), and corresponding polymers (**P1**–**P4**).

We synthesized side-chain liquid crystalline polyacrylates and polymethacrylates incorporating the 3,8-diphenyl-6,7-dihydro-5*H*-benzo[7]annulene (**DPB[7]**) skeleton with alkyl terminals (carbon number: *n* = 2, 4), alkoxy spacers (carbon number: *m* = 2, 5), and various terminal ester groups. The chemical structures and synthetic procedures of the monomers (**M1**–**M4**), polymers (**P1**–**P4**), and model compounds (**M5** and **M6**) are illustrated in Scheme 1. The synthesis of the monomers can be outlined as follows: Suzuki–Miyaura cross-coupling reaction [59,60] between 6-bromo-1-tetralone (**5**) and 4-alkylphenylboronic acid (with alkyl group carbon numbers: *m* = 2, 4) provided **6**, followed by a Wittig reaction to yield **7**; a ring expansion reaction using hypervalent iodine reagent [hydroxy(tosyloxy)iodo]benzene (HTIB) then produced **8**; triflation of the carbonyl group yielded **9**, and subsequent Suzuki–Miyaura cross-coupling of **9** with 4-alkoxyphenylboronic acid pinacol esters (alkoxy group carbon numbers: *n* = 2, 5) provided **10**; deprotection of the *tert*-butyldimethylsilyl (TBDMS) group of **10** using tetrabutylammonium fluoride (TBAF) resulted in **11**. Finally, the introduction of various acyl chlorides afforded the acrylic monomers (**M1** and **M3**), methacrylic monomers (**M2** and **M4**), and isobutyrate derivatives (**M5** and **M6**). The monomers were purified via column chromatography on silica gel and recrystallization. The chemical structures of the monomers and model compounds were confirmed through ^1^H-NMR, ^13^C-NMR, FT-IR, and high-resolution mass spectrometry (HRMS). These spectral data and the synthesis of **1**–**4** are provided in Supporting Information (SI).

Radical polymerization of the acrylic monomers (**M1** and **M3**) and methacrylic monomers (**M2** and **M4**) was conducted using 6.5 wt% azobisisobutyronitrile (AIBN) as the initiator, resulting in the corresponding polymers (**P1**–**P4**). The crude polymers were purified by reprecipitation in methanol. The polymer structures were confirmed by ^1^H-NMR and ^13^C-NMR spectroscopy. The number-average molecular weight (*M*_n_) and weight-average molecular weight (*M*_w_) of the polymers were determined via gel permeation chromatography using polystyrene standards. Furthermore, thermogravimetric analysis (TGA) measurements were performed on **P1**–**P4** at a rate of 20 °C min^−1^ (Figures S1–S4), determining degradation temperatures at a 10% weight loss (*T*_10_). The results are summarized in Table 1. The polymethacrylates exhibited higher molecular weights compared with the polyacrylates.

### 2.2. Phase Transition Behaviors

**Figure 1:**
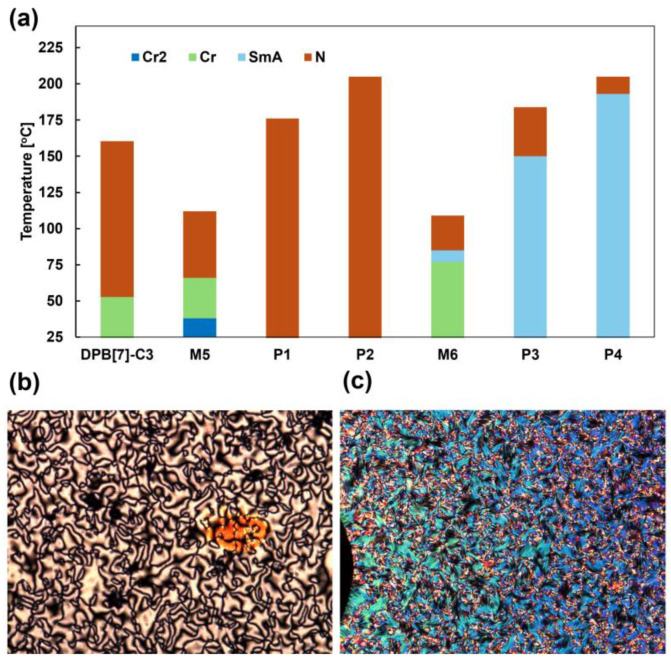
Summary of phase transition behavior. (**a**) Phase transition temperature and range diagram for **DPB[7]** monomers (**DPB[7]-C3**, **M5**, and **M6**) and **DPB[7]** polymers (**P1**–**P4**) observed by DSC measurement in second heating (for **M5** and **M6**) and third heating (for **DPB**[7]**-C3** and **P1**–**P4**) process. POM images of (**b**) schlieren texture for **P2** at 193 °C and (**c**) fan-shape texture for **P4** at 128 °C.

Liquid crystalline phases of the polymers, monomers, and model compounds were identified using polarized optical microscopy (POM). Phase transition temperatures and enthalpies were determined through differential scanning calorimetry (DSC). POM observations suggested that **M1** and **M2** exhibited only the nematic (N) phase, while **M3** and **M4** exhibited both the N phase and the smectic A (SmA) phase. It is suspected that the vinyl groups in **M1**–**M4** underwent either conversion to another compound or oligomerization owing to thermal reactions. Therefore, we synthesized model compounds (**M5** and **M6**) in which the (meth)acrylate group was replaced with an isobutyrate group. **M5** and **M6** were used for comparison with the monomers and polymers in subsequent analyses. The phase transition temperatures and enthalpies (Δ*H*) of **M5** and **M6** and **P1**–**P4** are summarized in Table 2, and the typical POM images of **P2** and **P4** are illustrated in Figure 1. The DSC curves for **M5** (second scan), **M6** (second scan), and **P1**–**P4** (third scan) are shown in Figures S5–S10, while the POM images for **M5**, **M6**, **P1**, **P3**, and the rest of **P2** and **P4** are shown in Figures S11–S16.

**M5** exhibited the N phase between 66.4 °C and 111.6 °C during the heating process, while **M6** exhibited the SmA phase between 77.4 °C and 84.8 °C and the N phase between 84.8 °C and 109.3 °C. POM images of the schlieren textures in the N phase for **M5** and **M6** are illustrated in Figures S11 and S12, and fan-shaped textures in the SmA phase of **M6** are also presented in Figure S12. The flexible chain length, excluding the terminal ester group, (in terms of carbon and oxygen atoms) is three or fewer for **M5** and four or more for **M6**. This trend is similar to our **DPB**[7] liquid crystals [58], where the SmA phase emerges when the flexible chain length exceeds three atoms.

Next, we describe the phase transition behaviors of **P1**–**P4**. **P1** and **P2** exhibited N phases over the ranges of 25.0–176.3 °C and 25.0–205.0 °C, respectively, during the heating process. In these polymers, the N phase was successfully maintained at room temperature (25 °C), and schlieren textures were observed, as illustrated in Figure 1b. **P3** displayed a smectic A (SmA) phase between 25.0 °C and 150.3 °C and an N phase from 150.3 °C to 184.2 °C, while **P4** showed a SmA phase from 25.0 °C to 193.8 °C and an N phase from 193.8 °C to 205.0 °C. In the SmA phases of **P3** and **P4**, fan-shaped textures were observed (Figure 1c). The glass transition temperature (*T*_g_) was observed at 60.6 °C for P1, and 25.6 °C for **P3**, while no *T*_g_ was detected for **P2** and **P4**.

The clearing points of **P1** and **P2** were 64.7 °C and 93.4 °C higher than those of **M5**, respectively. Similarly, the clearing points of **P3** and **P4** were higher than those of **M6** by 74.9 °C and 95.7 °C, respectively, and the N-SmA phase transition temperatures were higher by 65.5 °C and 109.0 °C. These results, indicating higher phase transition temperatures due to the polymer effect, are consistent with previously reported findings for SCLCPs. Notably, these liquid crystalline phases were maintained even after more than three months, as illustrated in Figures S13 and S14. The room temperature N phase observed in **P1** and **P2** offers significant advantages for optoelectronic applications operating at room temperature. Finally, upon 25.0 °C, the LC temperature ranges (Δ*T*_LC_) for **P1**–**P4** were 151.3 °C, 180.0 °C, 159.2 °C, and 180.0 °C, respectively. These values were considerably larger than those of the model monomers **M5** and **M6** (Δ*T*_LC_ = 45.2 °C and 31.9 °C, respectively).

### 2.3. Structure Analysis of LC Phase

**Figure 2:**
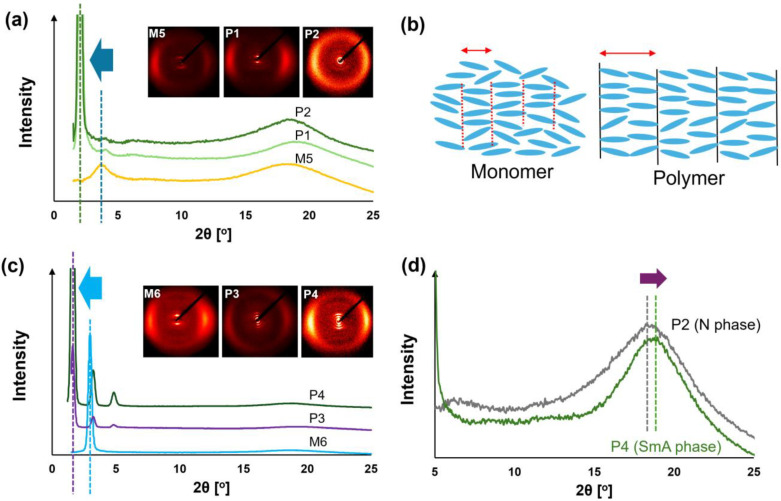
WAXD intensity profiles and expected structure in LC phase. (**a**) WAXD intensity profile in nematic (N) phase for **M5** (80 °C), **P1** (40 °C), and **P2** (30 °C). The 2*θ*_small_ values are shifted to small angle region due to the polymer effect. (**b**) Schematic illustration of molecular packing in N phase with cybotactic cluster for monomer (monolayer) and polymer (double-layer). (**c**) WAXD intensity profile in smectic A phase for **M6** (80 °C), **P3** (30 °C), and **P4** (30 °C) The 2*θ*_small_ values are shifted to small angle region due to the polymer effect. (**d**) WAXD intensity profile for **P2** and **P4** in wide-angle region recorded at 30 °C. The 2*θ*_wide_ value for SmA phase is slightly larger than that for N phase.

To investigate changes in the molecular arrangement of the LCs due to polymer effect, we performed wide-angle X-ray diffraction (WAXD) measurements for **P1**–**P4** and the corresponding monomers **M5** and **M6** (Figures S17–S34). The WAXD profiles are summarized in Figure 2, in which the magnetic field is in the vertical direction of the paper. In the N phase, the peak observed in the small-angle region of the WAXD profile (2*θ*_small_) corresponded to the layer spacing (*d*_LC_) along the molecular long axis. Also, in the SmA phase, not only the first-order peak (2*θ*_small_) but also second- or third-order peaks were observed for P3 and P4, while the peak in the wide-angle region (2*θ*_wide_) corresponded to the average distance between mesogens (*d*_mesogen_) along the molecular short axis. The values of the small-angle and wide-angle peaks (2*θ*_small_, 2*θ*_wide_), along with the corresponding *d*-spacing values (calculated using Bragg’s law) and the molecular length/side-chain length (*l*) (obtained from DFT calculations (Figures S35 and S36, and Tables S1 and S2)) are presented in Table 3.

In the N phase of **M5** (at 80 °C), **P1** (at 40 °C), and **P2** (at 30 °C), broad scattering peaks in the wide-angle region and sharp peaks in the small-angle region were observed (Figure 2a). In the N phase, there was no long-range positional order, and typically, only broad scattering in the wide-angle region was detected in WAXD measurements. Therefore, the observed small-angle peaks suggest the formation of smectic-like micro aggregates, specifically cybotactic clusters. The azimuthal profiles of diffraction in the small-angle region for **M5**, **P1**, and **P2** (Figures S19, S25 and S28) demonstrated that the scattering in the small-angle region was orthogonal to that in the wide-angle region. These results indicate that the cybotactic clusters [61,62,63] are SmA-type, with no uniform tilt in the domain. Notably, the ratio of the small-angle peak intensity to the wide-angle peak intensity was more than 10 times greater in **P1** and **P2** than in **M5**, suggesting that cybotactic clusters form more readily in **P1** and **P2** than in **M5**.

The 2*θ*_small_ values for **M5**, **P1**, and **P2** were 3.60°, 2.09°, and 2.01°, respectively, corresponding to *d*_LC_ values of 24.6 Å, 42.4 Å, and 44.0 Å, respectively. DFT calculations revealed that the molecular lengths (*l*) for **M5**, **P1**, and **P3** were 24.2 Å, 22.4 Å, and 22.4 Å, respectively, when the flexible chains were in the all-*trans* configuration and fully extended. Consequently, the *d*_LC_/*l* ratios for **M5**, **P1**, and **P3** were calculated to be 1.0, 1.9, and 2.0, respectively. This indicates that the cybotactic clusters in the N phase of **M5** form a monolayer structure, while those in **P1** and **P3** form a double-layer structure (Figure 2b).

In the SmA phase of **M6** (80 °C), **P3** (30 °C), and **P4** (30 °C), broad scattering peaks were observed in the wide-angle region, while sharp peaks appeared in the small-angle region. The azimuthal profiles of diffraction in the small-angle region for **M6**, **P3**, and **P4** (Figures S22, S31, and S34) showed that the scattering in the small-angle region was orthogonal to that in the wide-angle region, a characteristic of the SmA phase. The 2*θ*_small_ values for **M6**, **P3**, and **P4** were 2.97°, 1.61°, and 1.61°, respectively, corresponding to *d*_LC_ values of 29.8 Å, 55.1 Å, and 55.1 Å. DFT calculations revealed *l* = 30.2 Å, 28.9 Å, and 28.9 Å for **M6**, **P3**, and **P4**, respectively, from which the *d*_LC_/*l* ratios were determined to be 1.0, 1.9, and 1.9. This suggests that **M6** has a monolayer structure in the SmA phase, while **P3** and **P4** exhibit double-layer structures (Figure 2b).

In summary, **M5** and **M6** exhibit monolayer structures in the LC phase, while **P1**–**P4** form double-layer structures. The formation of a double-layer structure is commonly reported in SCLCPs [64,65]. The 2*θ*_wide_ values observed in the N phase of **M5**, **P1**, and **P2** were 18.5°, 18.9°, and 19.3°, respectively, while those observed in the SmA phase of **M6**, **P3**, and **P4** were 18.5°, 18.9°, and 19.3°, respectively. Consequently, *d*_mesogen_ values for **M5**, **P1**, **P2**, **M6**, **P3**, and **P4** were calculated to be 4.78 Å, 4.71 Å, 4.85 Å, 4.78 Å, 4.60 Å, and 4.69 Å, respectively. Although there was no difference between the *d*_mesogen_ values of **M5** and **M6**, the values of **P3** and **P4** were 0.02–0.25 Å smaller than those of **P1** and **P2**.

### 2.4. Birefringence Properties

To evaluate the optical properties of **P1**–**P4**, temperature-variable birefringence (Δ*n*) measurements were performed following our previously reported method [66,67,68,69,70]. For comparison with the corresponding polymers, ∆*n* measurements were also conducted for **M5** and **M6** because of the lack of heat stability of **M1**–**M4**. First, the nematic liquid crystals (NLCs) were filled into homogeneously aligned polyimide cells. When preparing LC cells for **P1**–**P4**, the acrylic polymers **P1** and **P3** could be filled into the polyimide cells via capillary force; however, the methacrylic polymers **P2** and **P4** could not be filled due to their high viscosity, likely caused by differences in molecular weight. To confirm uniaxial orientation, POM observations were carried out. The prepared LC cells appeared dark when the polarizer was aligned with the rubbing direction and brightest when at 45° to the rubbing direction (Figure S37). These results confirm that the nematic director was aligned with the rubbing direction.

Next, we employed a micro-spectroscopic method, observing the transmitted light through the LC cell as a function of wavelength (*λ*) under cross-polarized conditions. The nematic director was set at 45° to the polarizer. A typical transmission light plot is shown in Figure S38 (dot). The transmitted light intensity (*I*) was fitted using the following Equation (1) and Cauchy’s Equation (2) to determine the coefficients *a*, *b*, and *c*, where *A* is a constant.

(1) $$\frac{I}{I_{0}}=A\sin^{2}\left(\frac{\pi ∆nd}{\lambda }\right)\sin^{2}\theta$$

(2) $$∆n=a+\frac{b}{\lambda^{2}}+\frac{c}{\lambda^{4}}$$

**Figure 3:**
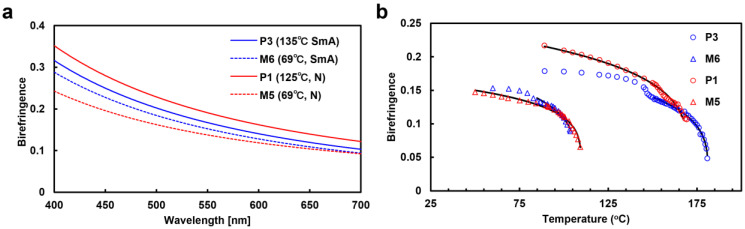
Birefringence is dependent on (**a**) wavelength and (**b**) temperature for **M5**, **M6**, **P1**, and **P3**. (**a**) Measured at the same reduced temperature (*T*/*T_i_* = 0.9) in each LC phase. (**b**) Plotted against temperature at 550 nm and fitted by Equations (3) and (4). For **M6** and **P3**, the fitting was performed within the nematic phase.

The theoretical curve obtained from Equation (2) fitted well to the transmitted light plot (Figure S38 (solid line)). From this fitting, we obtained the Δ*n* data presented in Figure 3. Figure 3a illustrates the wavelength dependence of Δ*n* at *T*/*T_i_* = 0.9 (*T_i_*; isotropic temperature), while Figure 3b shows Δ*n* plotted against temperature for **M5**, **M6**, **P1**, and **P3** at 550 nm. As shown in Figure 3a, the Δ*n* values of **P1** and **P3** were higher than those of **M5** and **M6**, indicating that Δ*n* increased as a result of polymerization. Additionally, **P1** and **P3** exhibited Δ*n* values of 0.24 and 0.20 at room temperature (25 °C), respectively.

As illustrated in Figure 3b, Δ*n* decreased with increasing temperature. The relationship between Δ*n* and temperature can be explained by the temperature dependence of the order parameter (*S*) of the nematic director, which is described by Haller’s approximation:

(3) $$∆n=∆n_{0}S$$

(4) $$S={\left(1-\frac{T}{T_{i}}\right)}^{\beta }$$

where Δ*n*_0_ is the extrapolated value for the perfectly oriented birefringence (*S* = 1) of the N LC, *T_i_* is the clearing point, and *β* is a material constant characteristic of the NLC. The theoretical curve obtained from Equation (4) fitted well to the plot of Δ*n* for each compound (Figure 3), and the values of *β* and Δ*n*_0_ were determined. Additionally, the order parameter *S* was calculated using Equation (3) from the measured Δ*n* values and the corresponding Δ*n*_0_ values.

The values of *S* at 90 °C were calculated, as this was the temperature at which **M5**, **M6**, **P1**, and **P3** could be measured, and are shown in Table 4 along with the corresponding values of *β* and Δ*n*_0_. The Δ*n*_0_ values for **M5**, **M6**, **P1**, and **P3** were 0.21, 0.26, 0.31, and 0.26, respectively, and the *β* values were 0.18, 0.23, 0.22, and 0.23, respectively. The *S* values at 90 °C were 0.60, 0.50, 0.69, and 0.69, respectively. Comparison of these values indicates an improvement in *S* due to the polymer effect [21]. 

Furthermore, a comprehensive evaluation of the *S* values, combined with the results from DSC and WAXD measurements, led to the conclusion that the significant increase in the higher phase transition temperatures (SmA–N and N–isotropic transitions) of **P1**–**P4** might be attributed to the enhancement of *S*, which resulted from the formation of a double-layer structure in the liquid crystalline phase.

### 2.5. Fluorescence Properties

To investigate the fluorescence properties of **M1**–**M4** and **P1**–**P4**, absorption spectra, fluorescence spectra, and fluorescence quantum yields (*Φ*_fl_) were measured in a dilute THF solution and in the solid state. Additionally, the fluorescence spectra of cast films of **P1**–**P4** were measured. The obtained values for maximum absorption wavelength (*λ*_abs_), maximum fluorescence wavelength (*λ*_fl_), and *Φ*_fl_ for **M1**–**M4** and **P1**–**P4** are summarized in Table 5. 

**Figure 4:**
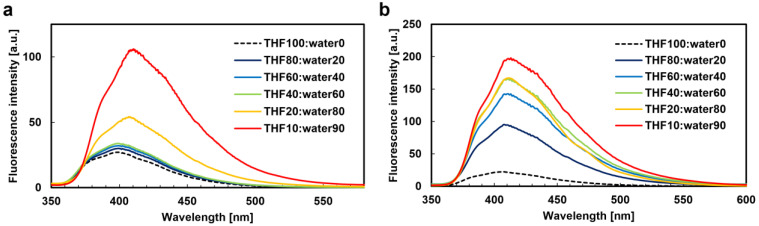
Aggregation experiments. Fluorescence spectra of (**a**) **M2** and (**b**) **P2** in THF/water mixtures with different water contents (vol %) excited at each *λ*_abs_; the concentration is 10^−5^ M.

The *λ*_abs_ values for all the monomers (**M1**–**M4**) in THF were 316 nm. The *Φ*_fl_ values for **M1**–**M4** in THF solution were 0.02, while in the solid state, they were 0.43, 0.58, 0.61, and 0.48, respectively. This indicates that **M1**–**M4** exhibited aggregation-induced emission (AIE) properties. The AIE properties were further confirmed by aggregation experiments on **M2** and **P2** (Figure 4). The *λ*_fl_ values for **M1**–**M4** in the THF solution were approximately 400 nm, while in the solid state (polycrystalline state), they were 417, 407, 413, and 408 nm, respectively. These results suggest that the fluorescence in the solid state may be derived from aggregates rather than isolated monomers. This behavior was similar to that of our previously reported AIEgen, bridged stilbene [55].

**Figure 5:**
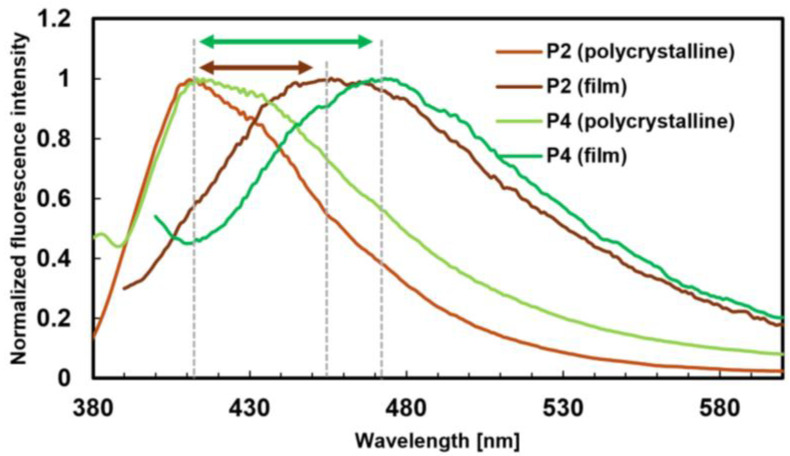
Fluorescence spectra of **P2** and **P4** excited at 370 nm (polycrystalline) and at 380 nm (film).

The *λ*_abs_ value for **P1**–**P4** in THF was 316 nm. The *Φ*_fl_ value for **P1**–**P4** in the THF solution was approximately 0.03, while in the solid state, the values were 0.14, 0.15, 0.35, and 0.18, respectively. **P1**–**P4** exhibited the same AIE properties as the monomers, and aggregation experiments with **P2** and **P4** yielded similar results to those of the monomers (Table S3). Similar redshifts in the *λ*_fl_ values for **P1**–**P4** in both the THF solution and the solid state were observed, mirroring the behavior of the monomers. These findings revealed that the luminescence properties of the bulk polymers and monomers were nearly identical. Interestingly, the *λ*_fl_ values for **P1**–**P4** in the polymer films were 442, 456, 469, and 473 nm, respectively, redshifted by more than 27 nm compared with those in the solid state (polycrystalline state) (Figure 5). The relationship between the *λ*_fl_ of the films and the liquid crystalline phase at room temperature was also investigated. The *λ*_fl_ values for **P1** and **P2** (which exhibited the N phase) were around 450 nm, while those for **P3** and **P4** (which exhibited the SmA phase) were around 470 nm. These results suggest that the *λ*_fl_ in the films may vary depending on the liquid crystalline phase, with a relatively shorter wavelength shift in the N phase and a longer wavelength shift in the SmA phase. This indicates that the molecular orientation and interactions in different LC phases may affect the fluorescence properties.

## 3. Experimental

### 3.1. Materials

Unless otherwise noted, all solvents and chemicals were commercially available and used without further purification. Column chromatography was performed on silica gel (Silica Gel 60N, 63–210 μm, Kanto chemical Co., Inc., Tokyo, Japan). [Hydroxy(tosyloxyl)iodo]benzene, tetrakis(triphenylphoshine)palladium (0) (Pd(PPh_3_)_4_), [1,1-bis(diphenylphosphino)ferrocene]palladium (0) (Pd(dppf)Cl_2_·CH_2_Cl_2_), and 6-bromo-1-tetralone were purchased from TCI (Tokyo, Japan).

### 3.2. Instruments

^1^H-NMR and ^13^C-NMR spectra were recorded on BRUKER 500 (Yokohama, Japan) (500 MHz) and JEOL 400 (Tokyo, Japan) (100 MHz) spectrometers, respectively, for CDCl_3_ solution using tetramethylsilane (TMS) as an internal standard. ^1^H-NMR spectra were reported as follows: chemical shift (δ ppm), multiplicity (s = singlet, d = doublet, t = triplet, q = quartet, m = multiplet), integration, and coupling constants in units of Hz. ^13^C-NMR spectra were reported as chemical shifts in ppm. The FT-IR spectra were recorded on a JASCO FT-IR 4600 spectrometer (Tokyo, Japan). High-resolution EI mass spectra (HRMS) were recorded on a double-focusing mass spectrometer JEOL JMS-700, measured at the Tokyo Institute of Technology Open Facility Center. This center is independent of our laboratory to ensure fairness. Size exclusion chromatography (SEC) was performed using a JASCO system (PU-2080, CL-NETII/ADC, CO-2060, UV-2075, RI-2031, Tokyo, Japan) equipped with TSK gel columns (TOSOH G3000H xl, Tokyo, Japan) with THF as the eluent at the following rate of 0.85 mL min^−1^ at 40 °C after calibration with polystyrene standards. Polarized optical microscopy (POM) was performed using a Leica DM2500P microscopy (Wetzlar, Germany) with a Mettler FP90 hot stage (Greifensee, Switzerland). Differential scanning calorimetry (DSC) was performed using PerkinElmer DSC 8500 equipment (Waltham, MA, USA) at a scanning rate of 10 °C min^−1^ under a flow of dry nitrogen. Thermo-gravimetric analysis (TGA) was performed using a Rigaku Thermo Plus EVO2 series TG-DTA 8122 (Tokyo, Japan) at a heating rate of 20 °C min^–1^ under a flow of dry nitrogen. The initial mass of the samples was 3–6 mg. X-ray investigations were carried out with samples kept in glass capillary tubes (1.0 mm diameter) for oriented patterns under a magnetic field. Wide-angle X-ray diffraction (WAXD) patterns were obtained using a Bruker D8 DISCOVER (Billerica, MA, USA) equipped with a Vantec-500 detector and Cu Kα radiation. UV-Vis spectra were recorded on a JASCO V-670 UV-vis spectrophotometer (Tokyo, Japan) and fluorescence spectra were recorded on a JASCO FP-6500 spectrofluorometer (Tokyo, Japan). Absolute quantum yields were measured by Hamamatsu Photonics Quantaurus QY apparatus (Hamamatsu City, Japan). All sample solutions were de-aerated by bubbling with argon for 15 min prior to the quantum yield measurement.

### 3.3. Birefringence

Measurement of birefringence was performed in uniaxially aligned LC cells containing indium tin oxide (ITO) purchased from EHC. The cell gap (*d*) of 3–5 μm was determined by the interferometric method [66,67,68,69,70].

### 3.4. Theoretical Calculations

Theoretical calculations were carried out on the Gaussian 16 program [71]. Geometry optimizations were carried out using DFT methods at the B3LYP with the 6-31G(d) basis. Whether the optimized geometry was at the stationary point without any imaginary frequency was checked by the frequency calculation performed at the optimized geometries using their level of theory.

### 3.5. Synthesis

6-(4-ethylphenyl)-3,4-dihydronaphthalen-1(2*H*)-one (**6a**)

To a solution of 6-bromo-1-tetralone (**5**) (3.4 g, 15 mmol), 4-alkylphenylboronic acid (3.4 g, 22 mmol), potassium phosphate (9.5 g, 45 mmol) in solvent (30 mL/12 mL/6 mL; toluene/water/methanol), Pd(PPh_3_)_4_ (0.55 g, 0.48 mmol) was added under argon atmosphere, and the mixture was refluxed (100 °C) for 3 hours. After the reaction, the mixture was cooled to room temperature, then extracted with dichloromethane. The organic layer was washed with water three times, dried over MgSO_4_, filtrated, and evaporated in vacuo. The residue was purified by column chromatography on silica gel (6/1 (*v*/*v*) hexane/ethyl acetate) to afford **6a** as a brown solid; yield 96%; ^1^H-NMR (500 MHz, CDCl_3_) δ 8.09 (d, *J* = 8.2 Hz, Ar-*H*, 1H), 7.56–7.52 (m, Ar-*H*, 3H), 7.47–7.45 (m, Ar-*H*, 1H), 7.31–7.29 (m, Ar-*H*, 2H), 3.02 (t, *J* = 6.1 Hz, -C*H*_2_-, 2H), 2.73–2.67 (m, -C*H*_2_-, 4H), 2.20–2.15 (m, -C*H*_2_-, 2H), 1.28 (t, *J* = 7.6 Hz, -C*H*_3_, 3H) ppm (Figure S47).

6-(4-ethylphenyl)-1-methylene-1,2,3,4-tetrahydronaphthalene (**7a**)

Methyltriphenylphosphonium bromide (7.1 g, 19 mmol) was dissolved in THF (40 mL) under argon atmosphere. The solution was cooled to 0 °C, and then potassium tert-butoxide (2.4 g, 21 mmol) was added. Following stirring for 10 minutes, **6a** (14.5 mmol) was added, warming to room temperature and stirring overnight. The reaction mixture was quenched by adding NH_4_Cl aq to the solution and extracted with ethyl acetate. The organic layer was washed with water three times, dried over MgSO_4_, filtrated, and evaporated in vacuo. The residue was purified by column chromatography on silica gel (4/1 (*v*/*v*) hexane/dichloromethane) to afford **7a** as a colorless solid; yield 90%; ^1^H-NMR (500 MHz, CDCl_3_) δ 7.71 (d, *J* = 8.2 Hz, Ar-*H*, 1H), 7.51 (d, *J* = 8.2 Hz, Ar-*H*, 2H), 7.40–7.37 (m, Ar-*H*, 1H), 7.33–7.32 (m, Ar-*H*, 1H), 7.26 (d, *J* = 7.6 Hz, Ar-*H*, 2H), 5.51 (s, C=C*H*, 1H), 4.96 (s, C=C*H*, 1H), 2.90 (t, *J* = 6.3 Hz, -C*H*_2_-, 2H), 2.69 (q, *J* = 7.6 Hz, -C*H*_2_-, 2H), 2.57 (t, *J* = 6.1 Hz, -C*H*_2_-, 2H), 1.94–1.89 (m, -C*H*_2_-, 2H), 1.27 (t, *J* = 7.6 Hz, -C*H*_3_, 3H) ppm (Figure S49).

2-(4-ethylphenyl)-5,7,8,9-tetrahydro-6*H*-benzo[7]annulen-6-one (**8a**)

To a solution of **7a** (13 mmol) in solvent (38 mL/2 mL; methanol/water), HTIB ([hydroxy(tosyloxy)iodo]benzene; 5.6 g, 14 mmol) was added under air, and stirred at room temperature for 20 min. After the reaction, the mixture was extracted with dichloromethane. The organic layer was washed with water three times, dried over MgSO_4_, filtrated, and evaporated in vacuo. The residue was purified by column chromatography on silica gel (6/1 (*v*/*v*) hexane/ethyl acetate) to afford **8a** as a colorless solid; yield 92%; ^1^H-NMR (500 MHz, CDCl_3_) δ 7.51 (d, *J* = 8.2 Hz, Ar-*H*, 2H), 7.41–7.38 (m, Ar-*H*, 2H), 7.27 (d, *J* = 8.2 Hz, Ar-*H*, 2H), 7.21 (d, *J* = 7.6 Hz, Ar-*H*, 1H), 3.76 (s, -C*H*_2_-, 2H), 3.01 (t, *J* = 6.4 Hz, -C*H*_2_-, 2H), 2.69 (q, *J* = 7.6 Hz, -C*H*_2_-, 2H), 2.60 (t, *J* = 7.0 Hz, -C*H*_2_-, 2H), 2.06–2.01 (m, -C*H*_2_-, 2H), 1.28 (t, *J* = 7.6 Hz, -C*H*_3_, 3H) ppm (Figure S51).

3-(4-ethylphenyl)-6,7-dihydro-5*H*-benzo[7]annulen-8-yl trifluoromethanesulfonate (**9a**)

The mixture of **8a** (12 mmol) and THF (30 mL) was cooled to −20 °C, and then tert-butoxide (2.4 g, 21 mmol) was added. The mixture was stirred at 0 °C for 1 h, and then cooled to −20 °C, then N-phenylbis(trifluolomethanesulfonimide) (5.3 g, 15 mmol) was added, and stirred at −20 °C for a further 1 h, and then warmed to 0 °C, and stirred for 4 h. After that, the reaction was quenched by the dropwise addition of water (20 mL), and organic products were extracted with ethyl acetate. The organic layer was washed with water three times, dried over MgSO_4_, filtrated, and evaporated in vacuo. The residue was purified by column chromatography on silica gel (6/1 (*v*/*v*) hexane/ethyl acetate) to afford **9a** as a slight yellow solid; yield 98%; ^1^H-NMR (500 MHz, CDCl_3_) δ 7.51 (d, *J* = 7.9 Hz, Ar-*H*, 2H), 7.43–7.40 (m, Ar-*H*, 1H), 7.34–7.33 (m, Ar-*H*, 1H), 7.27 (d, *J* = 8.2 Hz, Ar-*H*, 2H), 7.22 (d, *J* = 7.9 Hz, Ar-*H*, 1H), 6.62 (s, Ar-C*H*-, 1H), 2.95 (t, *J* = 5.2 Hz, -C*H*_2_-, 2H), 2.81 (t, *J* = 6.4 Hz, -C*H*_2_-, 2H), 2.70 (q, *J* = 7.6 Hz, -C*H*_2_-, 2H), 2.06–2.01 (m, -C*H*_2_-, 2H), 1.28 (t, *J* = 7.6 Hz, -C*H*_3_, 3H) ppm (Figure S53).

*tert*-butyl(2-(4-(3-(4-ethylphenyl)-6,7-dihydro-5*H*-benzo[7]annulen-8-yl)phenoxy)ethoxy)dimethylsilane (**10a**)

To a solution of 9 (8 mmol), 4a (16 mmol), K_3_PO_4_·*n*H_2_O (2.4 g, 16 mmol) in THF (40 mL), Pd(PPh_3_)_4_ (0.28 g, 0.24 mmol) was added under argon atmosphere, and stirred at 50 °C overnight. After the reaction, the mixture was cooled to room temperature, then extracted with dichloromethane. The organic layer was washed with water three times, dried over MgSO_4_, filtrated, and evaporated in vacuo. The residue was purified by column chromatography on silica gel (4/1 (*v*/*v*) hexane/dichloromethane) to afford **10a** as a colorless solid; yield 54%; ^1^H-NMR (500 MHz, CDCl_3_) δ 7.55 (d, *J* = 7.9 Hz, Ar-*H*, 2H), 7.45–7.39 (m, Ar-*H*, 4H), 7.27–7.26 (m, 3H), 6.91 (d, *J* = 8.5 Hz, Ar-*H*, 2H), 6.77 (s, Ar-*H*, 1H), 4.07 (t, *J* = 5.3 Hz, -C*H*_2_-, 2H), 3.99 (t, *J* = 5.0 Hz, -C*H*_2_-, 2H), 2.87 (t, *J* = 6.0 Hz, -C*H*_2_-, 2H), 2.72–2.66 (m, -C*H*_2_-, 4H), 2.23 (t, *J* = 6.3 Hz, -C*H*_2_-, 2H), 1.28 (t, *J* = 7.6 Hz, -C*H*_3_, 3H), 0.92 (s, C*H*_3_, 9H), 0.12 (s, -C*H*_3_, 6H) ppm (Figure S55).

2-(4-(3-(4-ethylphenyl)-6,7-dihydro-5*H*-benzo[7]annulen-8-yl)phenoxy)ethan-1-ol (**11a**)

To a solution of **10a** (2.6 mmol) in THF (5.0 mL), 12M HCl aq (4.0 mL) was added, and stirred at room temperature for 10 min. After that, the reaction was quenched by the dropwise addition of NaHCO_3_ aq (20 mL), and organic products were extracted with dichloromethane. The organic layer was washed with water three times, dried over MgSO_4_, filtrated, and evaporated in vacuo. Purification by recrystallization (CHCl_3_/Hex) gave **11a** as a colorless solid; yield 87%; ^1^H-NMR (500 MHz, CDCl_3_) δ 7.55 (d, *J* = 8.2 Hz, Ar-*H*, 2H), 7.46 (d, *J* = 8.7 Hz, Ar-*H*, 2H), 7.42 (dd, *J* = 7.8, 2.0 Hz, Ar-*H*, 1H), 7.39 (s, Ar-*H*, 1H), 7.28–7.26 (m, 3H), 6.93 (d, *J* = 8.9 Hz, Ar-*H*, 2H), 6.78 (s, Ar-*H*, 1H), 4.12 (t, *J* = 4.6 Hz, -C*H*_2_-, 2H), 4.00–3.97 (m, -C*H*_2_-, 2H), 2.88 (t, *J* = 6.1 Hz, -C*H*_2_-, 2H), 2.72–2.66 (m, -C*H*_2_-, 4H), 2.26–2.21 (m, -C*H*_2_-, 2H), 1.28 (t, *J* = 7.5 Hz, -C*H*_2_-, 3H) ppm (Figure S57).

2-(4-(3-(4-ethylphenyl)-6,7-dihydro-5*H*-benzo[7]annulen-8-yl)phenoxy)ethyl acrylate (**M1**)

To a solution of compound **11a** (1.3 mmol) and triethylamine (0.27 mL, 2.0 mmol) in dichloromethane (5.0 mL) was added acyl chloride (1.7 mmol) and the mixture was stirred at room temperature for 1 h. After the reaction, the solvent was removed by evaporation, and the residue was filtered with hexane. The organic layer was washed with water three times, dried over MgSO_4_, filtrated, and evaporated in vacuo. The residue was purified by column chromatography on silica gel (1/1 (*v*/*v*) hexane/ethyl acetate) to afford crude M1-M6. Purification by recrystallization (hexane/dichloromethane) gave pure **M1** as a colorless solid; yield: 53%; ^1^H-NMR (500 MHz, CDCl_3_) δ 7.55 (d, *J* = 8.2 Hz, Ar-*H*, 2H), 7.47–7.39 (m, Ar-*H*, 4H), 7.27–7.25 (m, 3H), 6.93 (d, *J* = 8.5 Hz, Ar-*H*, 2H), 6.77 (s, Ar-*H*, 1H), 6.48–6.44 (m, =C*H*, 1H), 6.18 (dd, *J* = 17.4, 10.4 Hz, -C*H*=, 1H), 5.87 (dd, *J* = 10.5, 1.4 Hz, =C*H*, 1H), 4.53 (t, *J* = 4.7 Hz, -C*H*_2_-, 2H), 4.25 (t, *J* = 4.9 Hz, -C*H*_2_-, 2H), 2.88 (t, *J* = 6.1 Hz, -C*H*_2_-, 2H), 2.72–2.66 (m, -C*H*_2_-, 4H), 2.23 (t, *J* = 6.1 Hz, -C*H*_2_-, 2H), 1.28 (t, *J* = 7.6 Hz, -C*H*_3_, 3H) ppm (Figure S59); ^13^C-NMR (100 MHz, CDCl_3_) δ 166.2, 157.9, 143.4, 142.3, 141.6, 139.2, 138.4, 137.4, 136.4, 131.5, 131.1, 128.4, 128.2, 127.7, 127.5, 127.4, 127.0, 124.5, 114.5, 66.1, 63.0, 35.0, 33.1, 30.3, 28.6, 15.7 ppm (Figure S60). HRMS (EI) Calcd for C_30_H_30_O_3_: 438.5664, Found 438.2195 (Figure S57).

Poly[2-(4-(3-(4-ethylphenyl)-6,7-dihydro-5*H*-benzo[7]annulen-8-yl)phenoxy)ethyl] acrylate (**P1**)

To a pressure-resident tube, which contained **M1** (0.37 g, 0.83 mmol) and a portion of THF (3.0 mL), AIBN (azobis(isobutyronitrile), 9.0 mg, 6.5 wt%, was added, and the mixture was stirred at 60 °C for 24 h. The mixture was poured dropwise with an excess amount of methanol, and it was filtered. The solids were purified by column chromatography on Sephadex^TM^ G100 (eluted with THF), and reprecipitated with methanol; colorless solid; yield 40.3%; ^1^H-NMR (500 MHz, CDCl_3_) δ 7.50–7.05 (brm, Ar-H, 9H), 6.88–6.71 (brm, Ar-H, 3H), 4.26–4.10 (brm, 4H), 2.88–2.36 (brm, 6H), 2.17–2.13 (brm, 2H), 1.84 (br, 1H), 1.26 (br, 3H) ppm (Figure S71); ^13^C-NMR (100 MHz, CDCl3) δ 174.7, 157.8, 143.3, 142.1, 141.6, 139.0, 138.3, 138.2, 137.2, 136.3, 135.9, 131.2, 128.3, 127.4, 127.3, 126.9, 125.6, 124.5, 114.5, 65.8, 62.9, 41.3, 41.2, 35.0, 34.3, 33.1, 31.6, 30.4, 30.1, 29.6, 28.6, 21.3, 15.7 ppm (Figure S72).

## 4. Conclusions

We synthesized **P1**–**P4** as SCLCPs with varying flexible chain lengths by radical polymerization of acrylate monomers containing π-extended bridged stilbene mesogens. The resulting polymers exhibited liquid crystalline phases over a temperature range exceeding 150 °C. **P1** and **P2** exhibited a nematic phase at room temperature, which was maintained for more than three months. These stable nematic phases are highly advantageous for device applications operating at room temperature. WAXD measurements revealed that the polymers possessed a double-layer structure in the liquid crystalline phase. Additionally, the polymers demonstrated larger order parameter (*S*) values compared with small molecules and exhibited higher birefringence. The polymers were also found to retain the AIE characteristics of the **DPB[7]** skeleton, with high quantum yields (*Φ*_fl_ = 0.14–0.35) in the solid state (polycrystalline). Furthermore, film formation resulted in a redshift of the maximum fluorescence wavelength (*λ*_fl_) by more than 27 nm, yielding *λ*_fl_ values in the range of 442–473 nm. The magnitude of the redshift may be related to the liquid crystalline phase exhibited at room temperature, though further investigation is required to clarify the details. These optically and luminescent-functionalized SCLCPs hold promise for applications in luminescent semiconductors and optoelectronic materials, including holograms [72,73,74] and polarized light-emitting devices [75,76,77,78,79,80,81,82,83,84,85]. 

## Figures and Tables

**Table 1:** The results of polymerization: yields, *M*_n_, *M*_w_, polydispersity indexes (*M*_w_/*M*_n_), and degradation temperature at 10% weight loss (*T*_10_) of **P1**–**P4**.

Entry	Yield (%)	*M*_n_	*M*_w_	*M*_w_/*M*_n_	*T*_10_ [°C]
**P1**	40	3000	5200	1.70	410
**P2**	34	10,400	20,000	1.93	409
**P3**	19	5000	7000	1.40	413
**P4**	10	17,100	26,500	1.55	412

**Table 2:** Phase transition temperature [°C] (enthalpy Δ*H* [kJ mol^–1^]) and the range of LC phase (Δ*T*_LC_ [°C]) of **DPB[7]** monomers (**M5** and **M6**) and **DPB[7]** polymers (**P1**–**P4**) upon heating and cooling at a rate of 10 °C min^–1^ (Cr and Cr2: crystal phase, N: nematic phase, SmA: smectic A phase, Iso: isotropic phase, G: glass state).

Entry	Phase Transition Behavior	
	Heating ^[a]^	Cooling ^[b]^
**DPB[7]-C3 ^[c]^**	Cr 52.6 (11.8) N 160.4 (0.85) Iso	Iso 158.3 (0.86) N 4.8 (5.68) Cr
**M5**	Cr 37.9 (0.15) Cr2 ^[d]^ 66.4 (0.24) N 111.6 (0.33) Iso	Iso 109.8 (0.60) N
**M6**	Cr 77.4 (22.5) SmA 84.8 (0.49) N 109.3 (0.77) Iso	Iso 107.5 (0.41) N 83.0 (0.49) SmA 29.5 (15.6) Cr
**P1**	G 60.6 N 176.3 (0.22) Iso	Iso 173.4 (0.36) N 57.5 G
**P2**	N 205.0 (0.32) Iso	Iso 202.5 (0.28) N
**P3**	G 25.6 SmA 150.3 (0.65) N 184.2 (0.32) Iso	Iso 181.8 (0.46) N 152.8 (1.28) SmA 23.2 G
**P4**	SmA 193.8 (0.75) N 205.0 (0.19) Iso	Iso 202.0 (0.44) N 181.5 (1.18) SmA

^[a]^ Third heating for **DPB[7]-C3** and **P1–P4**, and second heating for **M5** and **M6**. ^[b]^ Third cooling for DPB[7]-C3 and **P1–P4** and second cooling for **M5** and **M6**. ^[c]^ Reported by Iwai et al. [54]. ^[d]^ A crystal–crystal phase transition was observed only for **M5**.

**Table 3:** The 2*θ* peak angle in small-angle region (2*θ*_small_ [°]) and wide-angle region (2*θ*_wide_ [°]) obtained from WAXD profile, their 2*θ d*-spacing values (*d*_LC_ and *d*_mesogen_ [Å]), calculated molecular length (*l* [Å]), and the value of *d*_LC_/*l*.

Entry	2*θ*_small_ [°]	*d*_LC_^[a]^ [Å]	*l*^[b]^ [Å]	*d*_LC_/*l* [-]	2*θ*_wide_ [°]	*d*_mesogen_ [Å]
**M5**	3.60 ^[c]^	24.6	24.2	1.0	18.5 ^[c]^	4.78
**P1**	2.09	42.4	22.4	1.9	18.9 ^[d]^	4.71
**P2**	2.01 ^[e]^	44.0	22.4	2.0	18.3 ^[e]^	4.85
**M6**	2.97 ^[c]^	29.8	30.2	1.0	18.5 ^[c]^	4.78
**P3**	1.61 ^[e]^	55.1	28.9	1.9	19.3 ^[e]^	4.60
**P4**	1.61 ^[e]^	55.1	28.9	1.9	18.9 ^[e]^	4.69

^[a]^ Layer spacing of LC phase (nematic phase for **M5**, **P1**, **P2**, and smectic A phase for **M6**, **P3**, **P4**) calculated from 2*θ*_small_ [°] value. ^[b]^ Whole molecular length (or length of side-chain for polymers) when alkyl chains are assumed to be fully extended in all-*trans* (calculated using DFT calculation at the B3LYP/6-31G(d) level of theory). ^[c]^ Measured at 80 °C. ^[d]^ Measured at 40 °C. ^[e]^ Measured at 30 °C.

**Table 4:** Determined values of ∆*n*_0_ and *β* by Haller’s approximation and estimated birefringence (∆*n*) and order parameter (*S*) at 90 °C.

Entry	∆*n*_0_	*β*	∆*n* (90 °C)	*S* (90 °C)
**M5**	0.21	0.18	0.12	0.60
**M6**	0.26	0.23	0.13 ^[a]^	0.50 ^[b]^
**P1**	0.31	0.22	0.22	0.69
**P3**	0.26	0.23	0.18 ^[a]^	0.69 ^[b]^

^[a]^ Value in smectic A phase. ^[b]^ calculated using Δ*n* (90 °C) value in smectic A phase.

**Table 5:** Spectroscopic properties for **M1**–**M4** and **P1**–**P4** in THF solution, solid state, and film.

Entry	*λ*_abs_ [nm]	*Φ*_fl_ [-]		*λ*_fl_ [nm]		
	THF	THF	Solid ^[a]^	THF	Solid ^[a]^	Film
**M1**	316	0.02	0.43	399	417	-
**M2**	316	0.02	0.58	399	407	-
**M3**	316	0.02	0.61	401	413	-
**M4**	316	0.02	0.48	400	408	-
**P1**	316	0.03	0.14	407	415	442
**P2**	316	0.02	0.16	399	413	456
**P3**	316	0.03	0.35	400	413	469
**P4**	316	0.03	0.18	407	413	473

^[a]^ Polycrystalline solid.

## Data Availability

The original contributions presented in this study are included in this article/Supplementary Materials, further inquiries can be directed to the corresponding author.

## Supplementary Materials

The following supporting information can be downloaded at https://www.mdpi.com/article/10.3390/molecules29215220/s1: Figures S1–S4: TGA curves; Figures S5–S10: DSC thermogram; Figures S11–S16: POM images; Figures S17–S34: 1D- or 2D-WAXD profiles; Figures S35 and S36: Optimized structure (DFT calculation); Figure S37: POM images of nematic phase in polyimide cell; Figure S38: Wavelength dependence of light transmittance; Figure S39: Absorption spectra; Figure S40: Fluorescence spectra; Figures S41–S78: NMR spectra; Figures S79–S84: FT-IR spectra; Figures S85–S88: HRMS spectra; Tables S1 and S2: Atomic coordination and absolute energy (DFT calculation); Table S3: Aggregation experiments.
