# Supplementary material for: Synthesis of Side-Chain Liquid Crystalline Polyacrylates with Bridged Stilbene Mesogens
Source: Molecules. 2024 Nov 4;29(21):5220. doi: 10.3390/molecules29215220 (PMC11547409; doi:10.3390/molecules29215220)
Supplement: Supplementary file 1 [file molecules-29-05220-s001.zip › molecules-3261235-supplementary.pdf]

Synthesis of side-chain liquid crystalline polyacrylates with bridged stilbene mesogens

Gen-ichi Konishi, Yuki Sawatari, Riki Iwai, Takuya Tanaka, Yoshimichi Shimomura and Masatoshi Tokita

Table of contents

1. Experimental section 2
2. (Thermal gravimetric analysis) TGA charts 12
3. Liquid crystal behavior 14
(differential scanning calorimetry) DSC thermogram. 14
(Polarized optical microscopy) POM images 17
(wide angle x-ray diffraction) WAXD measurement 21
4. Optical properties 32
Birefringence 32
Fluorescence properties 33
5. Spectra chart 35
NMR chart 35
FT-IR spectra 73
High resolution mass spectrometry (HRMS) chart 77

1. *Experimental section*

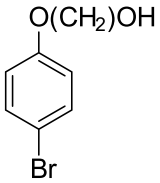

2-(4-bromophenoxy)ethan-1-ol (2a)

To a mixture of 4-bromophenol (1.7 g, 10 mmol), K_2CO_3 (4.1 g, 30 mmol) in 2-butanone (30 ml) under argon atmosphere, 2-bromo-1-ethanol (1.1 ml, 15 mmol) was added, and the mixtures was refluxed (100 °C) for 24h. After the reaction, the mixture was cooled to room temperature, then extracted with dichloromethane. The organic layer was washed with water three times, dried over $MgSO_4$, filtrated, and evaporated in vacuo. The residue was purified by column chromatography on silica gel (1/1 (v/v) hexane/ethyl acetate) to afford **2a** as colorless oil. Yield 25%; ^{1}H-NMR (500 MHz, $CDCl_3$) δ 7.40-7.37 (m, Ar-*H*, 2H), 6.82-6.79 (m, Ar-*H*, 2H), 4.05 (t, *J* = 4.4 Hz, -C*H*$_2$-, 2H), 3.96 (dd, *J* = 9.2, -C*H*$_2$-, 5.2 Hz, 2H) ppm (Figure S41).

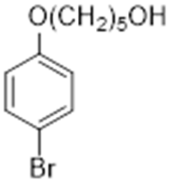

5-(4-bromophenoxy)pentan-1-ol (2b)

Following a similar procedure used for **2a** from **1** (1.7 g, 10 mmol), K_2CO_3 (3.5 g, 25 mmol), 2-butanone (15 ml), and 5-bromo-1-pentanol (1.2 ml, 10 mmol) chromatography over silica gel, eluting with 1/1 (v/v) hexane/ethyl acetate gave crude **2b** as colorless oil. Yield 68%; ^{1}H-NMR (500 MHz, $CDCl_3$) δ 7.36 (d, *J* = 9.2 Hz, Ar-*H*, 2H), 6.77 (d, *J* = 8.9 Hz, Ar-*H*, 2H), 3.93 (t, *J* = 6.4 Hz, -C*H*$_2$-, 2H), 3.68 (q, *J* = 5.8 Hz, -C*H*$_2$-, 2H), 1.84-1.78 (m, -C*H*$_2$-, 2H), 1.67-1.62 (m, -C*H*$_2$-, 2H), 1.57-1.53 (m, -C*H*$_2$-, 2H) ppm (Figure S42).

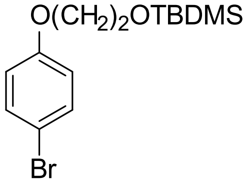

(2-(4-bromophenoxy)ethoxy)(*tert*-butyl)dimethylsilane (3a)

To a solution of **2a** (1.0g, 4.6 mmol) and imidazole (0.47 g, 7.0 mmol) in

dichloromethane (15 ml), *tert*-butyldimethylsilyl chloride (0.75 g, 5.0 mmol) was added under argon atmosphere and stirred at room temperature for 3 hours. After the reaction, the mixture was extracted with ethyl acetate. The organic layer was washed with water three times, and with NH_4Cl aq. dried over $MgSO_4$, filtrated, and evaporated in vacuo. The residue was purified by column chromatography on silica gel (1/1 (v/v) hexane/ethyl acetate) to afford **3a** as colorless oil. Yield 95%; ^{1}H-NMR (500 MHz, $CDCl_3$) δ 7.38-7.34 (m, Ar-*H*, 2H), 6.81-6.78 (m, Ar-*H*, 2H), 4.01-3.99 (m, -C*H*$_2$-, 2H), 3.96-3.94 (m, -C*H*$_2$-, 2H), 0.90 (s, -C*H*$_3$, 9H), 0.09 (s, -C*H*$_3$, 6H) ppm (Figure S43).

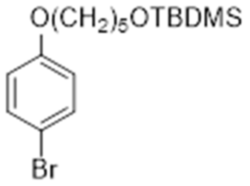

((5-(4-bromophenoxy)pentyl)oxy)(*tert*-butyl)dimethylsilane (3b)

Following a similar procedure used for **3a** from **2b** (1.8 g, 6.9 mmol), imidazole (0.7 g, 10 mmol), *tert*-butyldimethylsilyl chloride (1.6 g, 10 mmol)) chromatography over silica gel, eluting with 1/1 (v/v) hexane/ethyl acetate gave crude **3b** as colorless oil. Yield 91%; ^{1}H-NMR (500 MHz, $CDCl_3$) δ 7.39-7.36 (m, Ar-*H*, 2H), 6.80-6.77 (m, Ar-*H*, 2H), 3.92 (t, *J* = 6.6 Hz, -C*H*$_2$-, 2H), 3.63 (t, *J* = 6.3 Hz, -C*H*$_2$-, 2H), 1.82-1.76 (m, -C*H*$_2$-, 2H), 1.59-1.56 (m, -C*H*$_2$-, 2H), 1.52-1.48 (m, -C*H*$_2$-, 2H), 0.89 (s, -C*H*$_3$, 9H), 0.05 (s, -C*H*$_3$, 6H) ppm (Figure S44).

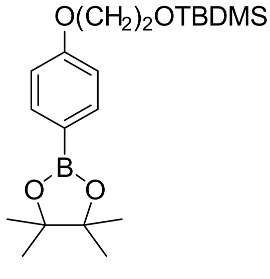

***tert*-butyldimethyl(2-(4-(4,4,5,5-tetramethyl-1,3,2-dioxaborolan-2-yl)phenoxy)ethoxy)silane (4a)**

To a mixture of **3a** (1.5 g, 4.6 mmol), potassium acetate (0.90 g, 9.2 mmol) in 1,4-dioxane (10 ml), bis(pinacolate)diboron (1.4 g, 5.5 mmol) was added, and the mixtures was refluxed (80 °C) for 3.5 hours. After the reaction, the mixture was cooled to room temperature, then extracted with dichloromethane. The organic layer was washed with water three times, dried over $MgSO_4$, filtrated, and

evaporated in vacuo. The residue was purified by column chromatography on silica gel (6/1 (v/v) hexane/ethyl acetate) to afford **4a** as colorless solid. Yield 71%; ^{1}H-NMR (500 MHz, $CDCl_3$) δ 7.74 (dd, *J* = 6.7, 1.8 Hz, Ar-*H*, 2H), 6.89 (dt, *J* = 8.9, 2.1 Hz, Ar-*H*, 2H), 4.06 (t, *J* = 5.2 Hz, -C*H*$_2$-, 2H), 3.98-3.95 (m, -C*H*$_2$-, 2H), 1.33 (s, -CH_3, 12H), 0.90 (s, -C*H*$_3$, 9H), 0.10 (s, -C*H*$_3$, 6H) ppm (Figure S45).

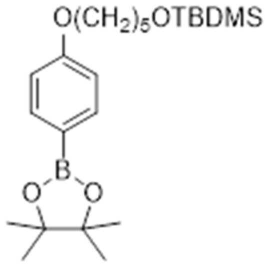

***tert*-butyldimethyl((5-(4-(4,4,5,5-tetramethyl-1,3,2-dioxaborolan-2-yl)phenoxy)pentyl)oxy)silane (4b)**

Following a similar procedure used for **4a** from **3b** (4.3 g, 12 mmol), potassium acetate (2.3 g, 23 mmol) in 1,4-dioxane (20 ml), bis(pinacolate)diboron (3.5 g, 14 mmol) chromatography over silica gel, eluting with 6/1 (v/v) hexane/ethyl acetate gave crude **4b** as slight brown oil. Yield 90%; ^{1}H-NMR (500 MHz, $CDCl_3$) δ 7.76 (d, *J* = 8.5 Hz, Ar-*H*, 2H), 6.90 (d, *J* = 8.5 Hz, Ar-*H*, 2H), 4.02-3.98 (m, -C*H*$_2$-, 2H), 3.66 (t, *J* = 6.3 Hz, -C*H*$_2$-, 2H), 1.85-1.80 (m, -C*H*$_2$-, 2H), 1.63-1.57 (m, -C*H*$_2$-, 2H), 1.55-1.51 (m, -C*H*$_2$-, 2H), 1.35 (s, -C*H*$_3$, 12H), 0.92 (s, -C*H*$_3$, 9H), 0.07 (s, -C*H*$_3$, 6H) ppm (Figure S46).

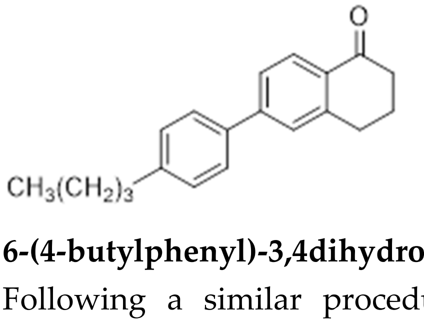

6-(4-butylphenyl)-3,4dihydronaphthalene-1(2*H*)-one (6b)

Following a similar procedure used for **6a** from **5** (3.4 g, 15 mmol), 4-butylphenylboronic acid (4.0 g, 19 mmol), potassium phosphate (9.7 g, 45 mmol) in solvent (30 ml/12 ml/6 ml; toluene/water/methanol), $Pd(PPh_3)_4$ (0.52 g, 0.45 mmol) chromatography over silica gel, eluting with 6/1 (v/v) hexane/ethyl acetate gave crude **6b** as brown solid. Yield 90%; ^{1}H-NMR (500 MHz, $CDCl_3$) δ 8.09 (d, *J* = 8.2 Hz, Ar-*H*, 1H), 7.55-7.52 (m, Ar-*H*, 3H), 7.46 (s, Ar-*H*, 1H), 7.27 (d, *J* = 8.2 Hz, Ar-*H*, 2H), 3.02 (t, *J* = 6.0 Hz, -C*H*$_2$-, 2H), 2.69-2.63 (m, -C*H*$_2$-, 4H), 2.20-2.15 (m, -

CH_2-, 2H), 1.67-1.61 (m, -CH_2-, 2H), 1.39-1.38 (m, -CH_2-, 2H), 0.95 (t, *J* = 7.3 Hz, -CH_3, 3H) ppm (Figure S48).

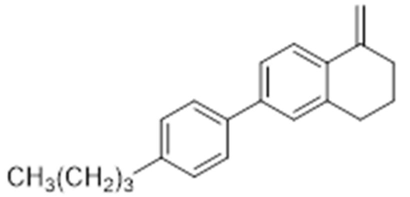

6-(4-butylphenyl)-1-methylene-1,2,3,4-tetrahydronaphthalene (7b)

Following a similar procedure used for **7a** from Methyltriphenylphosphonium bromide (7.1 g, 19 mmol), potassium *tert*-butoxide (2.4 g, 21mmol), THF (40ml), and **6b** (3.6 g, 14.5mmol), chromatography over silica gel, eluting with 6/1 (v/v) hexane/ethyl acetate gave crude **6b** as brown solid. Yield 90%; ^{1}H-NMR (500 MHz, $CDCl_3$) δ 7.71 (d, *J* = 8.2 Hz, Ar-*H*, 1H), 7.50 (d, *J* = 7.9 Hz, Ar-*H*, 2H), 7.40-7.37 (m, Ar-*H*, 1H), 7.33-7.32 (m, Ar-*H*, 1H), 7.24 (d, *J* = 8.2 Hz, Ar-*H*, 2H), 5.51 (s, C=C*H*, 1H), 4.96 (d, *J* = 0.9 Hz, C=C*H*, 1H), 2.90 (t, *J* = 6.3 Hz, -CH_2-, 2H), 2.64 (t, *J* = 7.8 Hz, -CH_2-, 2H), 2.57 (t, *J* = 6.1 Hz, -CH_2-, 2H), 1.94-1.89 (m, -CH_2-, 2H), 1.66-1.60 (m, -CH_2-, 2H), 1.39 (td, *J* = 14.9, -CH_2-, 7.5 Hz, 2H), 0.94 (t, *J* = 7.5 Hz, -CH_3, 3H) ppm (Figure S50).

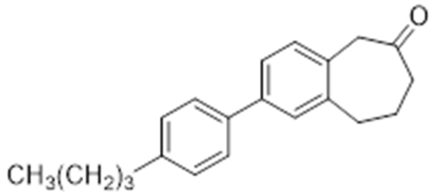

2-(4-butylphenyl)-5,7,8,9-tetrahydro-6*H*-benzo[7]annulen-6-one (8b)

Following a similar procedure used for **8a** from **7b** (3.6 g, 13 mmol), solvent (29 ml/1 ml; methanol/water), and HTIB ([hydroxy(tosyloxy)iodo]benzene; 5.6 g, 14 mmol) chromatography over silica gel, eluting with 6/1 (v/v) hexane/ethyl acetate gave crude **8b** as colorless solid. Yield 88%; ^{1}H-NMR (500 MHz, $CDCl_3$) δ 7.49 (d, *J* = 7.9 Hz, Ar-*H*, 2H), 7.41-7.38 (m, Ar-*H*, 2H), 7.25 (d, *J* = 8.2 Hz, Ar-*H*, 2H), 7.20 (d, *J* = 7.6 Hz, Ar-*H*, 1H), 3.76 (s, -CH_2-, 2H), 3.01 (t, *J* = 6.3 Hz, -CH_2-, 2H), 2.66-2.59 (m, -CH_2-, 4H), 2.06-2.01 (m, -CH_2-, 2H), 1.66-1.60 (m, -CH_2-, 2H), 1.42-1.36 (m, -CH_2-, 2H), 0.95 (t, *J* = 7.3 Hz, -CH_3, 3H) ppm (Figure S52).

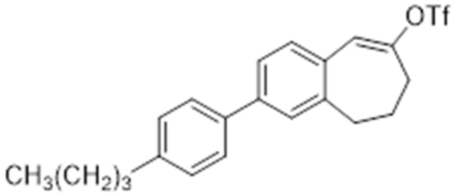

3-(4-butylphenyl)-6,7-dihydro-5*H*-benzo[7]annulen-8-yltrifluoromethanesulfonate (9b)

Following a similar procedure used for **9a** from **8b** (3.4 g, 12 mmol), THF (30 ml), *tert*-butoxide (1.7 g, 15 mmol), *N*-phenylbis(trifluolomethanesulfonimide) (5.3 g, 15 mmol) chromatography over silica gel, eluting with 6/1 (v/v) hexane/ethyl acetate gave crude **9b** as slight yellow solid. Yield >99%; ^{1}H-NMR (500 MHz, $CDCl_3$) δ 7.50 (d, *J* = 8.2 Hz, Ar-*H*, 2H), 7.43-7.40 (m, Ar-*H*, 1H), 7.34-7.33 (m, Ar-*H*, 1H), 7.25 (d, *J* = 7.9 Hz, Ar-*H*, 2H), 7.22 (d, *J* = 7.9 Hz, Ar-*H*, 1H), 6.61 (s, 1H), 2.95 (t, *J* = 5.0 Hz, -CH_2-, 2H), 2.81 (t, *J* = 6.4 Hz, -CH_2-, 2H), 2.65 (t, *J* = 7.8 Hz, -CH_2-, 2H), 2.05-2.01 (m, -CH_2-, 2H), 1.66-1.60 (m, -CH_2-, 2H), 1.39-1.37 (m, -CH_2-, 2H), 0.94 (t, *J* = 7.3 Hz, -CH_3, 3H) ppm (Figure S54).

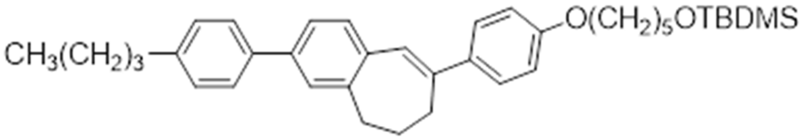

***tert*-butyl((5-(4-(3-(4-butylphenyl)-6,7-dihydro-5*H*-benzo[7]annulen-8-yl)phenoxy)pentyl)oxy)dimethylsilane (10b)**

Following a similar procedure used for **10a** from **9b** (3.4 g, 8.0 mmol), **4b** (3.9 g, 10 mmol), potassium phosphate (5.1 g, 24 mmol), THF (30 ml), and $Pd(PPh_3)_4$ (0.19 g, 0.16 mmol) chromatography over silica gel, eluting with 6/1 (v/v) hexane/ethyl acetate gave crude **9b** as a Colorless solid. Yield 98%; ^{1}H-NMR (500 MHz, $CDCl_3$) δ 7.57 (d, *J* = 7.9 Hz, Ar-*H*, 2H), 7.48-7.42 (m, Ar-*H*, 4H), 7.30-7.27 (m, Ar-*H*, 3H), 6.92 (d, *J* = 8.5 Hz, Ar-*H*, 2H), 6.80 (s, 1H), 4.02 (t, *J* = 6.4 Hz, -CH_2-, 2H), 3.68 (t, *J* = 6.4 Hz, -CH_2-, 2H), 2.90 (t, *J* = 6.0 Hz, -CH_2-, 2H), 2.72-2.67 (m, -CH_2-, 4H), 2.26 (t, *J* = 6.3 Hz, -CH_2-, 2H), 1.85 (t, *J* = 7.3 Hz, -CH_2-, 2H), 1.69-1.61 (m, -CH_2-, 4H), 1.57-1.54 (m, -CH_2-, 2H), 1.42 (q, *J* = 7.4 Hz, -CH_2-, 2H), 0.98 (t, *J* = 7.3 Hz, -CH_3, 3H), 0.94 (s, -CH_3, 9H), 0.09 (s, -CH_3, 6H) ppm (Figure S56).

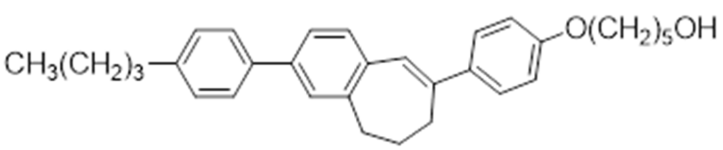

5-(4-(3-(4-butylphenyl)-6,7-dihydro-5*H*-benzo[7]annulen-8-yl)phenoxy)pentan-1-ol (11b)

Following a similar procedure used for **11a** from **10b** (4.4 g, 7.8 mmol), THF (4 ml), and 12M HCl aq (4 ml). Purification by recrystallization ($CHCl_3$/Hex) gave title compound **11b** as colorless solid. Yield 84%; ^{1}H-NMR (500 MHz, $CDCl_3$) δ 7.54 (d, *J* = 7.9 Hz, Ar-*H*, 2H), 7.46-7.39 (m, Ar-*H*, 4H), 7.26-7.24 (m, Ar-*H*, 3H), 6.89 (d, *J* = 8.5 Hz, Ar-*H*, 2H), 6.77 (s, 1H), 4.00 (t, *J* = 6.4 Hz, -CH_2-, 2H), 3.70 (t, *J* = 6.4 Hz, -CH_2-, 2H), 2.87 (t, *J* = 6.1 Hz, -CH_2-, 2H), 2.69-2.63 (m, -CH_2-, 4H), 2.23 (t, *J* = 6.3 Hz, -CH_2-, 2H), 1.84 (t, *J* = 7.5 Hz, -CH_2-, 2H), 1.68-1.62 (m, -CH_2-, 4H), 1.59-1.56 (m, -CH_2-, 2H), 1.39 (q, *J* = 7.4 Hz, -CH_2-, 2H), 0.95 (t, *J* = 7.3 Hz, -CH_3, 3H) ppm (Figure S58).

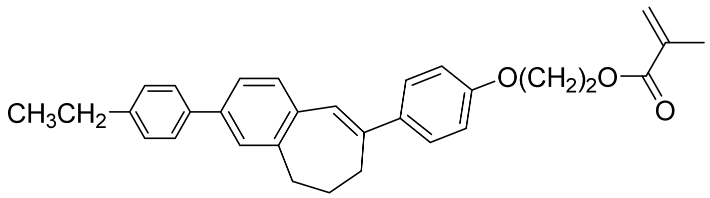

2-(4-(3-(4-ethylphenyl)-6,7-dihydro-5*H*-benzo[7]annulen-8-yl)phenoxy)ethyl methacrylate (M2)

Following a similar procedure used for **M1** from **11a** (0.71 g, 1.8 mmol), triethylamine (0.38 ml, 2.7 mmol), dichloromethane (10 ml), and methacryloyl chloride (0.23ml, 2.4 mmol) chromatography over silica gel, eluting with 1/1 (v/v) hexane/ethyl acetate gave crude **M2** as colorless solid. Purification by recrystallization (hexane/dichloromethane) gave a colorless solid. Yield 65%; ^{1}H-NMR (500 MHz, $CDCl_3$) δ 7.55 (d, *J* = 8.2 Hz, Ar-*H*, 2H), 7.46 (d, *J* = 8.5 Hz, Ar-*H*, 2H), 7.42 (dd, *J* = 7.8, 1.7 Hz, Ar-*H*, 1H), 7.39 (s, Ar-*H*, 1H), 7.28-7.25 (m, Ar-*H*, 3H), 6.93 (d, *J* = 8.9 Hz, Ar-*H*, 2H), 6.77 (s, 1H), 6.16 (s, =C*H*, 1H), 5.60 (t, *J* = 1.5 Hz, =C*H*, 1H), 4.52 (t, *J* = 4.9 Hz, -CH_2-, 2H), 4.26 (t, *J* = 4.9 Hz, -CH_2-, 2H), 2.88 (t, *J* = 6.1 Hz, -CH_2-, 2H), 2.72-2.66 (m, -CH_2-, 4H), 2.26-2.20 (m, -CH_2-, 2H), 1.88-2.02 (s, -CH_3, 3H), 1.28 (t, *J* = 7.6 Hz, -CH_3, 3H) ppm (Figure S61); ^{13}C-NMR (100 MHz, $CDCl_3$) δ 167.5, 158.0, 143.4, 142.4, 141.6, 139.2, 138.4, 137.5, 136.4, 136.1, 131.1, 128.4, 127.7, 127.5, 127.4, 127.0, 126.2, 124.6, 114.6, 66.2, 63.2, 35.0, 33.1, 30.3, 28.7, 18.5, 15.7 ppm (Figure S62). IR (KBr) 3050, 3014, 2964, 2926, 1717, 1602, 1542, 1509, 1489, 1457, 1439, 1401, 1321, 1273, 1251, 1157, 1118, 1073, 1048, 943, 921, 895, 888, 922, 804 cm^{-1}. (Figure S80). HRMS (EI) Calcd for $C_{31}H_{32}O_3$: 452.5933, Found 452.2351 (Figure S84).

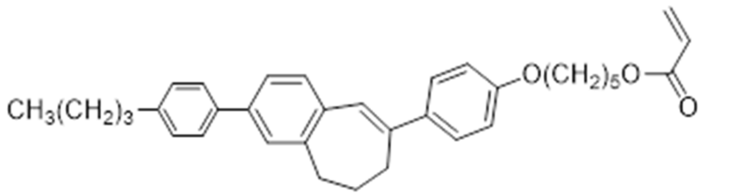

5-(4-(3-(4-butylphenyl)-6,7-dihydro-5*H*-benzo[7]annulen-8-yl)phenoxy)pentyl acrylate (M3)

Following a similar procedure used for **M1** from **11b** (0.91 g, 2.0 mmol), triethylamine (0.42 ml, 3.0 mmol), dichloromethane (5 ml), and acryloyl chloride (0.21 ml, 2.6 mmol) chromatography over silica gel, eluting with 1/1 (v/v) hexane/ethyl acetate gave crude **M3** as colorless solid. Purification by recrystallization (hexane/dichloromethane) gave a colorless solid. Yield 81%; ^{1}H-NMR (500 MHz, $CDCl_3$) δ 7.53 (d, *J* = 8.2 Hz, Ar-*H*, 2H), 7.45-7.39 (m, Ar-*H*, 4H), 7.26-7.24 (m, Ar-*H*, 3H), 6.89 (d, *J* = 8.5 Hz, Ar-*H*, 2H), 6.77 (s, 1H), 6.41 (d, *J* = 17.4 Hz, =C*H*, 1H), 6.13 (dd, *J* = 17.4, 10.4 Hz, -C*H*=, 1H), 5.82 (d, *J* = 10.7 Hz, =C*H*, 1H), 4.20 (t, *J* = 6.6 Hz, -CH_2-, 2H), 4.00 (t, *J* = 6.4 Hz, -CH_2-, 2H), 2.87 (t, *J* = 6.0 Hz, -CH_2-, 2H), 2.69-2.63 (m, -CH_2-, 4H), 2.23 (q, *J* = 6.3 Hz, -CH_2-, 2H), 1.87-1.82 (m, -CH_2-, 2H), 1.80-1.74 (m, -CH_2-, 2H), 1.67-1.57 (m, -CH_2-, 4H), 1.39 (q, *J* = 7.4 Hz, -CH_2-, 2H), 0.95 (t, *J* = 7.3 Hz, -CH_3, 3H) ppm (Figure S63); ^{13}C-NMR (100 MHz, $CDCl_3$) δ 166.4, 158.5, 142.4, 142.0, 141.6, 139.1, 138.3, 136.8, 136.5, 131.1, 130.7, 128.9, 128.7, 127.7, 127.4, 127.1, 126.9, 124.5, 114.4, 67.8, 64.6, 35.4, 35.0, 33.8, 33.1, 30.4, 29.1, 28.5, 22.8, 22.5, 14.1 ppm (Figure S64). HRMS (EI) Calcd for $C_{35}H_{40}O_3$: 508.7008, Found 508.2977 (Figure S85).

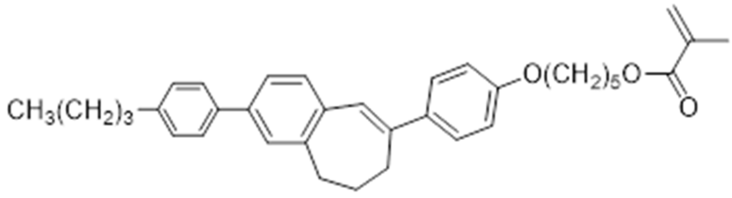

5-(4-(3-(4-butylphenyl)-6,7-dihydro-5*H*-benzo[7]annulen-8-yl)phenoxy)pentyl methacrylate (M4)

Following a similar procedure used for **M1** from **11b** (1.0 g, 2.2 mmol), triethylamine (0.45 ml, 2.9 mmol), dichloromethane (10 ml), and methacryloyl chloride (0.27 ml, 2.9 mmol) chromatography over silica gel, eluting with 1/1 (v/v) hexane/ethyl acetate gave crude **M4** as colorless solid. Purification by

recrystallization (hexane/dichloromethane) gave a colorless solid. Yield 86%; ^{1}H-NMR (500 MHz, $CDCl_3$) δ 7.54 (d, *J* = 8.2 Hz, Ar-*H*, 2H), 7.46-7.39 (m, Ar-*H*, 4H), 7.27-7.24 (m, Ar-*H*, 3H), 6.89 (d, *J* = 8.9 Hz, Ar-*H*, 2H), 6.77 (s, 1H), 6.11 (s, =C*H*, 1H), 5.56 (t, *J* = 1.5 Hz, =C*H*, 1H), 4.19 (t, *J* = 6.7 Hz, -CH_2-, 2H), 4.00 (t, *J* = 6.4 Hz, -CH_2-, 2H), 2.87 (t, *J* = 6.0 Hz, -CH_2-, 2H), 2.69-2.63 (m, -CH_2-, 4H), 2.25-2.20 (m, -CH_2-, 2H), 1.95 (s, -CH_3, 3H), 1.88-1.82 (m, -CH_2-, 2H), 1.80-1.74 (m, -CH_2-, 2H), 1.68-1.57 (m, -CH_2-, 4H), 1.39 (td, *J* = 14.9, 7.5 Hz, -CH_2-, 2H), 0.95 (t, *J* = 7.3 Hz, -CH_3, 3H) ppm (Figure S64); ^{13}C-NMR (100 MHz, $CDCl_3$) δ 167.6, 158.5, 142.4, 142.0, 141.6, 139.1, 138.3, 136.8, 136.6, 136.5, 131.1, 128.9, 127.7, 127.4, 127.1, 126.9, 125.4, 124.5, 114.4, 67.8, 64.7, 35.4, 34.9, 33.8, 33.1, 30.4, 29.1, 28.5, 22.8, 22.5, 18.5, 14.1 ppm (Figure S66). IR (KBr) 3019, 2954, 2928, 2869, 1715, 1638, 1602, 1543, 1509, 1451, 1434, 1397, 1331,1254, 1183, 1121, 1014, 976, 937, 902, 830, 648, 549, 515 cm^{-1}. (Figure S80) HRMS (EI) Calcd for $C_{36}H_{42}O_3$: 522.7277, Found 522.3134 (Figure S86).

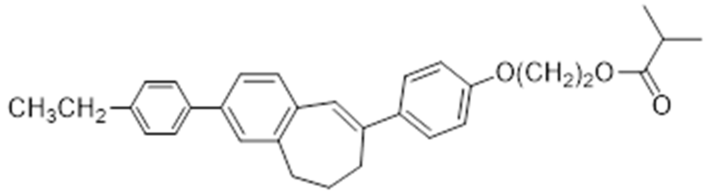

2-(4-(3-(4-ethylphenyl)-6,7-dihydro-5*H*-benzo[7]annulen-8-yl)phenoxy)ethyl isobutyrate (M5)

Following a similar procedure used for **M1** from **11a** (0.19 g, 0.5 mmol), triethylamine (0.10 ml, 0.72 mmol), dichloromethane (2.0 ml), and isobutyryl chloride (0.060ml, 0.57 mmol) chromatography over silica gel, eluting with 1/1 (v/v) hexane/ethyl acetate gave crude **M5** as colorless solid. Purification by recrystallization (hexane/dichloromethane) gave a Colorless solid. Yield 89%; ^{1}H-NMR (500 MHz, $CDCl_3$) δ 7.55 (d, *J* = 7.9 Hz, Ar-*H*, 2H), 7.46 (d, *J* = 8.5 Hz, Ar-*H*, 2H), 7.42 (dd, *J* = 7.9, 1.8 Hz, Ar-*H*, 1H), 7.39 (s, Ar-*H*, 1H), 7.28-7.25 (m, Ar-*H*, 3H), 6.92 (d, *J* = 8.9 Hz, Ar-*H*, 2H), 6.77 (s, 1H), 4.44 (t, *J* = 4.7 Hz, -CH_2-, 2H), 4.21 (t, *J* = 4.9 Hz, -CH_2-, 2H), 2.88 (t, *J* = 6.1 Hz, -CH_2-, 2H), 2.72-2.66 (m, -CH_2-, 4H), 2.64-2.58 (m, -C(C)*H*-, 1H), 2.23 (t, *J* = 6.1 Hz, -CH_2-, 2H), 1.55 (s, -CH_2-, 4H), 1.28 (t, *J* = 7.6 Hz, -CH_3, 3H), 1.19 (d, *J* = 7.0 Hz, -CH_3, 6H) ppm (Figure S67); ^{13}C-NMR (100 MHz, $CDCl_3$) δ 177.3, 158.0, 143.4, 142.3, 141.6, 139.2, 138.4, 137.4, 136.4, 131.1, 128.4, 127.7, 127.5, 127.4, 127.0, 124.5, 114.6, 77.5, 77.2, 76.8, 66.3, 62.8, 35.0, 34.0, 33.1, 30.3, 28.7, 19.1, 15.7 ppm (Figure S68). HRMS (EI) Calcd for $C_{31}H_{34}O_3$: 454.6092, Found 454.2508 (Figure S87).

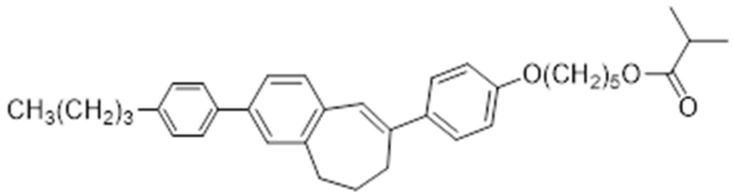

5-(4-(3-(4-butylphenyl)-6,7-dihydro-5*H*-benzo[7]annulen-8-yl)phenoxy)pentyl isobutyrate (M6)

Following a similar procedure used for **M1** from **11b** (0.23 g, 0.50 mmol), triethylamine (0.10 ml, 0.72 mmol), dichloromethane (2.0 ml), and isobutyryl chloride (0.060ml, 0.57 mmol) chromatography over silica gel, eluting with 1/1 (v/v) hexane/ethyl acetate gave crude **M6** as colorless solid. Purification by recrystallization (hexane/dichloromethane) gave a Colorless solid. Yield 74%; ^{1}H-NMR (500 MHz, $CDCl_3$) δ 7.54 (d, *J* = 7.9 Hz, Ar-*H*, 2H), 7.46-7.39 (m, Ar-*H*, 4H), 7.27-7.24 (m, Ar-*H*, 3H), 6.89 (d, *J* = 8.5 Hz, Ar-*H*, 2H), 6.77 (s, 1H), 4.11 (t, *J* = 6.6 Hz, -CH_2-, 2H), 4.00 (t, *J* = 6.3 Hz, -CH_2-, 2H), 2.87 (t, *J* = 6.1 Hz, -CH_2-, 2H), 2.69-2.63 (m, -CH_2-, 4H), 2.59-2.51 (m, -C(C)*H*-, 1H), 2.23 (q, *J* = 6.3 Hz, -CH_2-, 2H), 1.87-1.81 (m, -CH_2-, 2H), 1.75-1.69 (m, -CH_2-, 2H), 1.67-1.61 (m, -CH_2-, 2H), 1.59-1.55 (m, -CH_2-, 2H), 1.39 (q, *J* = 7.4 Hz, -CH_2-, 2H), 1.17 (d, *J* = 7.0 Hz, -CH_3, 6H), 0.95 (t, *J* = 7.5 Hz, -CH_3, 3H) ppm (Figure S69); ^{13}C-NMR (100 MHz, $CDCl_3$) δ 177.4, 158.5, 142.5, 142.1, 141.6, 139.1, 138.4, 136.8, 136.5, 131.0, 128.9, 127.7, 127.4, 127.1, 126.9, 124.5, 114.4, 67.8, 64.3, 35.4, 35.0, 34.2, 33.8, 33.1, 30.4, 29.1, 28.6, 22.7, 22.6, 19.2, 14.1 ppm (Figure S70). HRMS (EI) Calcd for $C_{36}H_{44}O_3$: 524.7436, Found 524.3290 (Figure S88).

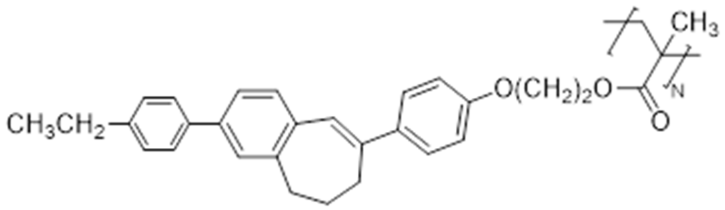

Poly[2-(4-(3-(4-ethylphenyl)-6,7-dihydro-5*H*-benzo[7]annulen-8-yl)phenoxy)ethyl] methacrylate (P2)

Following a similar procedure used for **P1** from **M2** gave a Colorless solid. Yield 34%; ^{1}H-NMR (500 MHz, $CDCl_3$) δ 7.46-7.04 (brm, Ar-*H*, 9H), 6.77-6.65 (brm, Ar-*H*, 3H), 4.05 (br, 4H), 2.68-2.48 (brm, 6H), 2.00 (br, 3H), 1.19-0.96 (brm, 6H) ppm (Figure S73); ^{13}C-NMR (100 MHz, $CDCl_3$) δ 177.9, 177.5, 176.8, 176.7, 157.8, 143.2,

141.9, 141.6, 138.9, 138.2, 137.0, 136.1, 131.4, 128.3, 127.5, 127.3, 127.2, 126.9, 126.6, 124.4, 114.5, 65.6, 65.4, 63.4, 54.7, 45.3, 45.0, 35.0, 33.2, 29.8, 28.6, 18.8, 16.9, 16.8, 15.7 ppm (Figure S74). IR (KBr) 3019, 2960, 2927, 2870, 1730, 1604, 1509, 1455, 1396, 1244, 1149, 1066, 924, 896, 827, 620, 529 cm^{-1} (Figure S81).

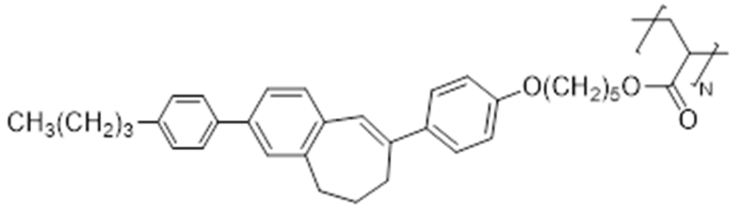

Poly[5-(4-(3-(4-butylphenyl)-6,7-dihydro-5*H*-benzo[7]annulen-8-yl)phenoxy)pentyl] acrylate (P3)

Following a similar procedure used for **P1** from **M3** gave a Colorless solid. Yield 19%; ^{1}H-NMR (500 MHz, $CDCl_3$) δ 7.52-7.23 (brm, Ar-*H*, 9H), 6.86-6.75 (brm, Ar-*H*, 3H), 4.04 (brm, 4H), 2.84 (br, 2H), 2.65 (br, 4H), 2.41-2.18 (brm, 3H), 1.82-1.64 (brm, 10H), 1.41 (br, 2H), 0.97 (br, 3H) ppm (Figure S75); ^{13}C-NMR (100 MHz, $CDCl_3$) δ 158.4, 142.3, 141.9, 141.5, 139.0, 138.3, 136.7, 136.4, 131.1, 128.9, 127.6, 127.3, 127.0, 126.8, 124.4, 114.3, 67.7, 64.6, 35.4, 34.9, 33.7, 33.1, 30.2, 29.1, 28.5, 22.7, 22.5, 14.1 ppm (Figure S76).

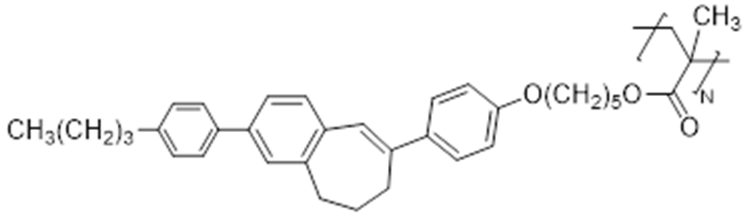

Poly[5-(4-(3-(4-butylphenyl)-6,7-dihydro-5*H*-benzo[7]annulen-8-yl)phenoxy)pentyl] methacrylate (P4)

Following a similar procedure used for **P1** from **M4** gave a Colorless solid. Yield 10%; ^{1}H-NMR (500 MHz, $CDCl_3$) δ 7.46 (br, 2H), 7.36-7.32 (brm, 4H), 7.17 (br, 3H), 6.81 (br, 2H), 6.72 (br, 1H), 3.95 (br, 4H), 2.77 (br, 2H), 2.61-2.57 (brm, 4H), 2.10 (br, 2H), 1.78-1.52 (brm, 11H), 1.37 (br, 2H), 1.12-0.93 (brm, 6H) ppm (Figure S77); ^{13}C-NMR (100 MHz, $CDCl_3$) δ 177.5, 176.9, 158.4, 142.2, 141.9, 141.5, 138.9, 138.2, 136.6, 136.3, 131.1, 128.8, 127.5, 127.3, 127.0, 126.8, 124.4, 114.3, 67.7, 64.9, 45.3, 45.0, 44.9, 35.4, 34.9, 33.7, 33.1, 30.1, 29.1, 28.1, 22.8, 22.5, 14.1 ppm (Figure S78). IR (KBr) 3019, 2951, 2928, 2857, 1726, 1604, 1509, 1498, 1474, 1396, 1246, 1179, 1154, 1179, 1073, 1026, 896, 828, 458, 433, 404 cm^{-1} (Figure S82).

2. *(Thermal gravimetric analysis) TGA charts*

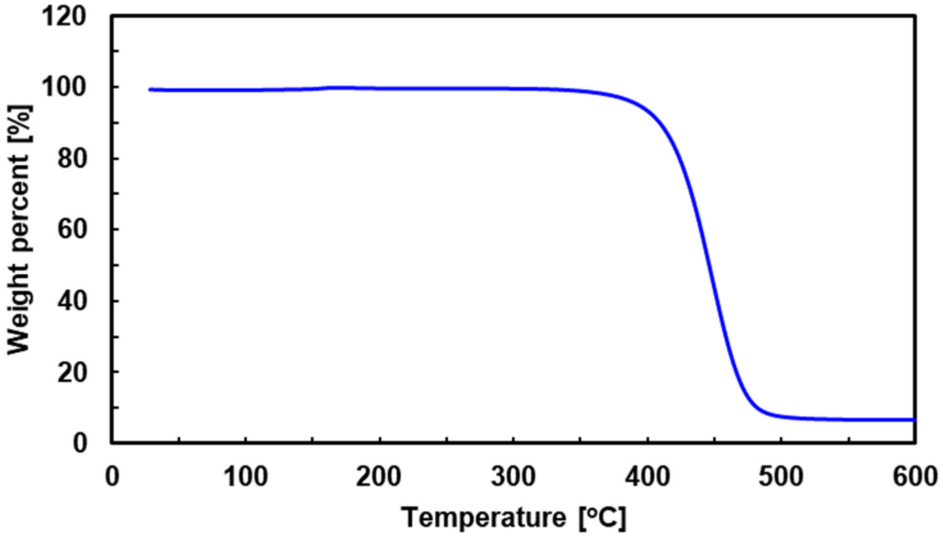

Figure S1. TGA curve for **P1** under nitrogen atmosphere at a heating rate of 20 °C min^{-1}.

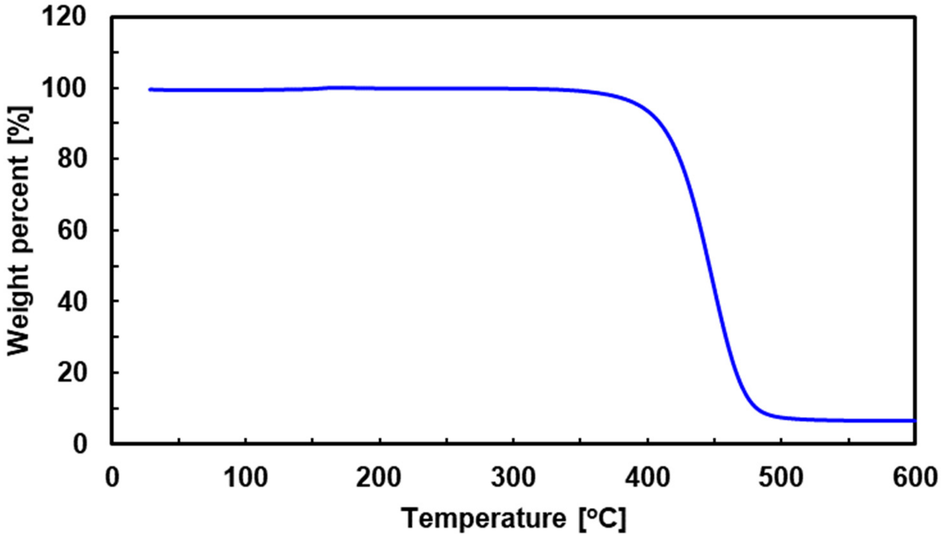

Figure S2. TGA curve for **P2** under nitrogen atmosphere at a heating rate of 20 °C min^{-1}.

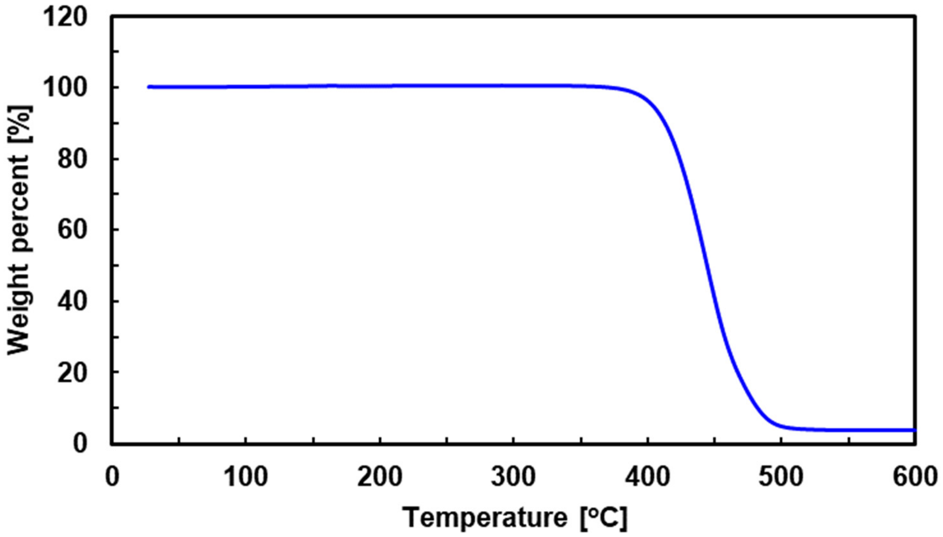

Figure S3. TGA curve for **P3** under nitrogen atmosphere at a heating rate of 20 ^{o}C min^{-1}.

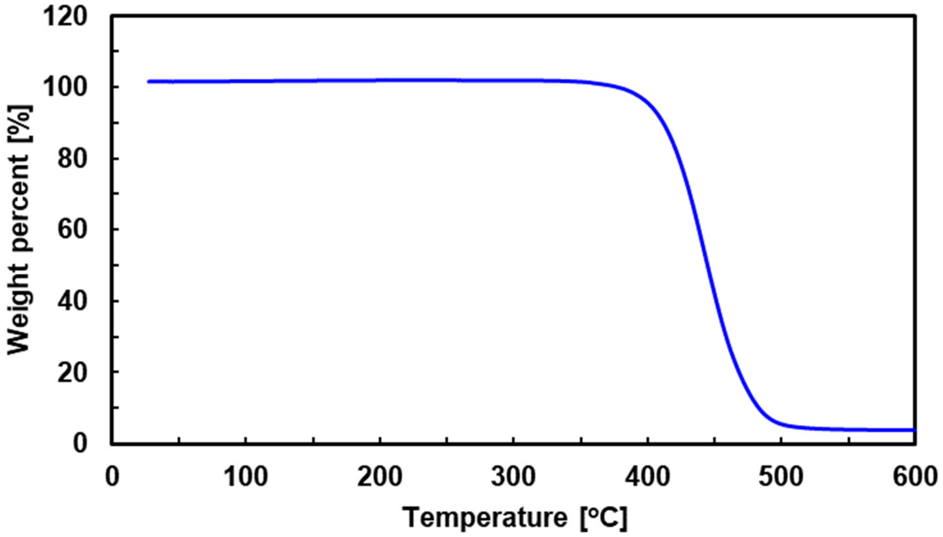

Figure S4. TGA curve for **P4** under nitrogen atmosphere at a heating rate of 20 ^{o}C min^{-1}.

3. Liquid crystal behavior
(differential scanning calorimetry) DSC thermogram.

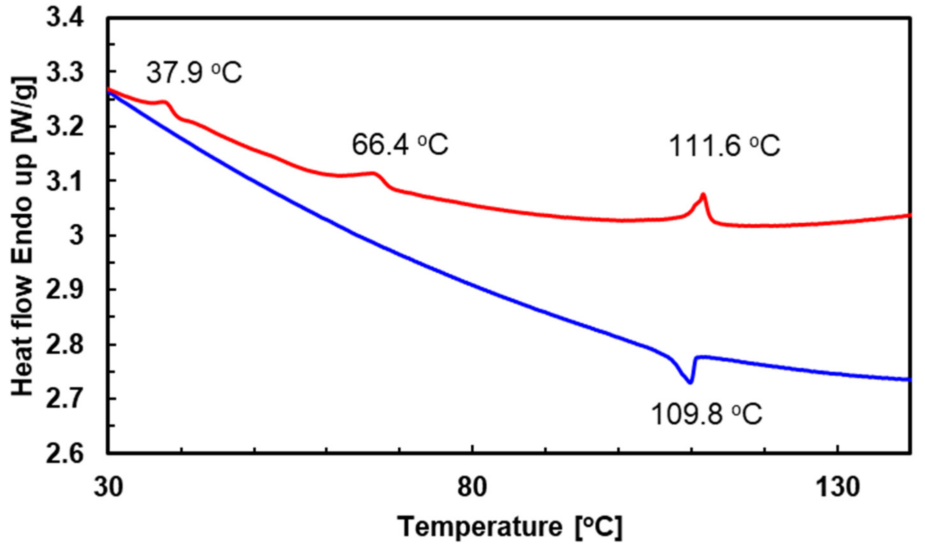

Figure S5. DSC thermogram of **M5** at a rate of 10 °C min^{-1} (2nd process).

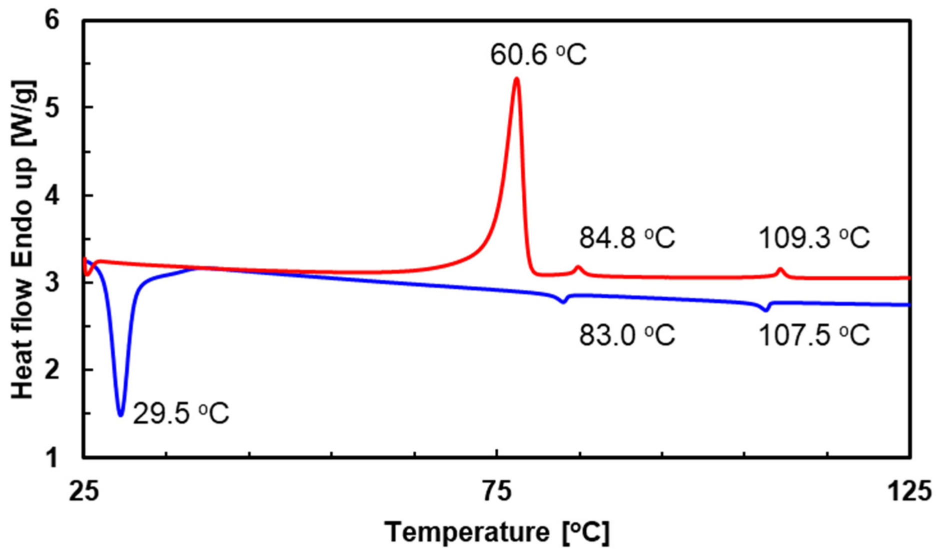

Figure S6. DSC thermogram of **M6** at a rate of 10 °C min^{-1} (2nd process).

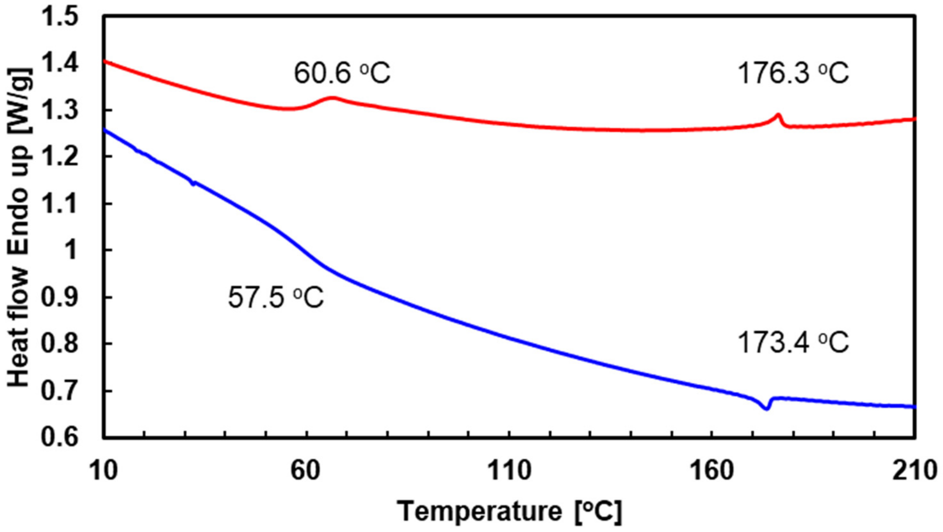

Figure S7. DSC thermogram of **P1** at a rate of 10 °C min^{-1} (3rd process).

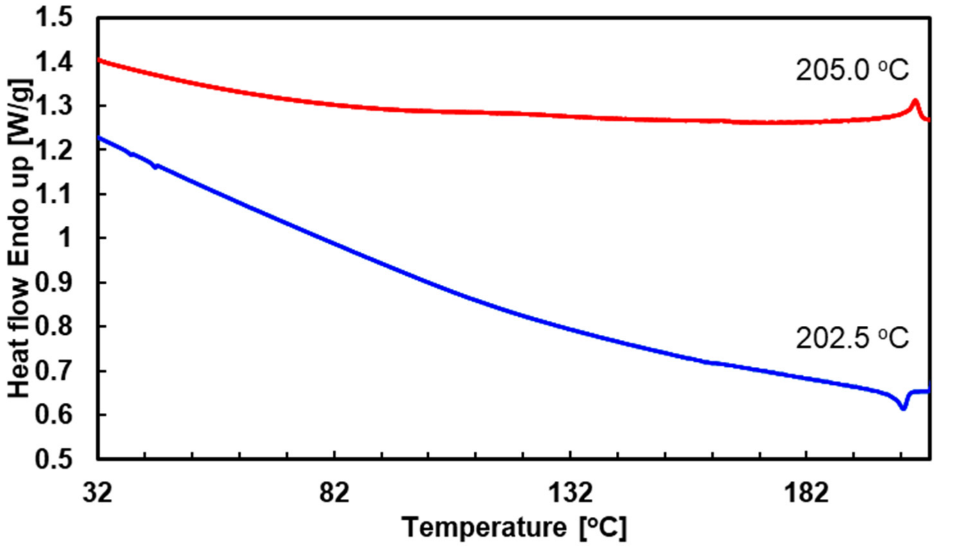

Figure S8. DSC thermogram of **P2** at a rate of 10 °C min^{-1} (3rd process).

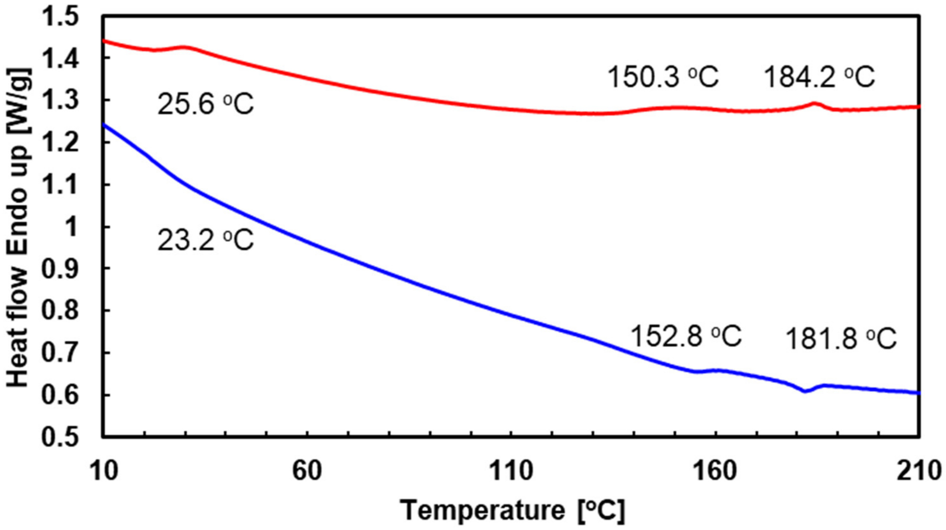

Figure S9. DSC thermogram of **P3** at a rate of 10 °C min^{-1} (3rd process).

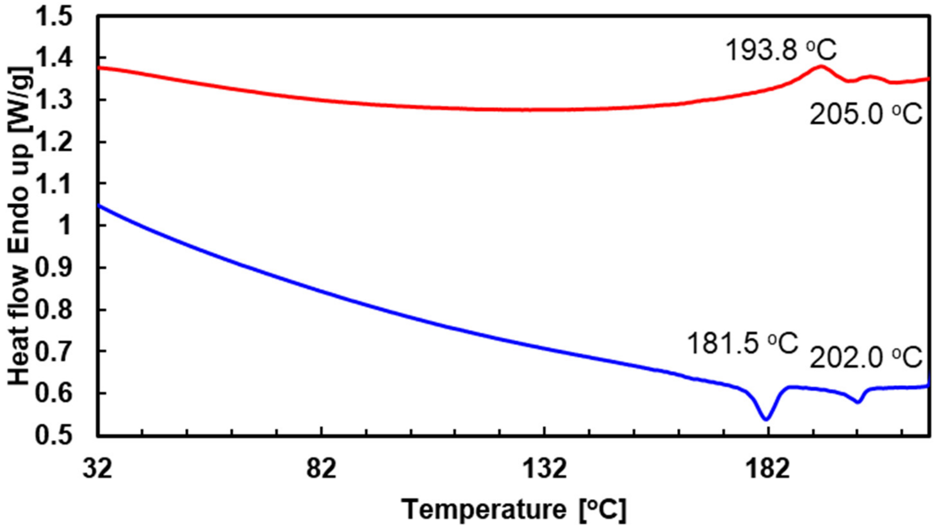

Figure S10. DSC thermogram of **P4** at a rate of 10 °C min^{-1} (3rd process).

(Polarized optical microscopy) POM images

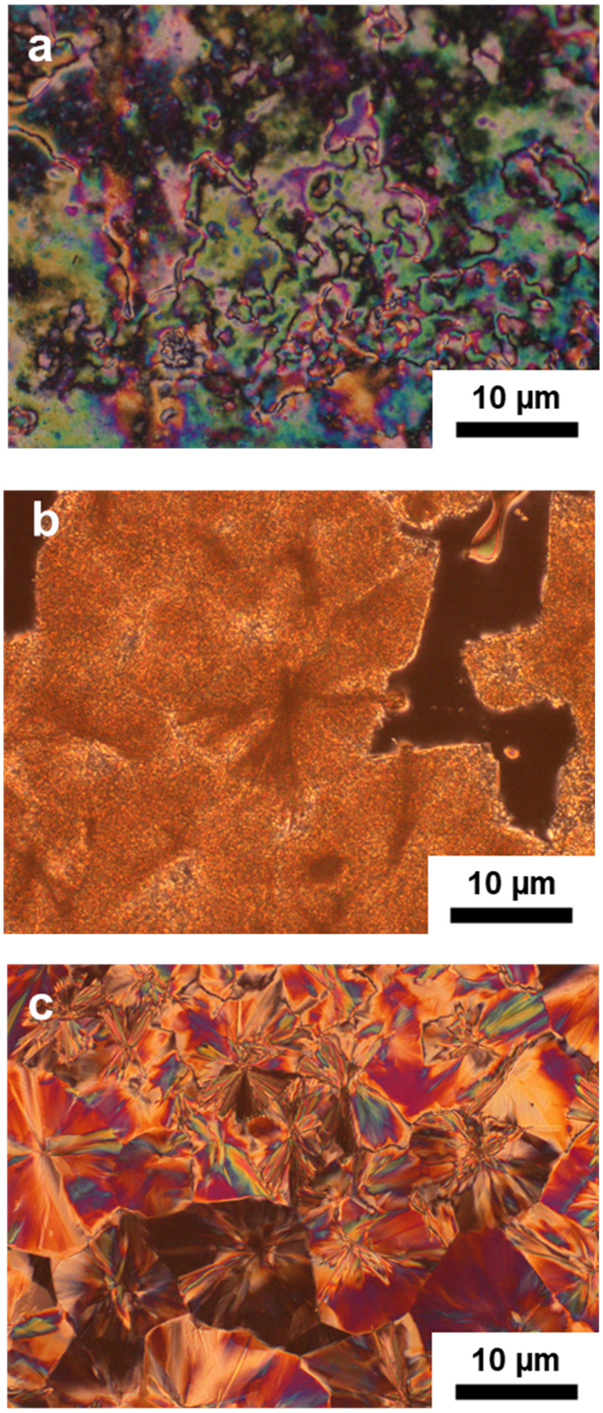

Figure S11. POM images of **M5** (a) at 108 °C in nematic (N) phase, (b) at 40 °C in crystal (Cr) phase, and (c) at 25 °C in crystal (Cr2) phase (bottom) on cooling.

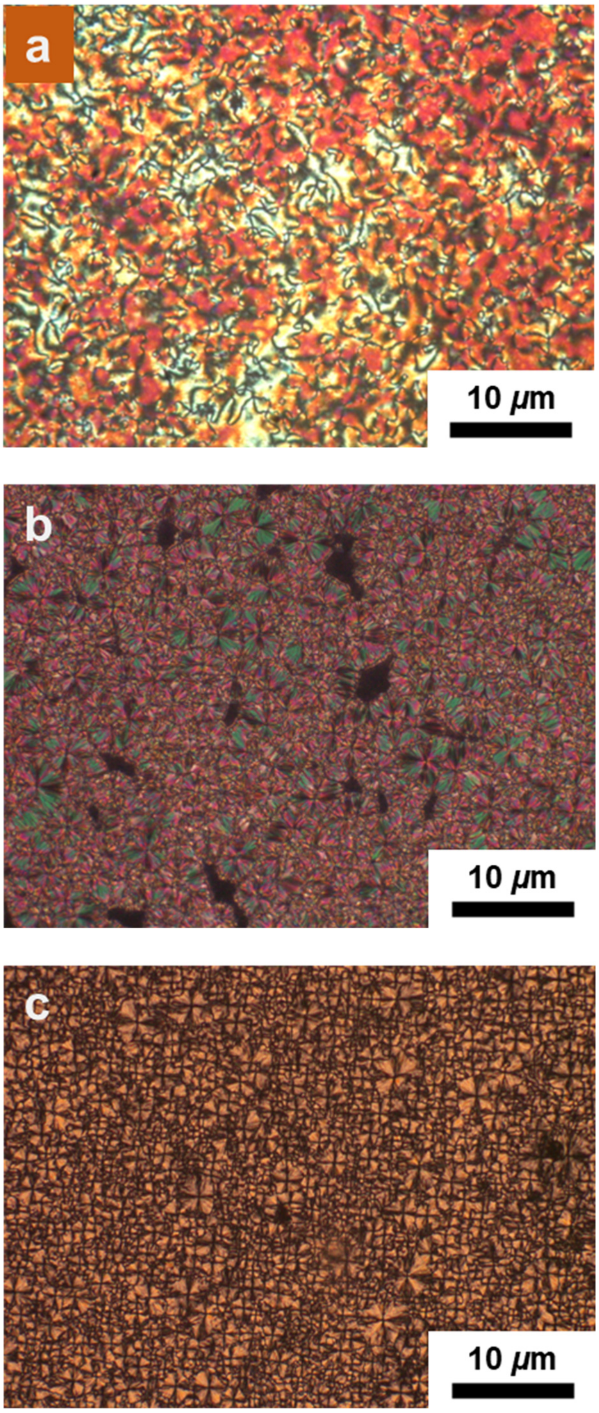

Figure S12. POM images of **M6** (a) at 106 °C in N phase, (b) at 81°C in smectic A (SmA) phase, and (c) at 30 °C in Cr phase on heating.

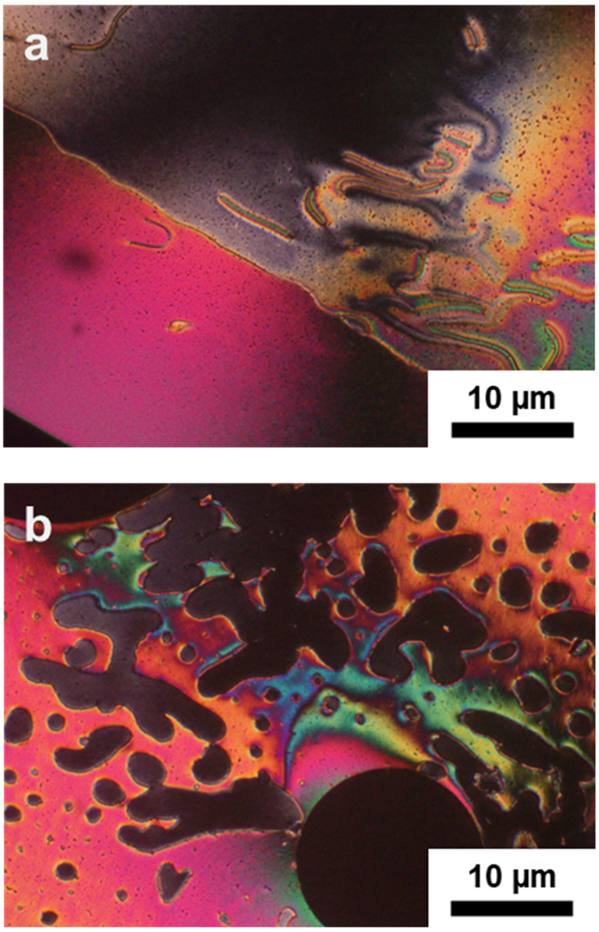

Figure S13. POM images of **P1** (a) at 159 °C in N phase on cooling, and (b) at 25 °C in glass N phase left at room temperature for 3 months.

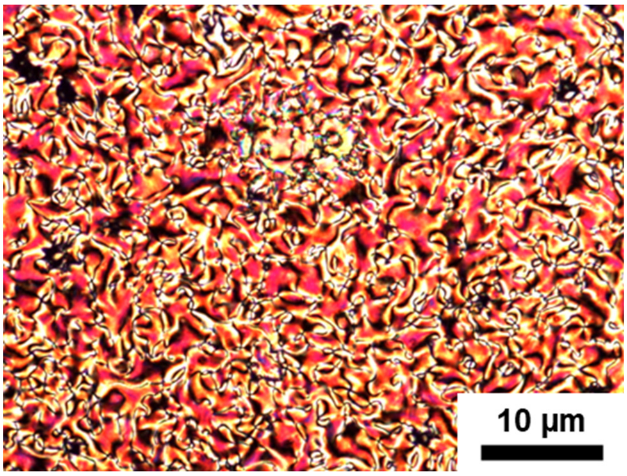

Figure S14. A POM image of **P2** at 25 °C in N phase left at room temperature for 3 months.

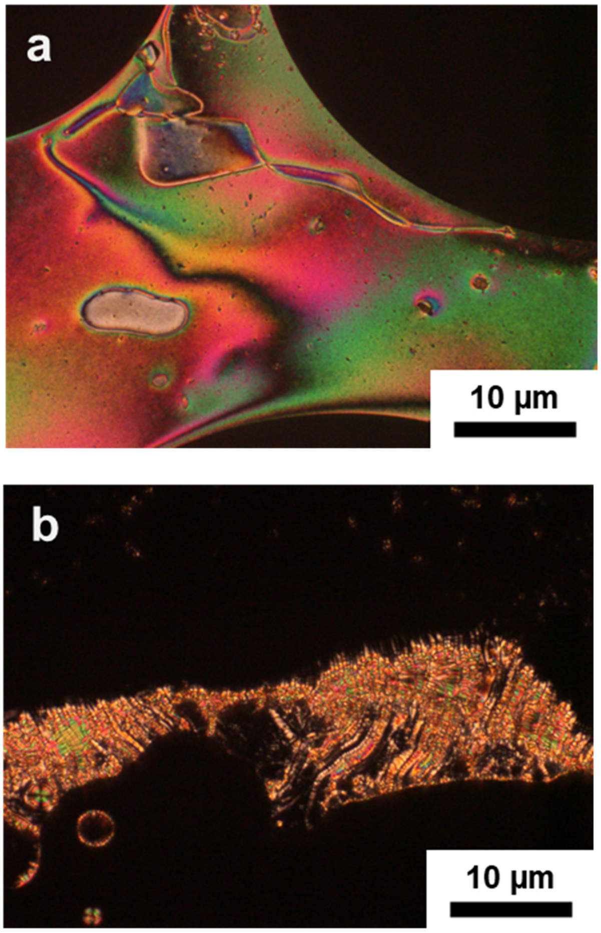

Figure S15. POM images of **P3** (a) at 160 °C in N phase and (a) at 50 °C in SmA phase on cooling.

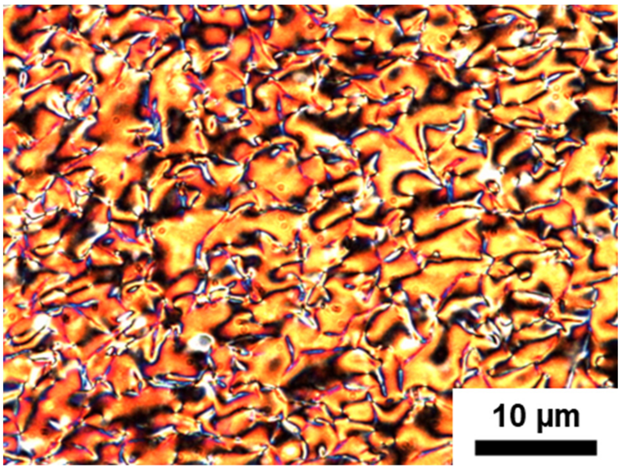

Figure S16. POM images of **P4** at 186 °C in N phase on cooling.

(wide angle x-ray diffraction) WAXD measurement

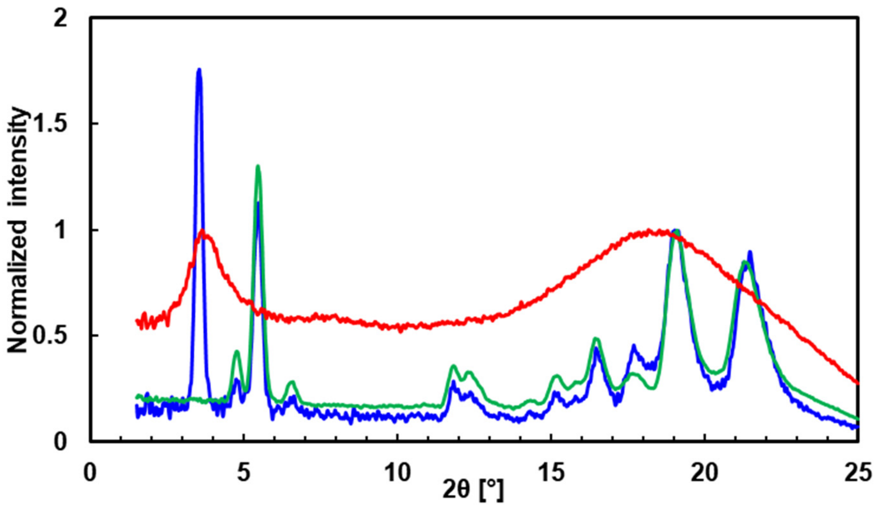

Figure S17. WAXD intensity profiles of **M5** at 30 °C (blue line), 50 °C (green line), and 80 °C (red line) on heating.

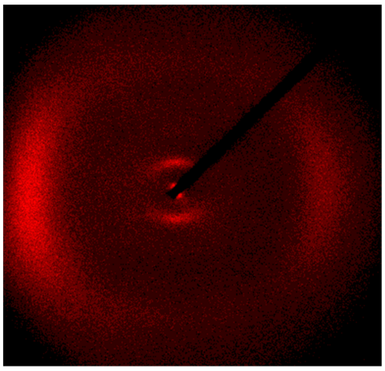

Figure S18. 2D-WAXD pattern for **M5** at 80 °C on cooling.

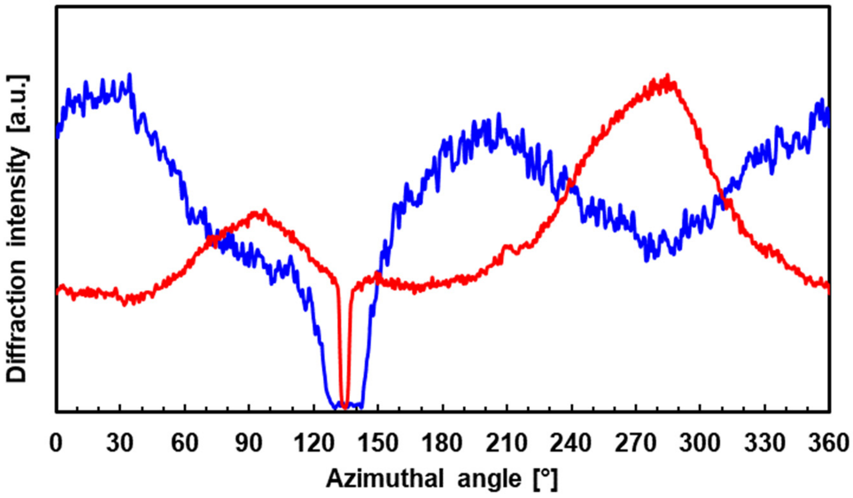

Figure S19. Azimuthal profiles of the diffraction for **M5** in the small-angle region (blue line) and wide-angle region (red line) at 80 °C on cooling.

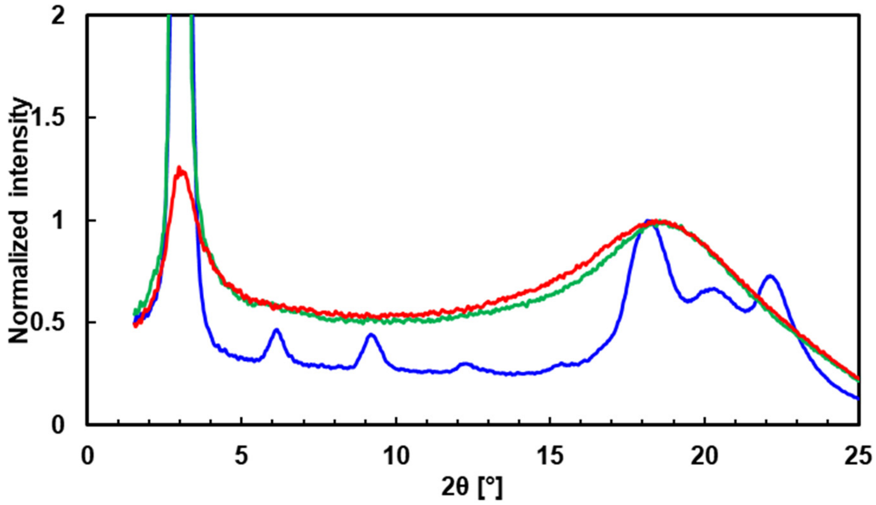

Figure S20. WAXD intensity profiles of **M6** at 30 °C (blue line), 80 °C (green line), and 100 °C (red line) on cooling.

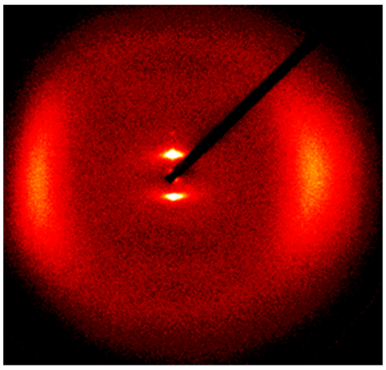

Figure S21. 2D-WAXD pattern for **M6** at 80 °C on cooling.

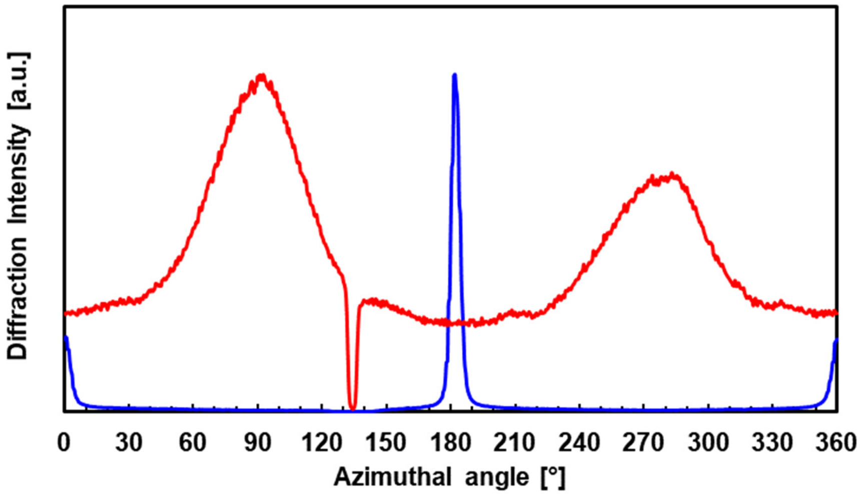

Figure S22. Azimuthal profiles of the diffraction for **M6** in the small-angle region (blue line) and wide-angle region (red line) at 80 °C on cooling.

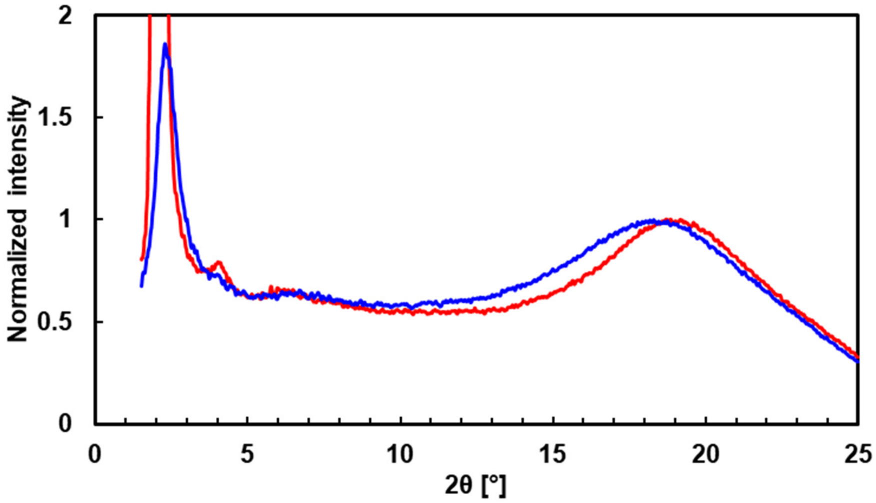

Figure S23. WAXD intensity profiles of **P1** at 40 °C (blue line) and 150 °C (red line) on cooling.

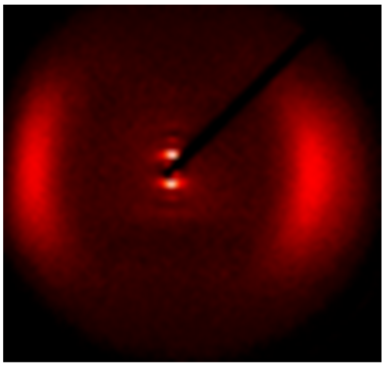

Figure S24. 2D-WAXD pattern for **P1** at 40 °C on cooling.

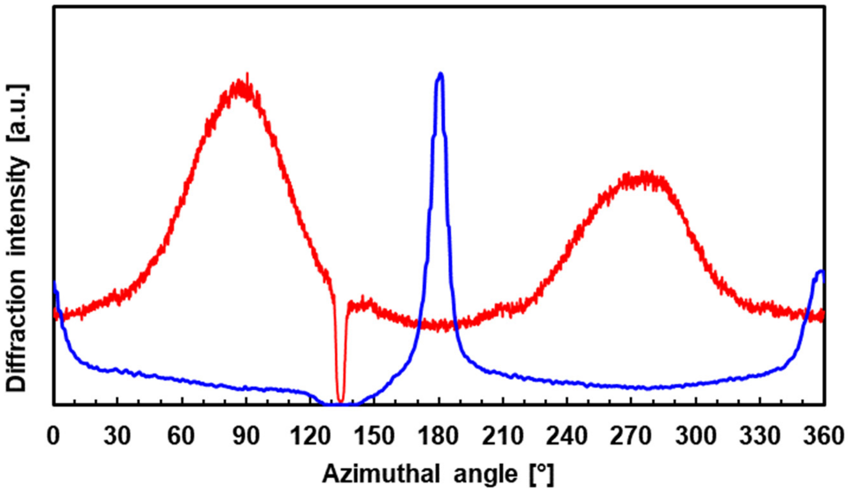

Figure S25. Azimuthal profiles of the diffraction for **P1** in the small-angle region (blue line) and wide-angle region (red line) at 40 °C on cooling.

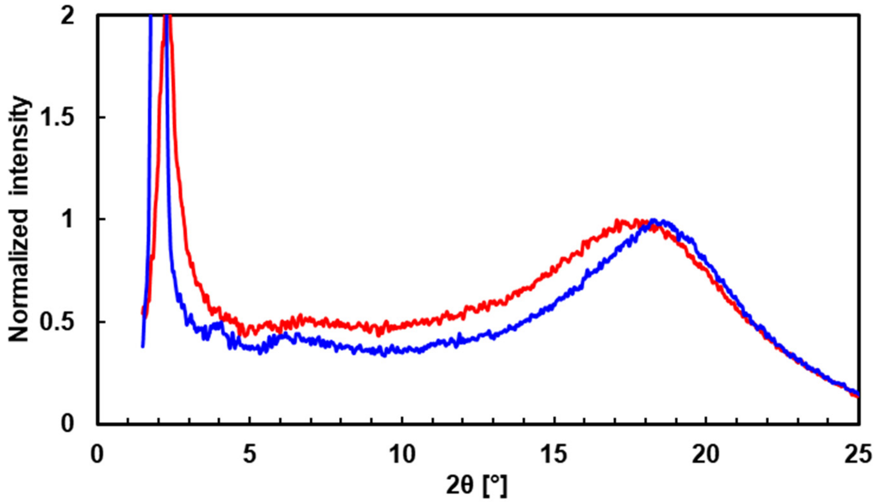

Figure S26. WAXD intensity profiles of **P2** at 30 °C (blue line) and 180 °C (red line) on cooling.

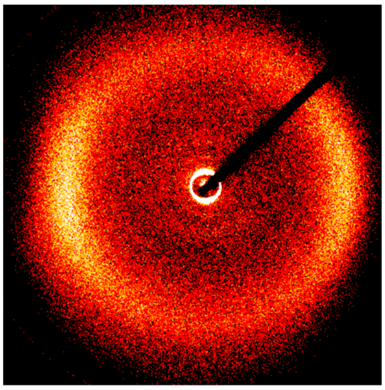

Figure S27. 2D-WAXD patterns for **P2** at 30 °C on cooling.

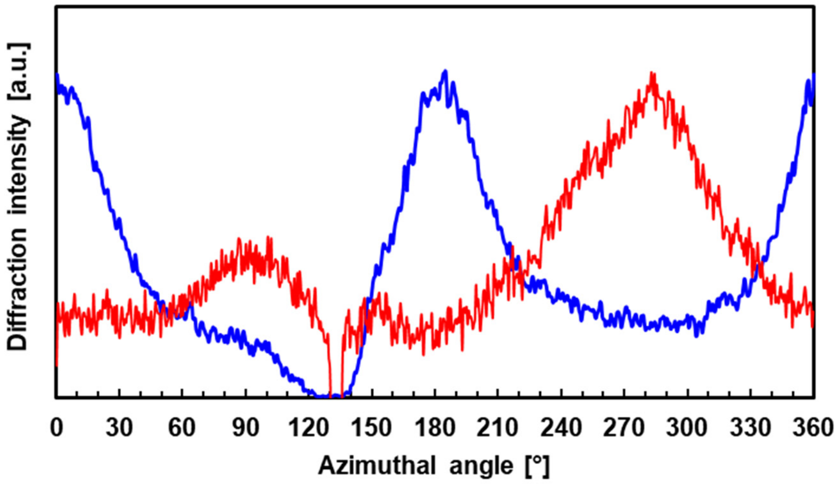

Figure 28. Azimuthal profiles of the diffraction for **P2** in the small-angle region (blue line) and wide-angle region (red line) at 30 °C on cooling.

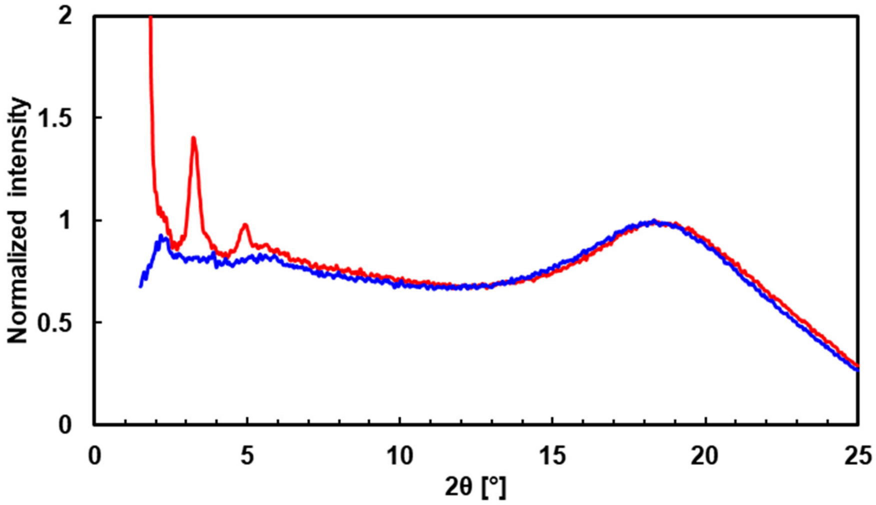

Figure S29. WAXD intensity profiles of **P3** at 140 °C (blue line) and 170 °C (red line) on cooling.

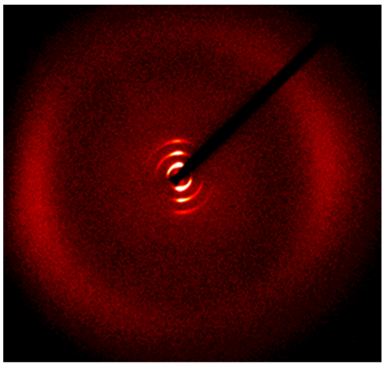

Figure S30. 2D-WAXD pattern for **P3** at 30 °C on cooling.

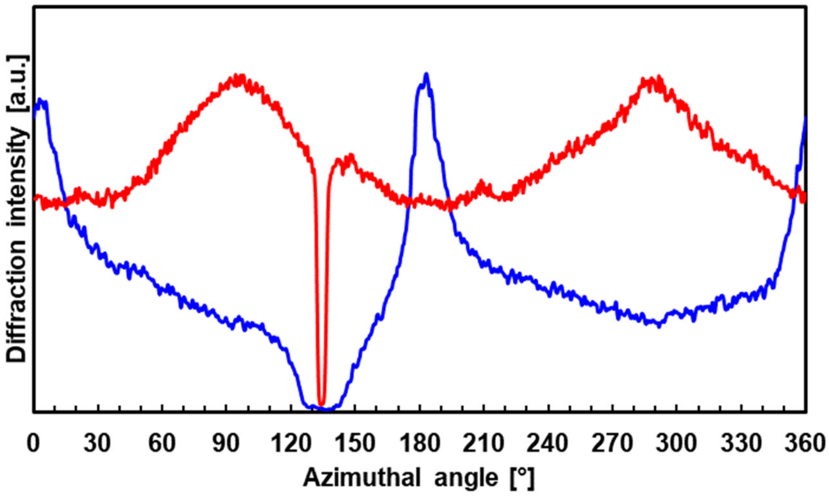

Figure S31. Azimuthal profiles of the diffraction for **P3** in the small-angle region (blue line) and wide-angle region (red line) at 30 °C on cooling.

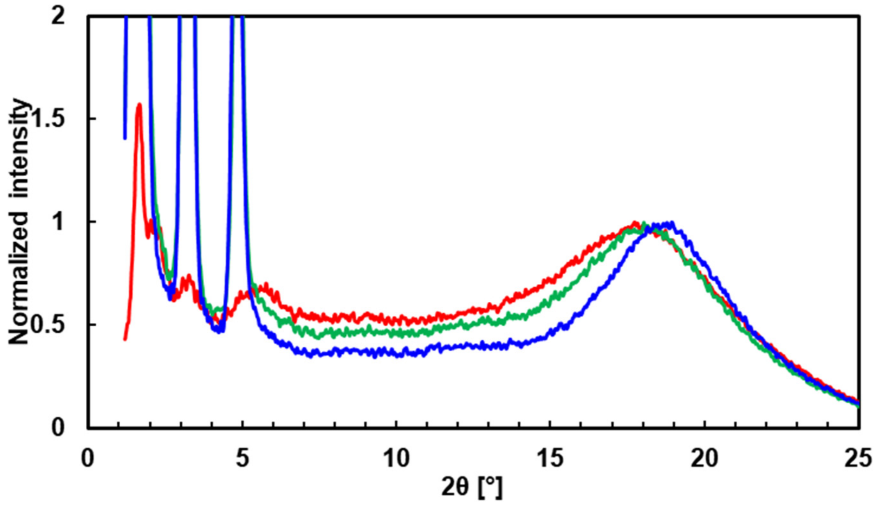

Figure S32. WAXD intensity profiles of **P4** at 30 °C (blue line), 160 °C (green line), and 190 °C (red line) on cooling.

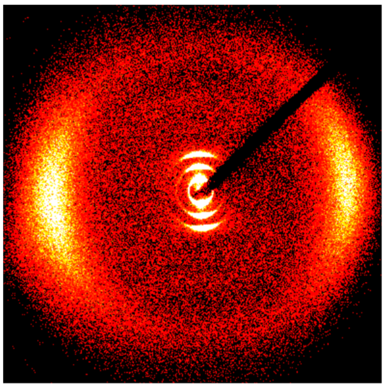

Figure S33. 2D-WAXD pattern for **P4** at 30 °C on cooling.

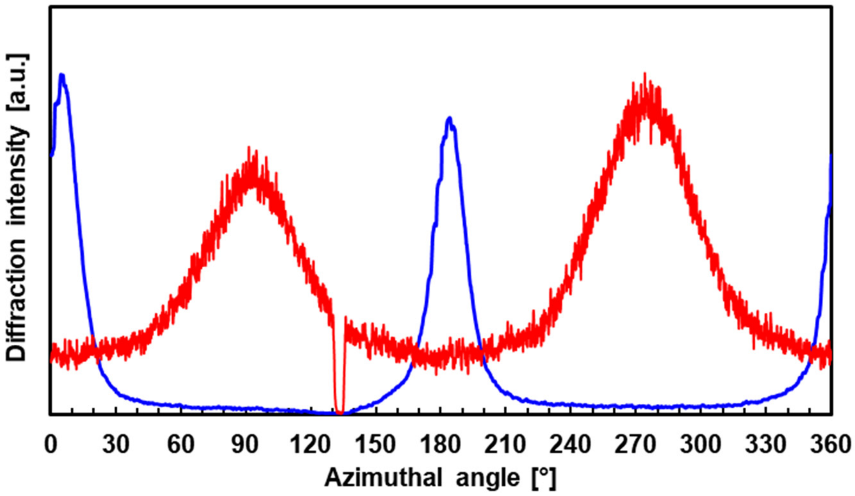

Figure S34. Azimuthal profiles of the diffraction for **P4** in the small-angle region (blue line) and wide-angle region (red line) at 30 °C on cooling.

Theoretical calculation

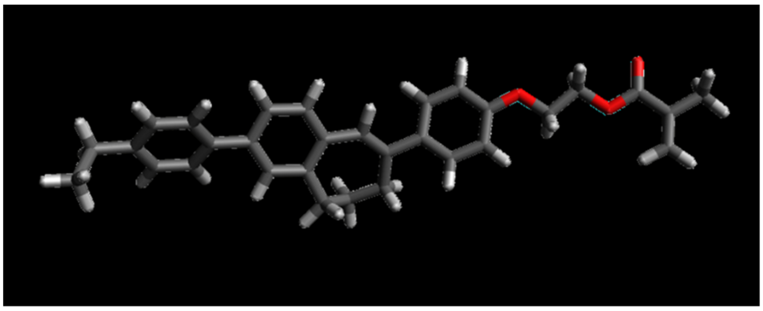

Figure 35. Optimized structure of **M5** using DFT calculation (B3LYP/6-31G(d) level).

Table S1. Atom coordinates and absolute energy of **M5** in theoretical calculation.

**M5** (Ground): *E* (B3LYP/6-31G(d)) = -1426.24781190 Hartree			
Atom	Coordinates (Angstroms)		
	X	Y	Z
C	2.56616	-0.06339	-0.1435
C	3.39074	1.07369	0.04301
C	4.77687	0.91207	0.08358
H	5.39079	1.79422	0.24858
C	4.58462	-1.45908	-0.20159
H	5.0268	-2.44388	-0.32254
C	3.20513	-1.31678	-0.24824
H	2.58947	-2.20257	-0.38724
C	1.09936	-0.08497	-0.2083
H	0.71651	-1.09944	-0.11044
C	0.14268	0.86402	-0.36282
C	-1.28365	0.42855	-0.40468
C	-1.67298	-0.77679	-1.02537
H	-0.92248	-1.38883	-1.51645
C	-2.30647	1.20987	0.15517
H	-2.06414	2.14884	0.6433
C	2.81783	2.46022	0.22131
H	2.36404	2.54969	1.22019
H	3.64331	3.18097	0.19965
C	0.35732	2.35832	-0.50764
H	0.02985	2.85442	0.41978

H	-0.33033	2.72392	-1.28236
C	1.76886	2.84595	-0.83534
H	1.7291	3.93931	-0.91891
H	2.08126	2.47011	-1.81764
C	-2.99716	-1.18831	-1.05908
H	-3.28533	-2.11305	-1.54951
C	-3.64456	0.80987	0.13396
H	-4.39381	1.44426	0.59421
C	-3.99612	-0.39857	-0.47382
C	5.40917	-0.33593	-0.03643
C	6.88571	-0.45833	0.01593
C	7.50293	-1.56536	0.62279
C	7.71989	0.52405	-0.54457
C	8.89012	-1.6816	0.66671
H	6.88838	-2.33309	1.08491
C	9.10654	0.40336	-0.49696
H	7.27569	1.37634	-1.0514
C	9.72063	-0.69999	0.11027
H	9.33724	-2.54957	1.14739
H	9.72452	1.17471	-0.9525
C	11.22806	-0.80792	0.19683
C	11.80422	-0.14587	1.46189
H	11.68053	-0.34404	-0.68897
H	11.52209	-1.86511	0.17892
H	11.55531	0.92082	1.49441
H	11.39638	-0.60898	2.36744
O	-5.27109	-0.88753	-0.55941
C	-6.32942	-0.12628	0.00193
C	-7.6008	-0.91925	-0.25513
H	-6.39569	0.8634	-0.47062
H	-6.18171	0.01683	1.08119
H	-7.76743	-1.06266	-1.32667
H	-7.55369	-1.90531	0.21606
H	12.89581	-0.24205	1.49199
O	-8.67208	-0.14914	0.31395
C	-9.91027	-0.68916	0.18771

O	-10.112	-1.75112	-0.3672
C	-10.9913	0.14516	0.79381
C	-10.7177	1.3091	1.39653
H	-11.5086	1.91142	1.83574
H	-9.70565	1.69066	1.46831
C	-12.3767	-0.4315	0.66585
H	-12.6434	-0.57868	-0.38674
H	-12.435	-1.41792	1.13943
H	-13.117	0.22677	1.12889

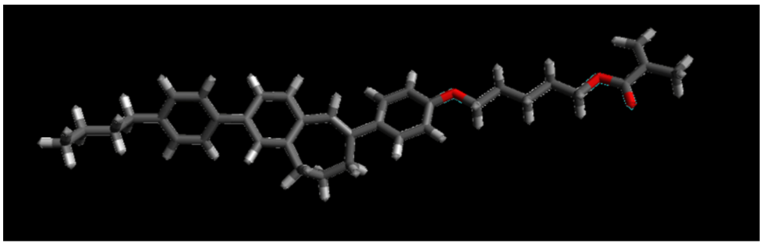

Figure S36. Optimized structure of **M6** using DFT calculation (B3LYP/6-31G(d) level).

Table S2. Atom coordinates and absolute energy of **M6** in theoretical calculation.

**M6** (Ground): *E* (B3LYP/6-31G(d)) = -1622.81975819 Hartree			
Atom	Coordinates (Angstroms)		
	X	Y	Z
C	-3.26025	0.01815	0.37201
C	-4.11974	1.11071	0.09741
C	-5.50102	0.90773	0.09102
H	-6.14205	1.75438	-0.14271
C	-5.23693	-1.4223	0.58227
H	-5.64897	-2.40439	0.79623
C	-3.86169	-1.23812	0.59661
H	-3.21904	-2.09056	0.80485
C	-1.79275	0.0429	0.41651
H	-1.38212	-0.96528	0.40227
C	-0.86166	1.02772	0.47308
C	0.57701	0.63789	0.53224
C	1.00885	-0.49997	1.24564
H	0.28229	-1.08983	1.79646

C	1.57074	1.39771	-0.10429
H	1.2959	2.28552	-0.66565
C	-3.58913	2.4912	-0.21098
H	-3.14968	2.50406	-1.22015
H	-4.4347	3.18799	-0.24152
C	-1.11828	2.52237	0.48907
H	-0.81607	2.94345	-0.48293
H	-0.4325	2.97401	1.21872
C	-2.53936	2.99783	0.79248
H	-2.53017	4.095	0.77874
H	-2.82939	2.70199	1.80844
C	2.34462	-0.86942	1.29512
H	2.66498	-1.74146	1.85709
C	2.92002	1.03865	-0.06844
H	3.64449	1.65358	-0.5903
C	3.31613	-0.1049	0.63268
C	-6.0956	-0.34172	0.32985
C	-7.56846	-0.50933	0.30883
C	-8.16028	-1.68234	-0.18979
C	-8.42455	0.49512	0.79184
C	-9.54391	-1.84053	-0.20459
H	-7.52897	-2.47044	-0.59101
C	-9.80758	0.33228	0.77367
H	-7.99962	1.40099	1.21545
C	-10.3965	-0.83708	0.27437
H	-9.97104	-2.75973	-0.60077
H	-10.4426	1.12335	1.16777
C	-11.9003	-0.99362	0.21741
C	-12.5153	-0.4644	-1.0943
H	-12.3586	-0.4644	1.06381
H	-12.166	-2.05308	0.33285
C	-14.0397	-0.62148	-1.15316
H	-12.2485	0.59513	-1.21405
H	-12.0569	-0.99036	-1.9436
C	-14.6485	-0.09507	-2.45688
H	-14.2998	-1.68246	-1.02909

H	-14.4914	-0.09658	-0.29924
H	-15.7372	-0.22083	-2.46943
H	-14.4347	0.97237	-2.59151
H	-14.2417	-0.62521	-3.32675
O	4.60257	-0.54963	0.74166
C	5.63954	0.18912	0.10194
C	6.9561	-0.52207	0.38584
H	5.66174	1.21895	0.48886
H	5.45123	0.24186	-0.98085
C	8.15535	0.18534	-0.25763
H	7.09358	-0.58472	1.47279
H	6.88406	-1.55325	0.01739
C	9.4838	-0.52853	0.02615
H	8.00207	0.25309	-1.34449
H	8.21142	1.22056	0.10914
H	9.65097	-0.59119	1.10922
H	9.44119	-1.56015	-0.34647
C	10.6646	0.18252	-0.61736
H	10.56451	0.23644	-1.70719
H	10.77507	1.20799	-0.24741
O	11.85478	-0.56451	-0.2919
O	13.06768	0.93815	-1.4661
C	13.01264	-0.0702	-0.78758
C	14.21473	-0.88081	-0.42187
C	14.10785	-1.98492	0.32793
H	13.14958	-2.33212	0.69715
H	14.98492	-2.57055	0.5909
C	15.51805	-0.35287	-0.96114
H	15.49495	-0.29762	-2.05531
H	15.70426	0.66673	-0.60544
H	16.35288	-0.99072	-0.65741

4. *Optical properties*

Birefringence

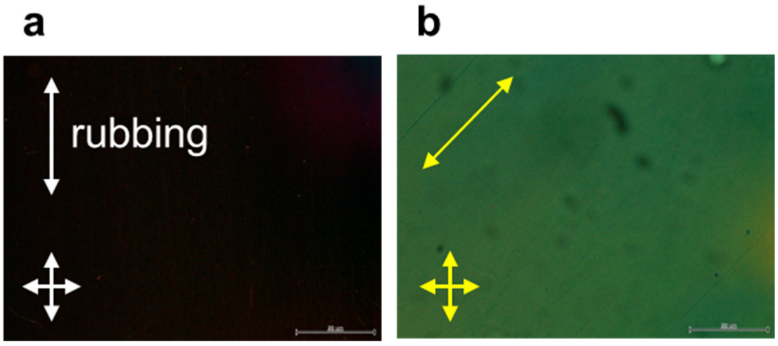

Figure S37. POM images of uniaxially aligned **P1** in nematic phase in which the rubbing direction is (a) parallel and (b) 45 degrees to the polarization direction.

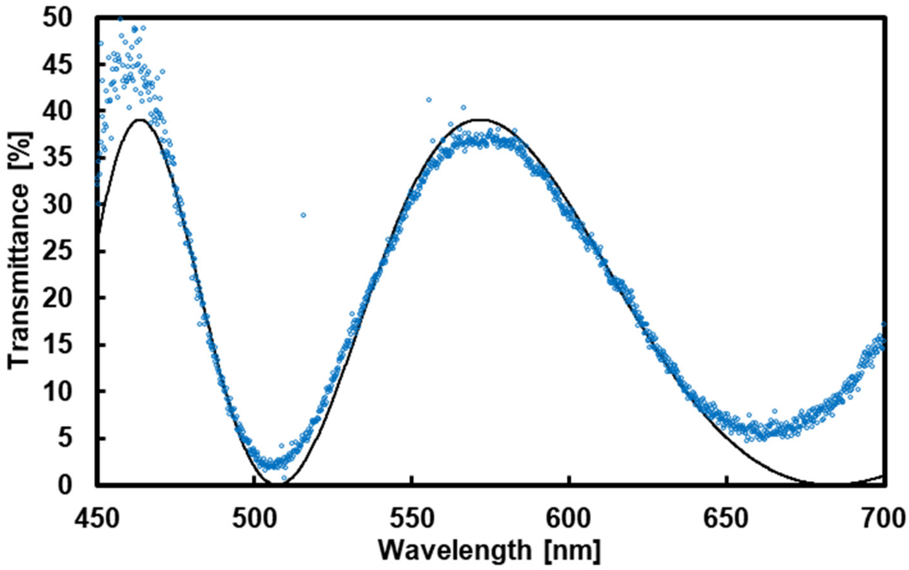

Figure S38. Wavelength dependence of light intensity (dots) transmitted through a homogeneous nematic cell of **P1** under cross-polarization conditions. The solid curve is the fitting of eqn (1).

Fluorescence properties

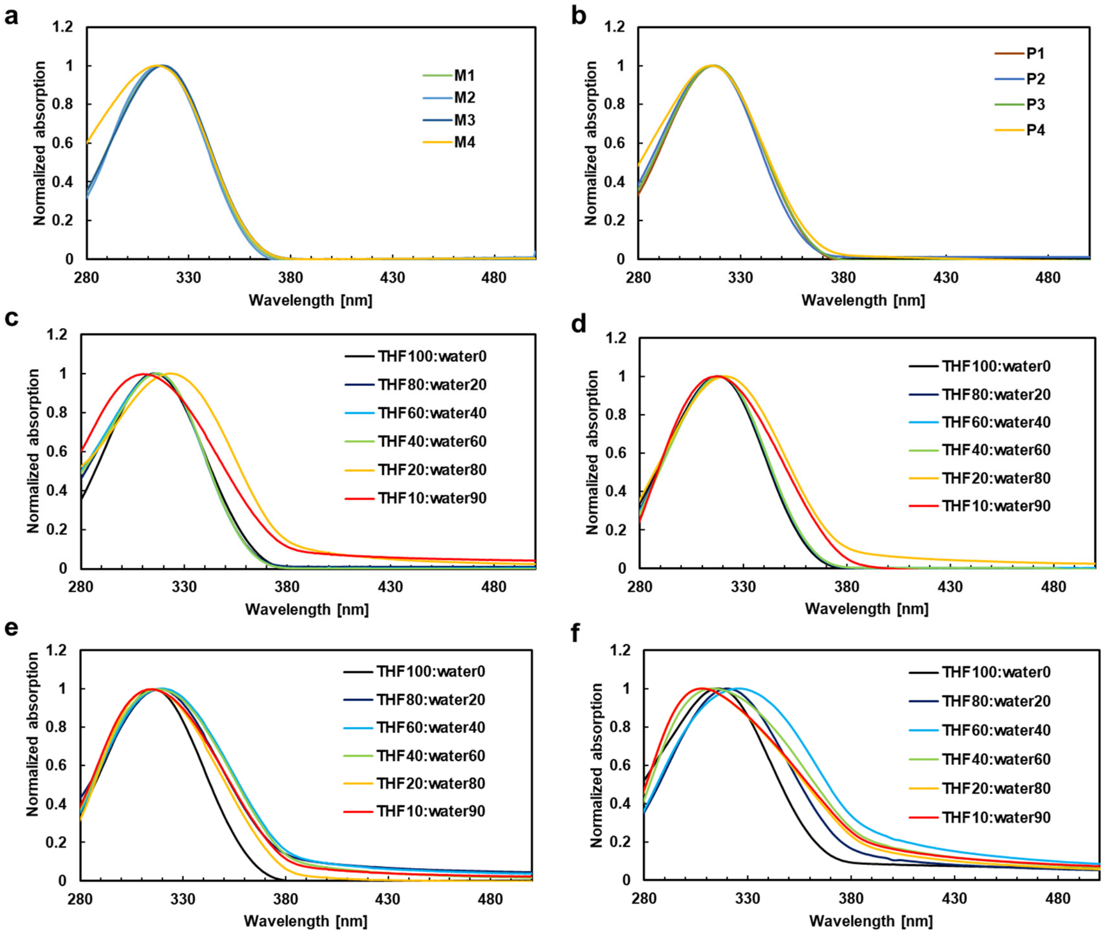

Figure S39. Absorption spectra in THF for (a) **M1-M4** and (b) **P1-P4**, and in THF/water mixtures with different water contents (vol%) for (c) **M2**, (d) **P2**, (e) **M4**, and (f) **P4**; concentration is 1×10^{-5} mol L^{-1}.

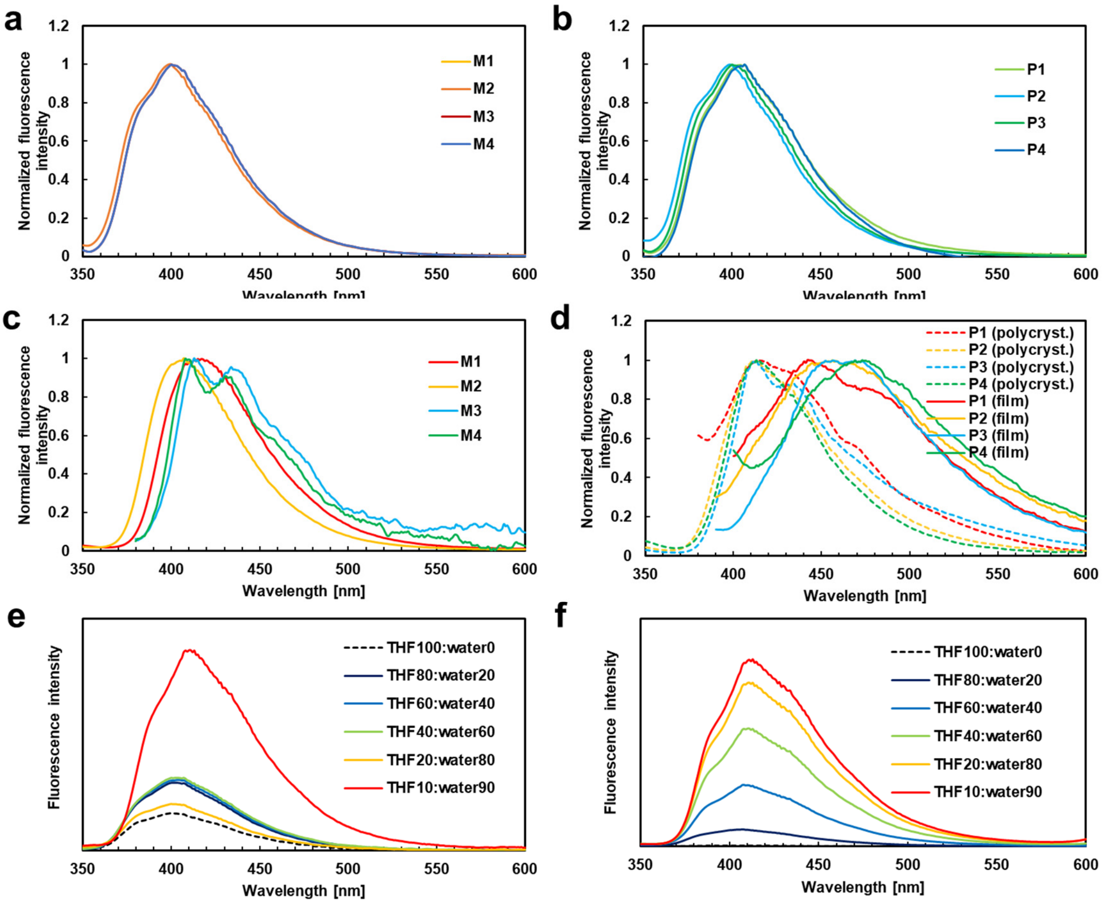

Figure S40. Fluorescence spectra excited at each λ_{abs} in THF for (a) **M1-M4** and (b) **P1-P4**, in solid (polycrystalline) state (or film state) for (c) **M1-M4** and (d) **P1-P4**, and in THF/water mixtures with different water contents (vol%) for (c) **M4** and (d) **P4** (solution concentration is 1 × 10^{-5} mol L^{-1}).

Table S3. Fluorescence quantum yield Φ_{fl} for **M2**, **M4**, **P2**, and **P4** in THF/water mixtures with different water contents (vol%) at the concentration of 1 × 10^{-5} mol L^{-1}).

THF [%]	water [%]	Φ_{fl}			
		M2	P2	M4	P4
100	0	0.02	0.06	0.01	0.03
80	20	0.02	0.07	0.02	0.04
60	40	0.03	0.21	0.02	0.10
40	60	0.03	0.23	0.02	0.13
20	80	0.08	0.25	0.02	0.17
10	90	0.12	0.19	0.09	0.18

5. Spectra chart

NMR chart

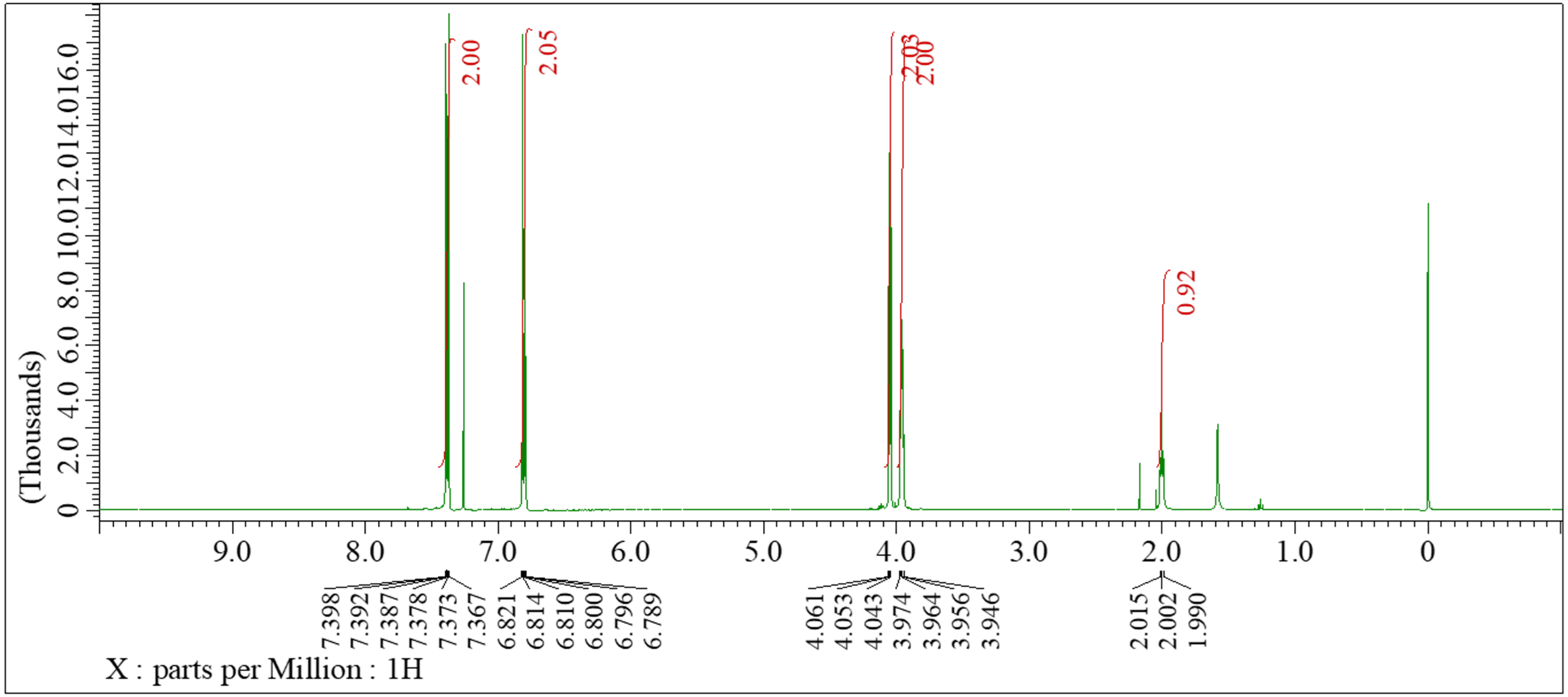

Figure S41. ^{1}H-NMR spectra of **2-(4-bromophenoxy)ethan-1-ol (2a)** (500Hz, $CDCl_3$).

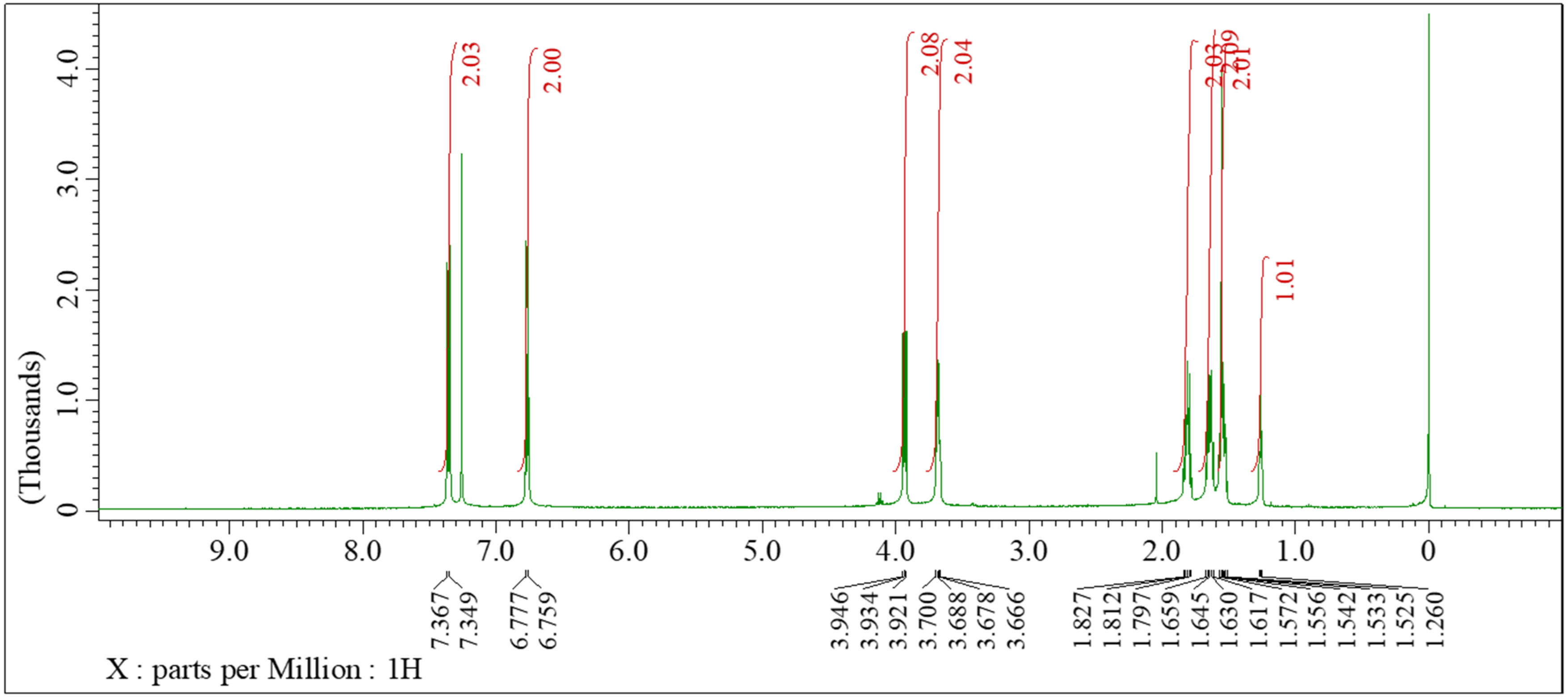

Figure S42. ^{1}H-NMR spectra of **5-(4-bromophenoxy)pentan-1-ol (2b)** (500Hz, $CDCl_3$).

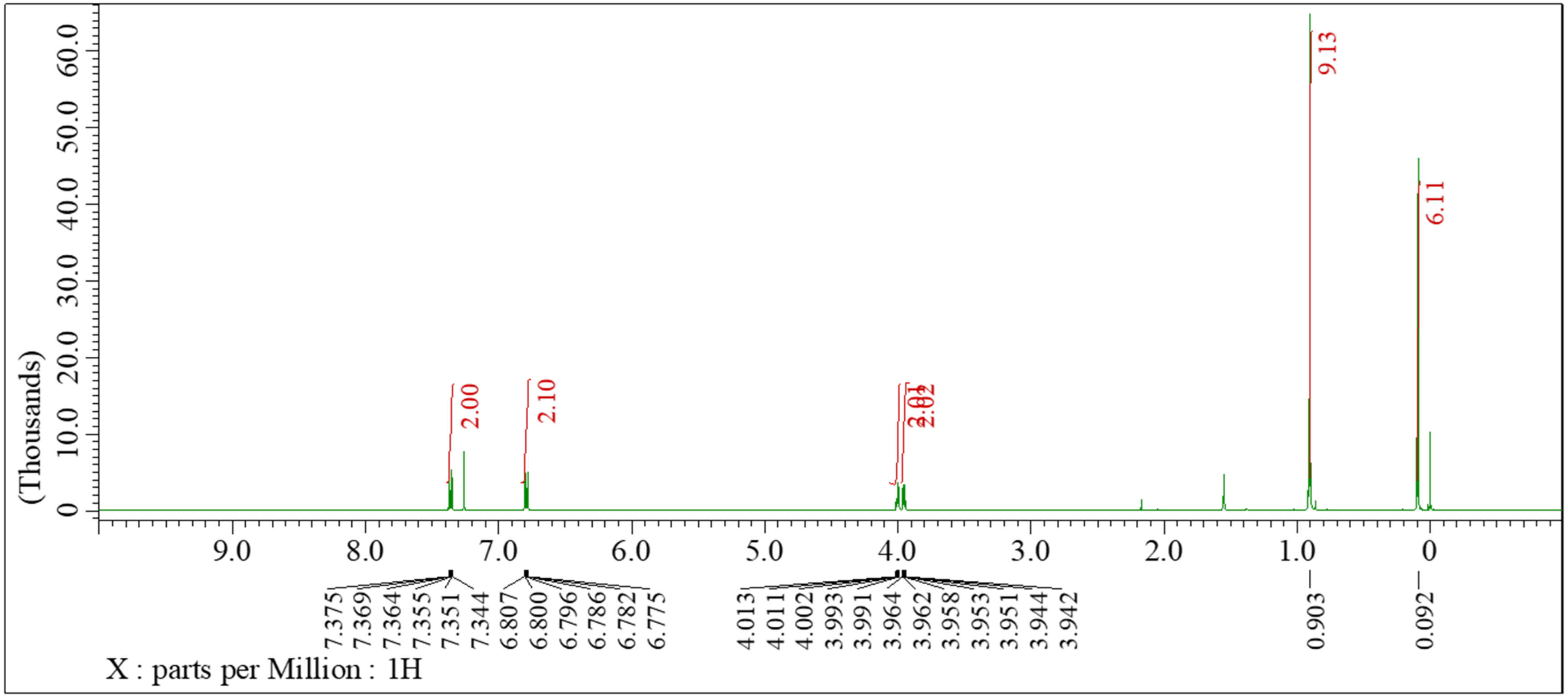

Figure S43. ^{1}H-NMR spectra of **(2-(4-bromophenoxy)ethoxy)(*tert*-butyl)dimethylsilane (3a)** (500Hz, $CDCl_3$).

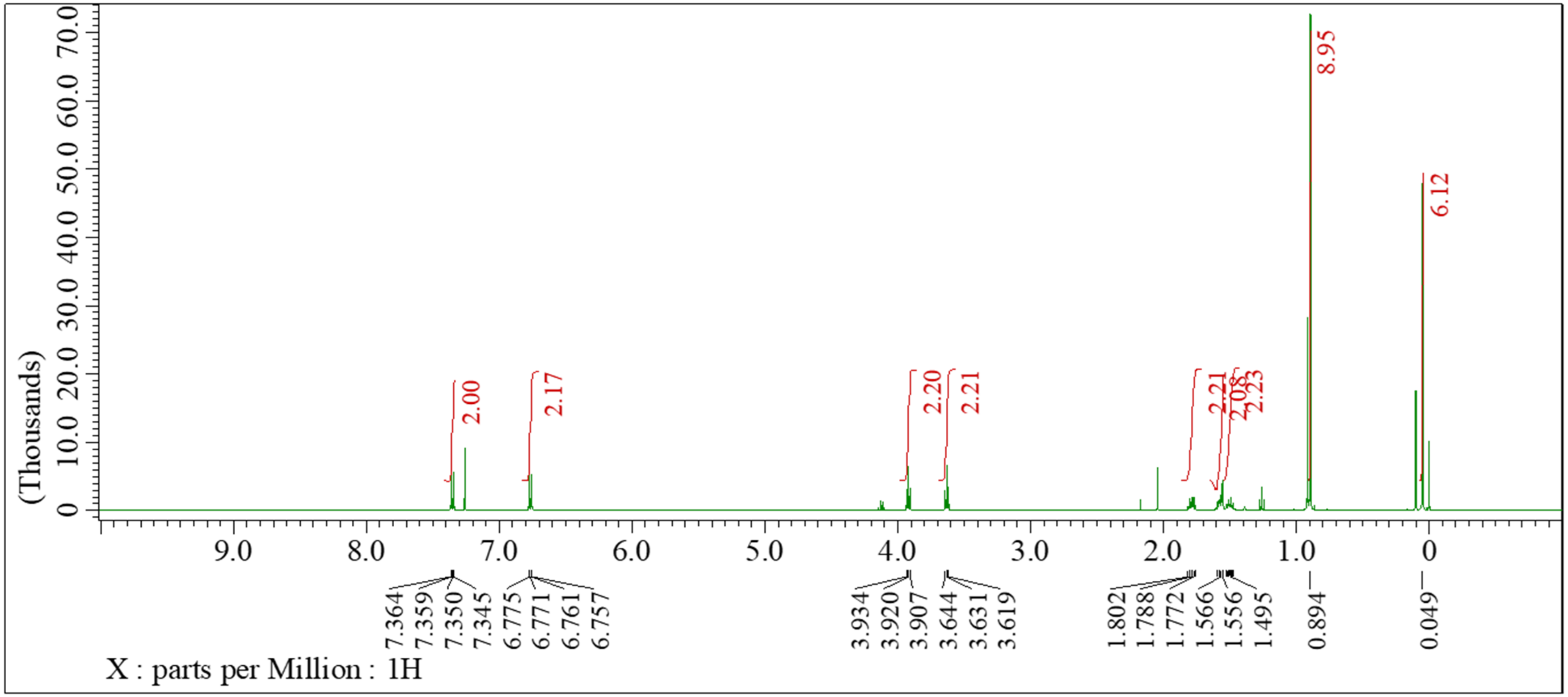

Figure S44. ^{1}H-NMR spectra of **((5-(4-bromophenoxy)pentyl)oxy)(*tert*-butyl)dimethylsilane (3b)** (500Hz, $CDCl_3$).

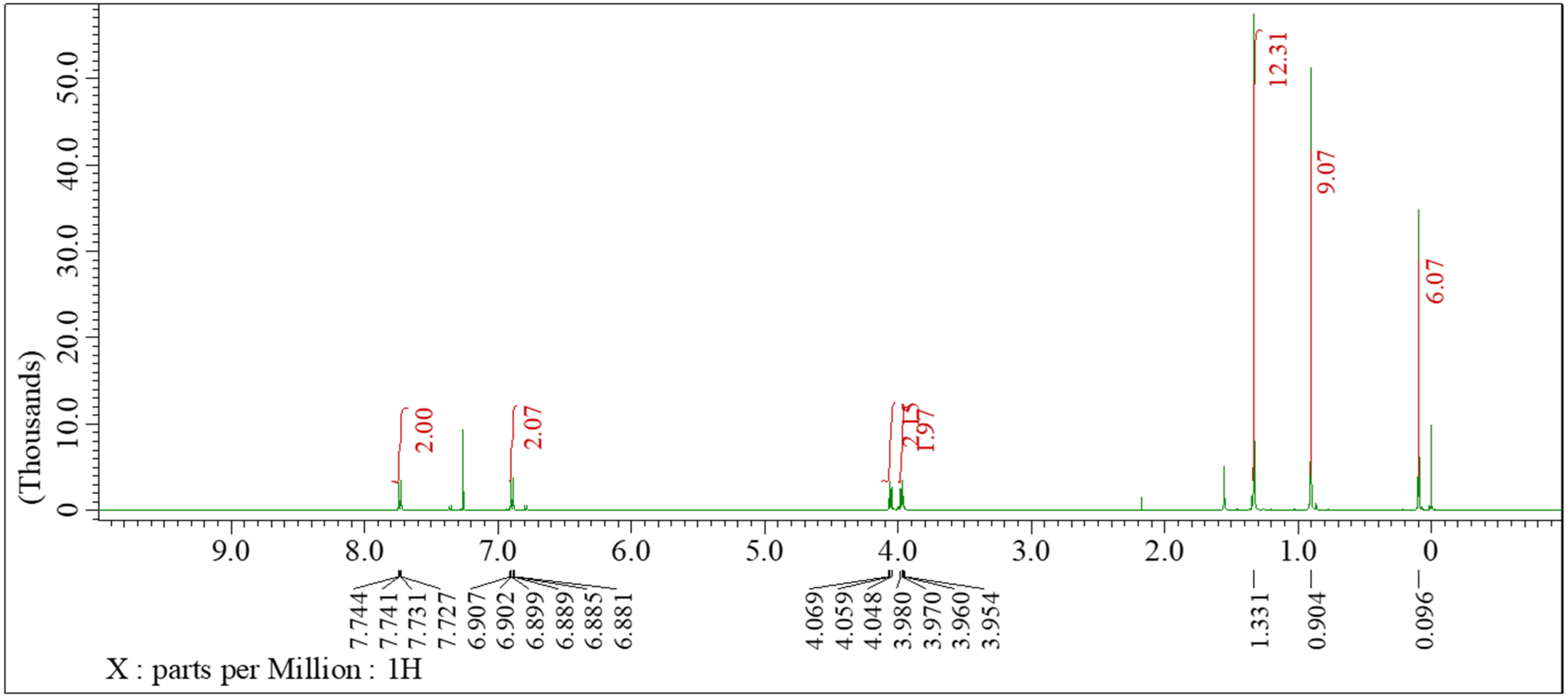

Figure S45. ^{1}H-NMR spectra of ***tert*-butyldimethyl(2-(4-(4,4,5,5-tetramethyl-1,3,2-dioxaborolan-2-yl)phenoxy)ethoxy)silane** (**4a**) (500Hz, $CDCl_3$).

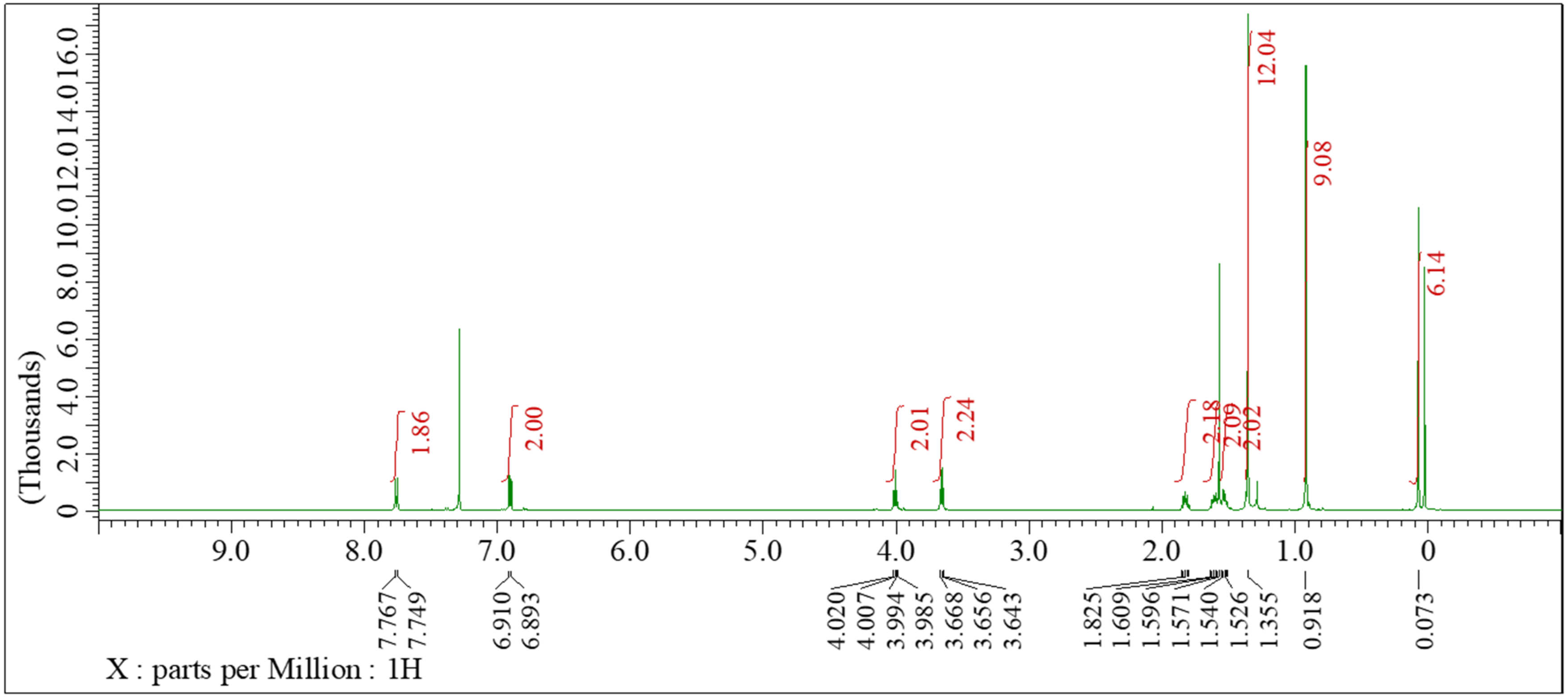

Figure S46. ^{1}H-NMR spectra of ***tert*-butyldimethyl((5-(4-(4,4,5,5-tetramethyl-1,3,2-dioxaborolan-2-yl)phenoxy)pentyl)oxy)silane (4b)** (500Hz, $CDCl_3$).

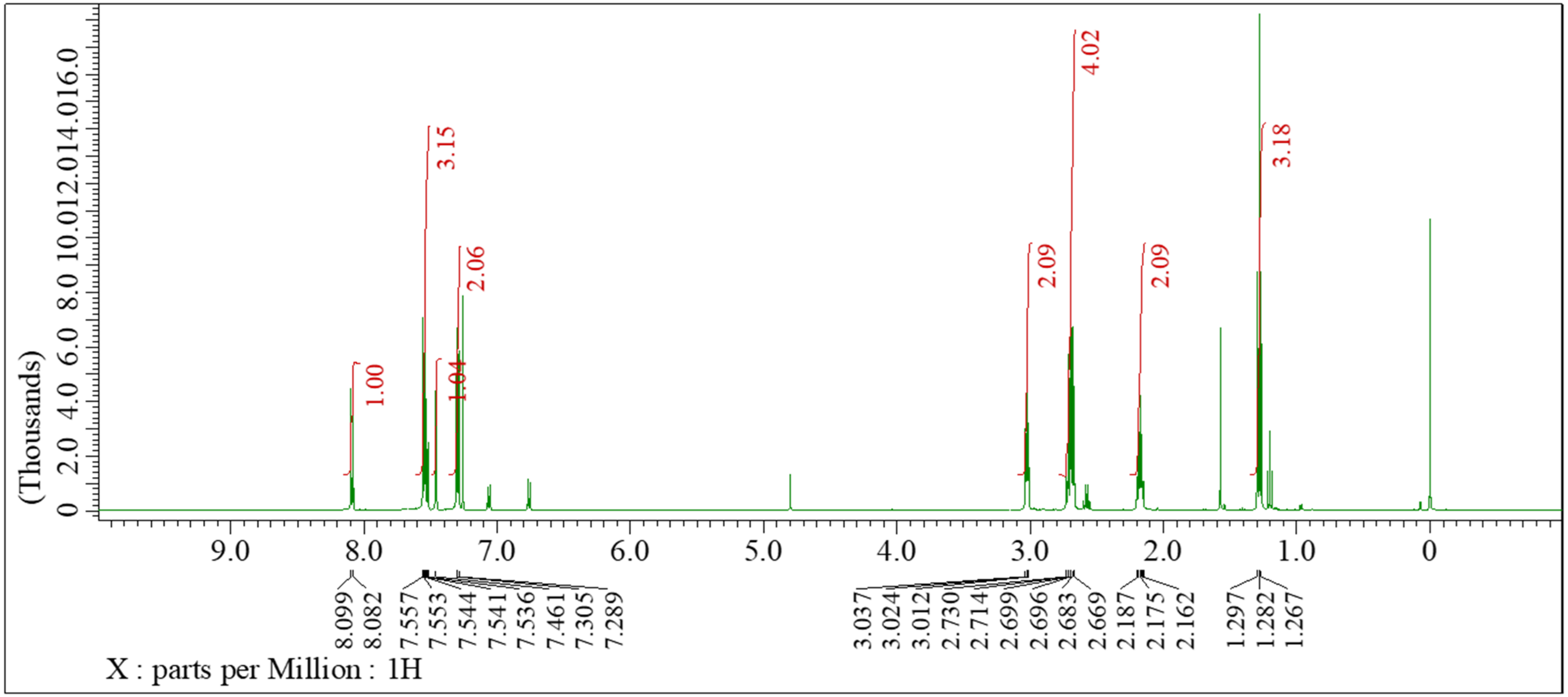

Figure S47. ^{1}H-NMR spectra of **6-(4-ethylphenyl)-3,4-dihydronaphthalen-1(2*H*)-one (6a)** (500Hz, $CDCl_3$).

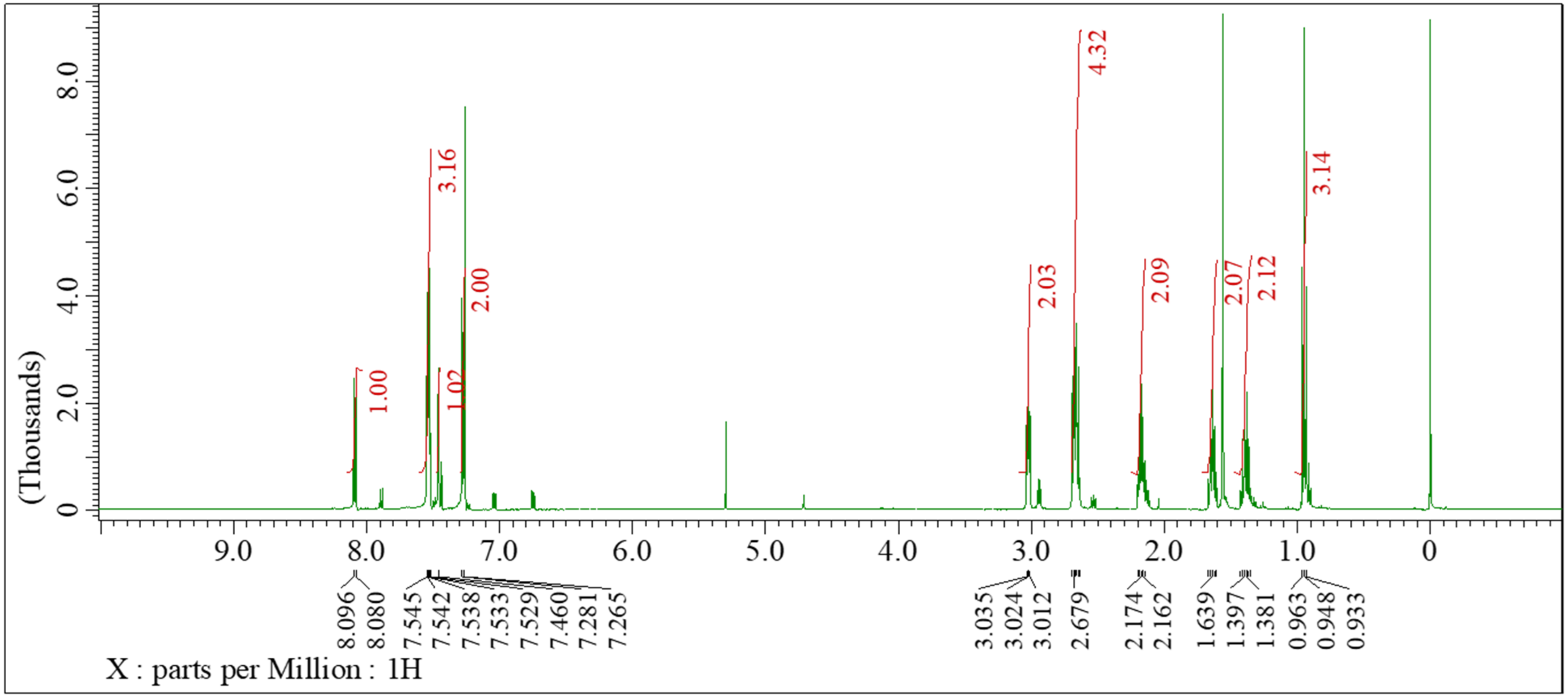

Figure S48. ^{1}H-NMR spectra of **6-(4-butylphenyl)-3,4dihydronaphthalene-1(2*H*)-one (6b)** (500Hz, $CDCl_3$).

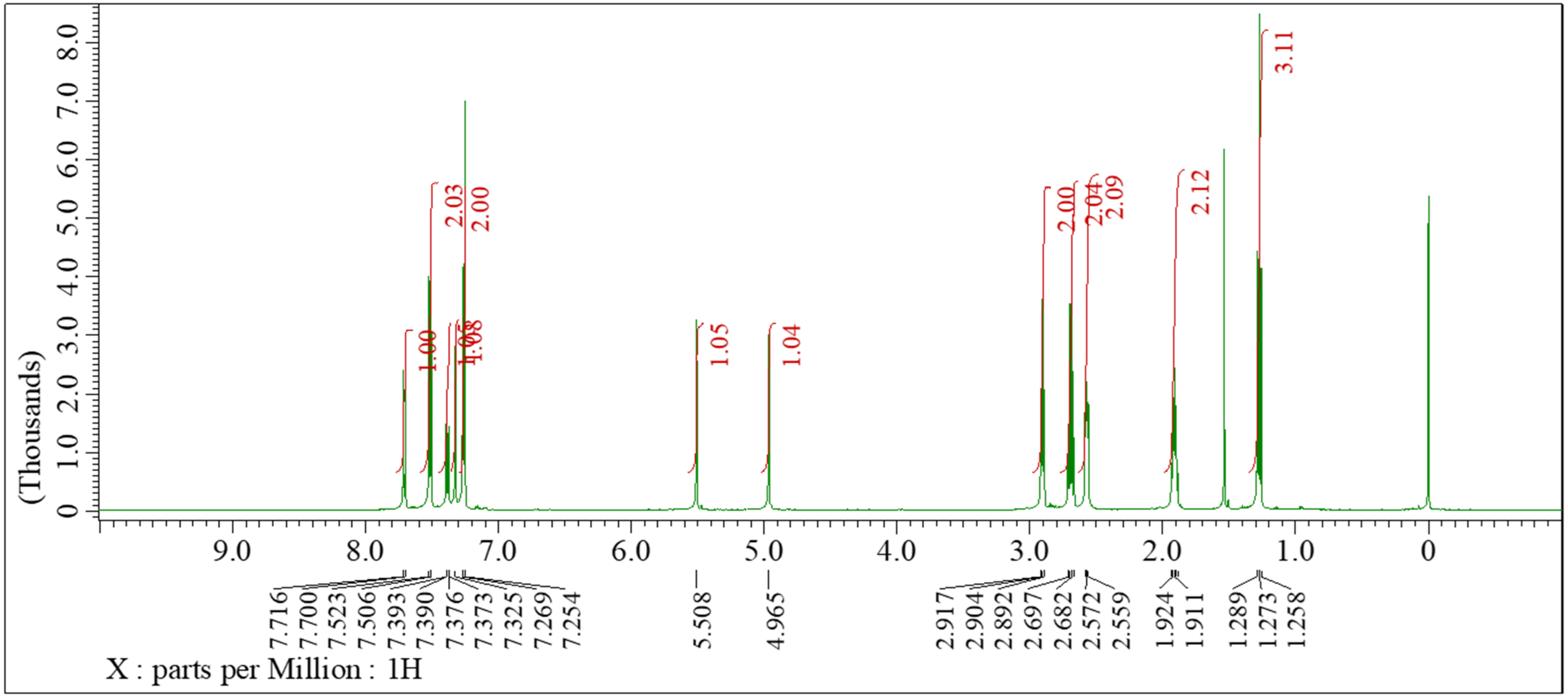

Figure S49. ^{1}H-NMR spectra of **6-(4-ethylphenyl)-1-methylene-1,2,3,4-tetrahydronaphthalene (7a)** (500Hz, $CDCl_3$).

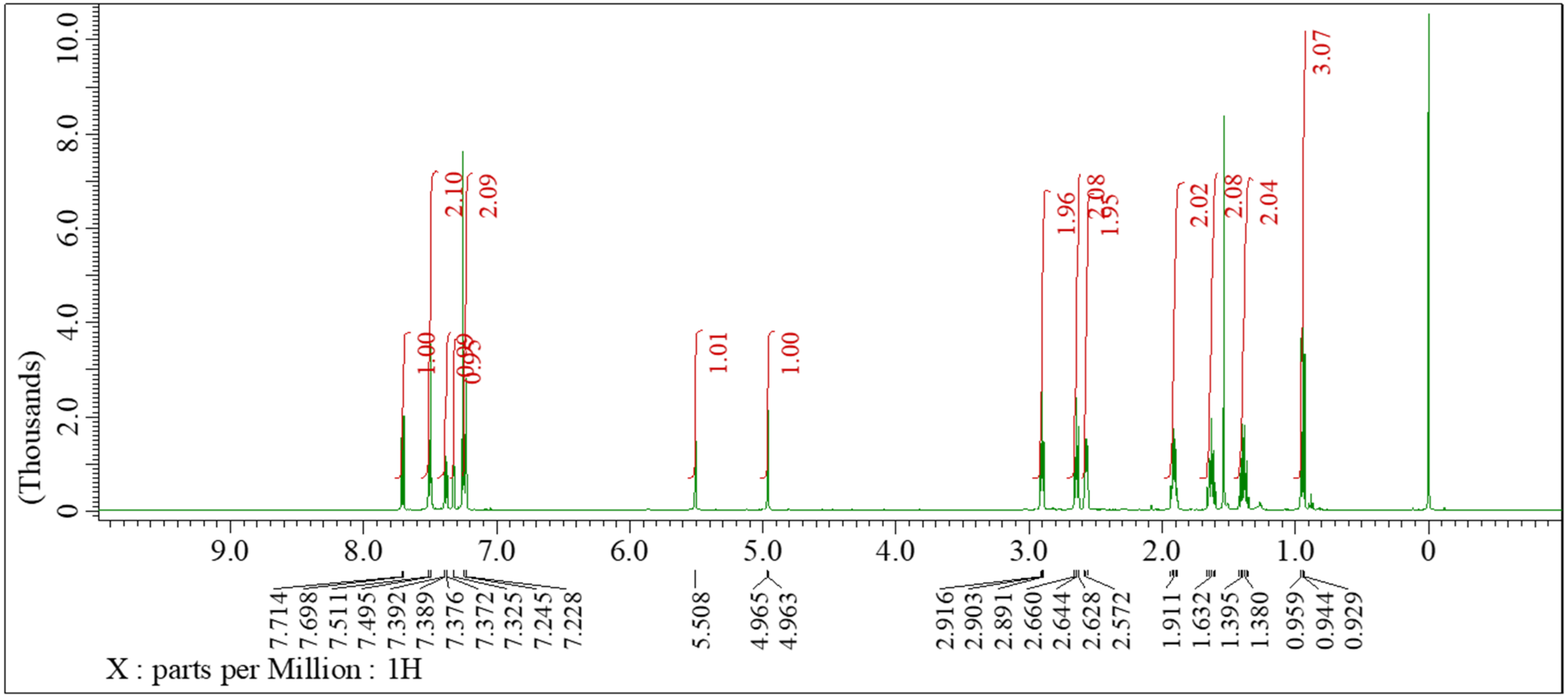

Figure S50. ^{1}H-NMR spectra of **6-(4-butylphenyl)-1-methylene-1,2,3,4-tetrahydronaphthalene (7b)** (500Hz, $CDCl_3$).

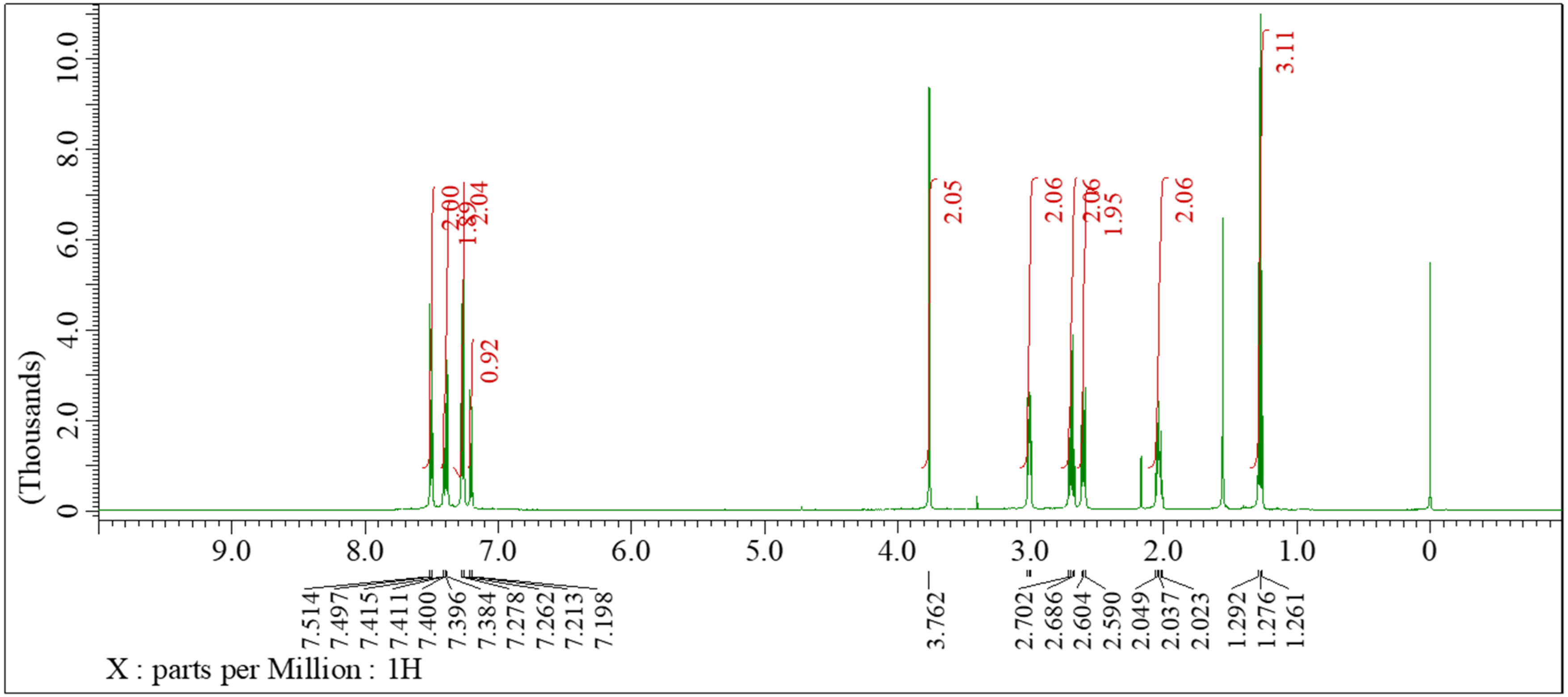

Figure S51. ¹H-NMR spectra of **2-(4-ethylphenyl)-5,7,8,9-tetrahydro-6*H*-benzo[7]annulen-6-one (8a)** (500Hz, $CDCl_3$).

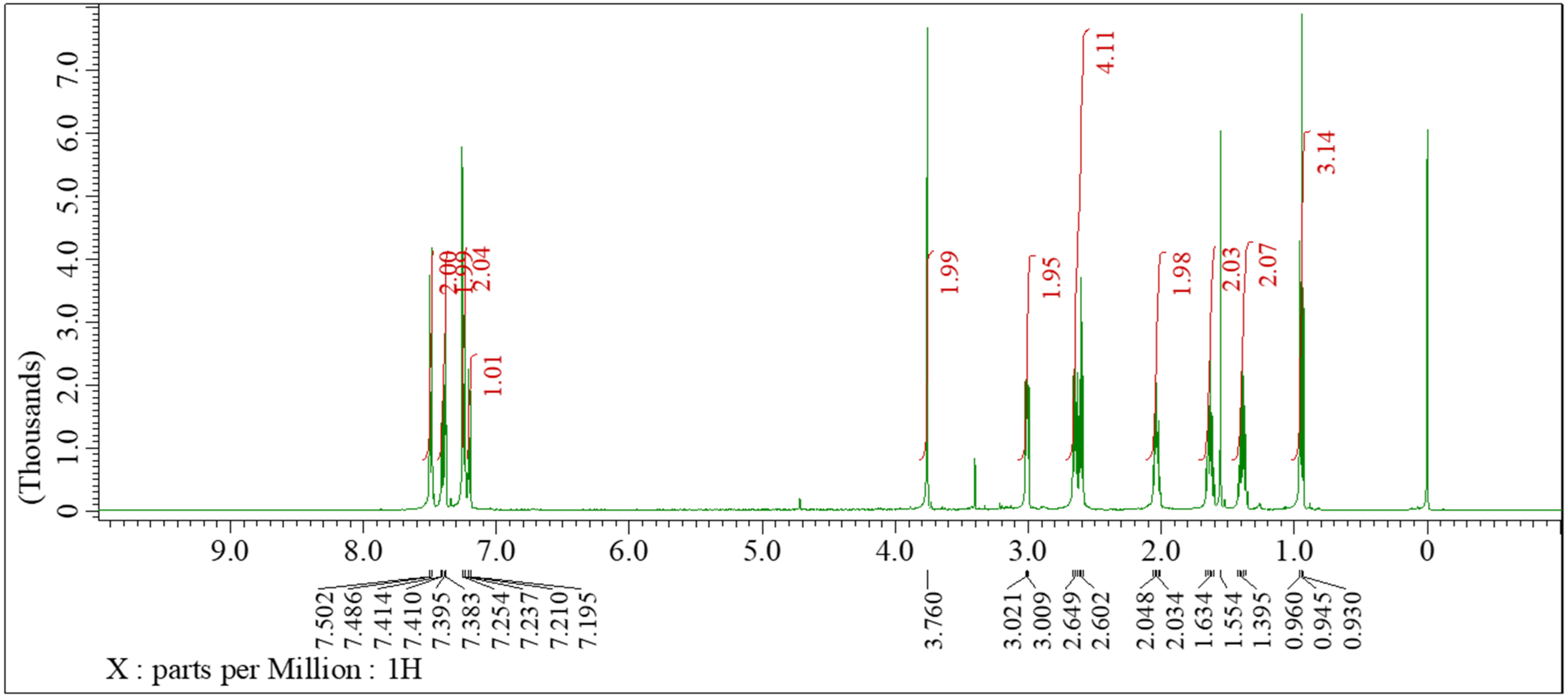

Figure S52. ^{1}H-NMR spectra of **2-(4-butylphenyl)-5,7,8,9-tetrahydro-6*H*-benzo[7]annulen-6-one (8b)** (500Hz, $CDCl_3$).

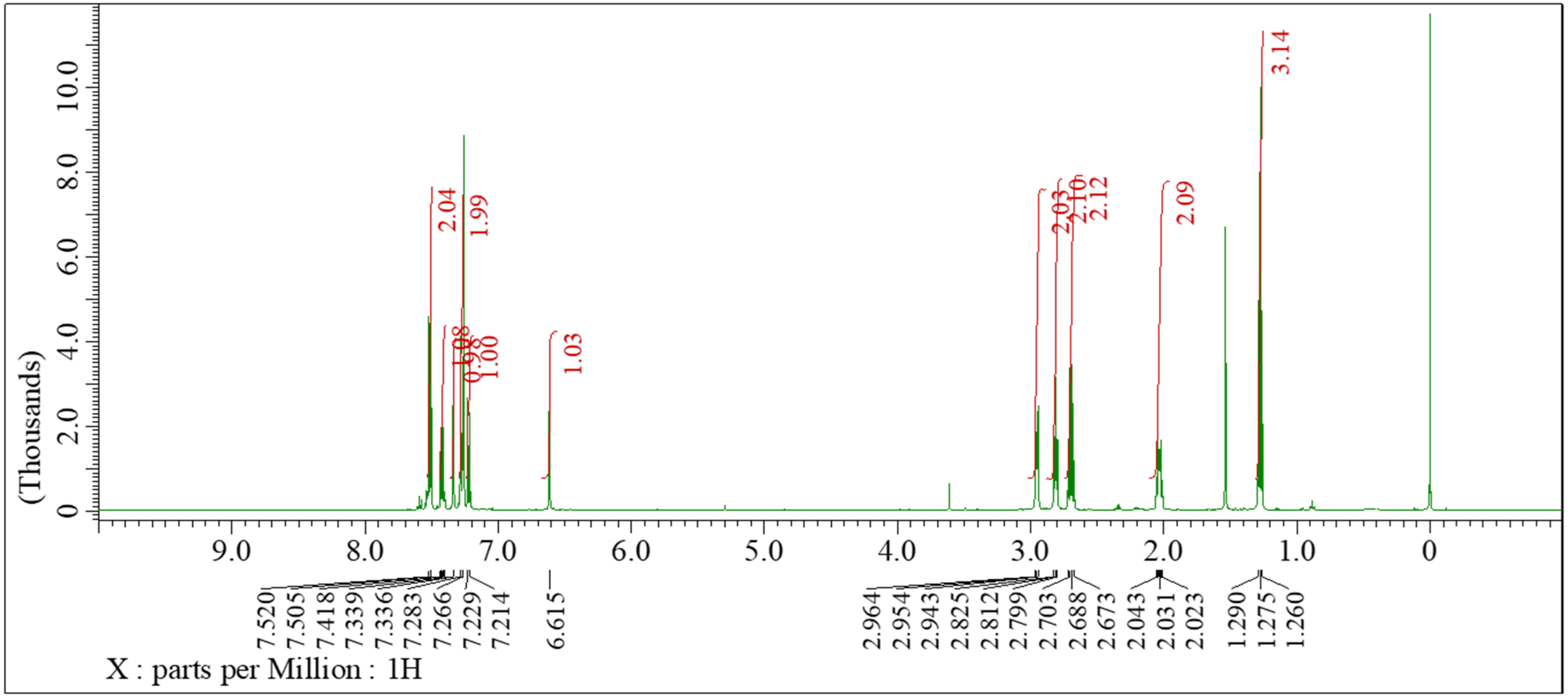

Figure S53. ^{1}H-NMR spectra of **3-(4-ethylphenyl)-6,7-dihydro-5*H*-benzo[7]annulen-8-yl trifluoromethanesulfonate (9a)** (500Hz, $CDCl_3$).

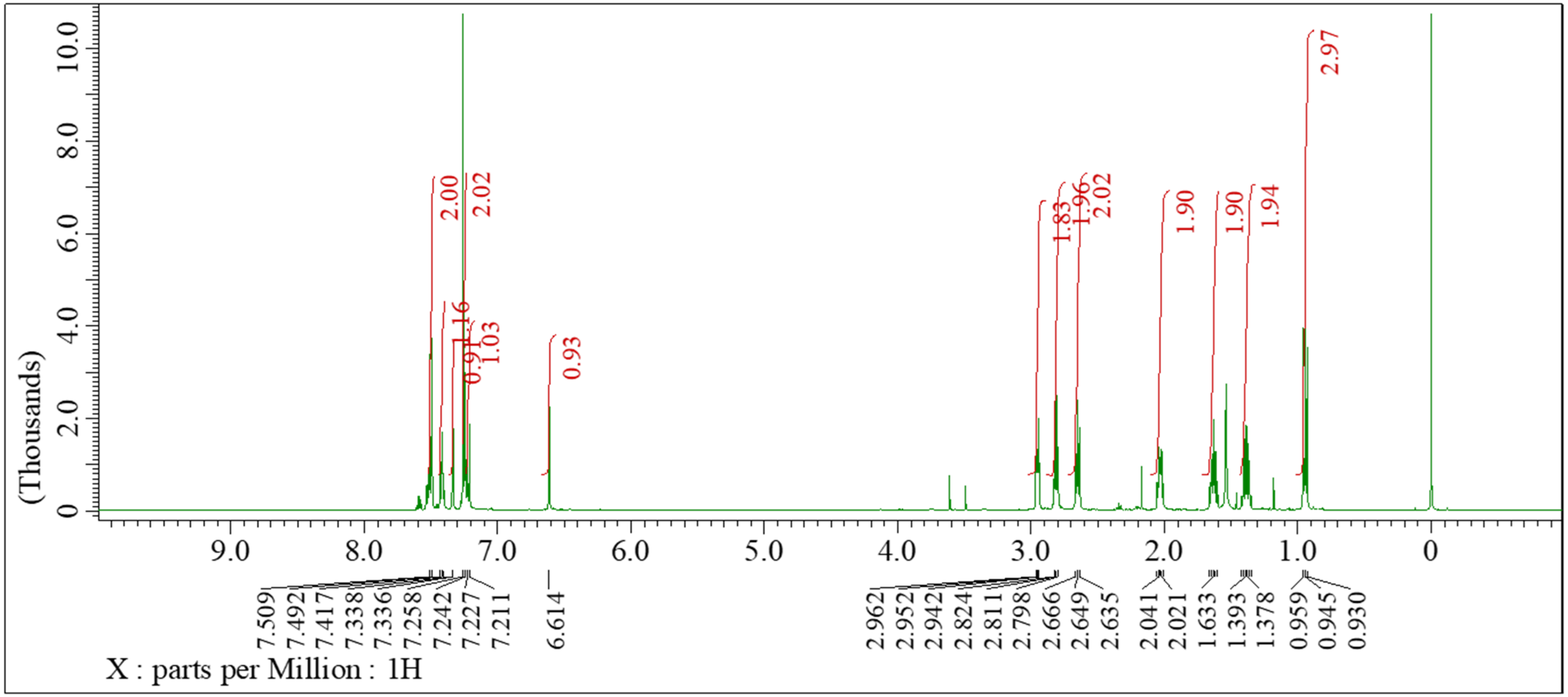

Figure S54. ^{1}H-NMR spectra of **3-(4-butylphenyl)-6,7-dihydro-5*H*-benzo[7]annulen-8-yltrifluoromethanesulfonate (9b)** (500Hz, $CDCl_3$).

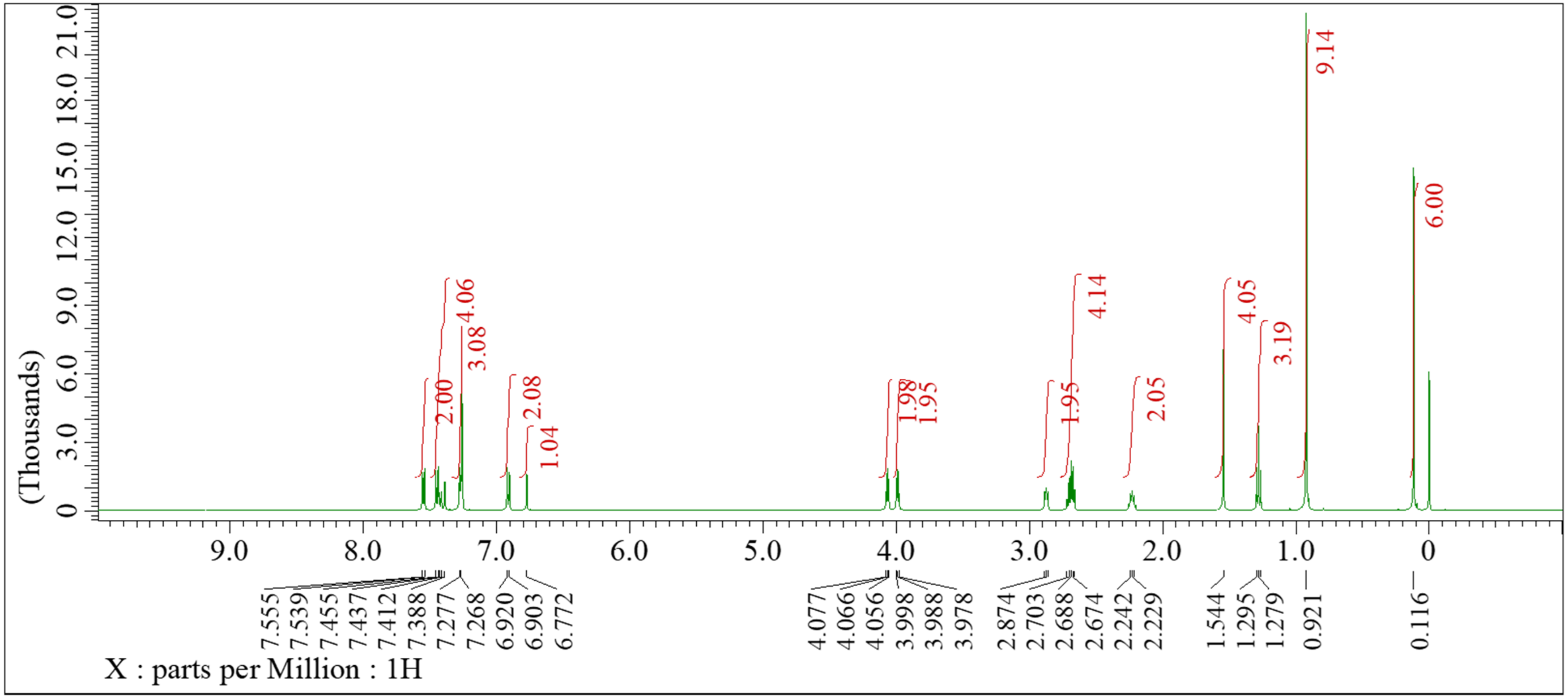

Figure S55. ^{1}H-NMR spectra of ***tert*-butyl(2-(4-(3-(4-ethylphenyl)-6,7-dihydro-5*H*-benzo[7]annulen-8-yl)phenoxy)ethoxy)dimethylsilane** **(10a)** (500Hz, $CDCl_3$).

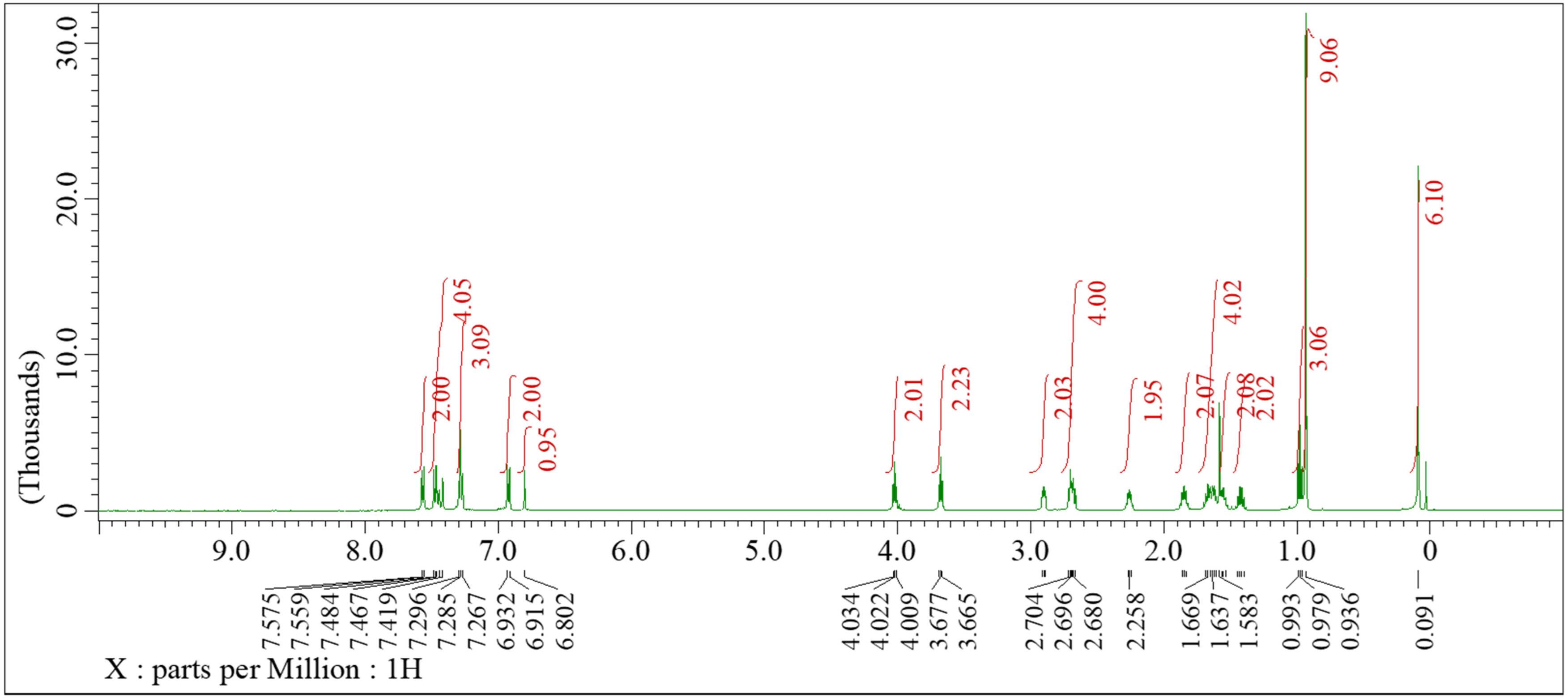

Figure S56. ^{1}H-NMR spectra of ***tert*-butyl((5-(4-(3-(4-butylphenyl)-6,7-dihydro-5*H*-benzo[7]annulen-8-yl)phenoxy)pentyl)oxy)dimethylsilane (10b)** (500Hz, $CDCl_3$).

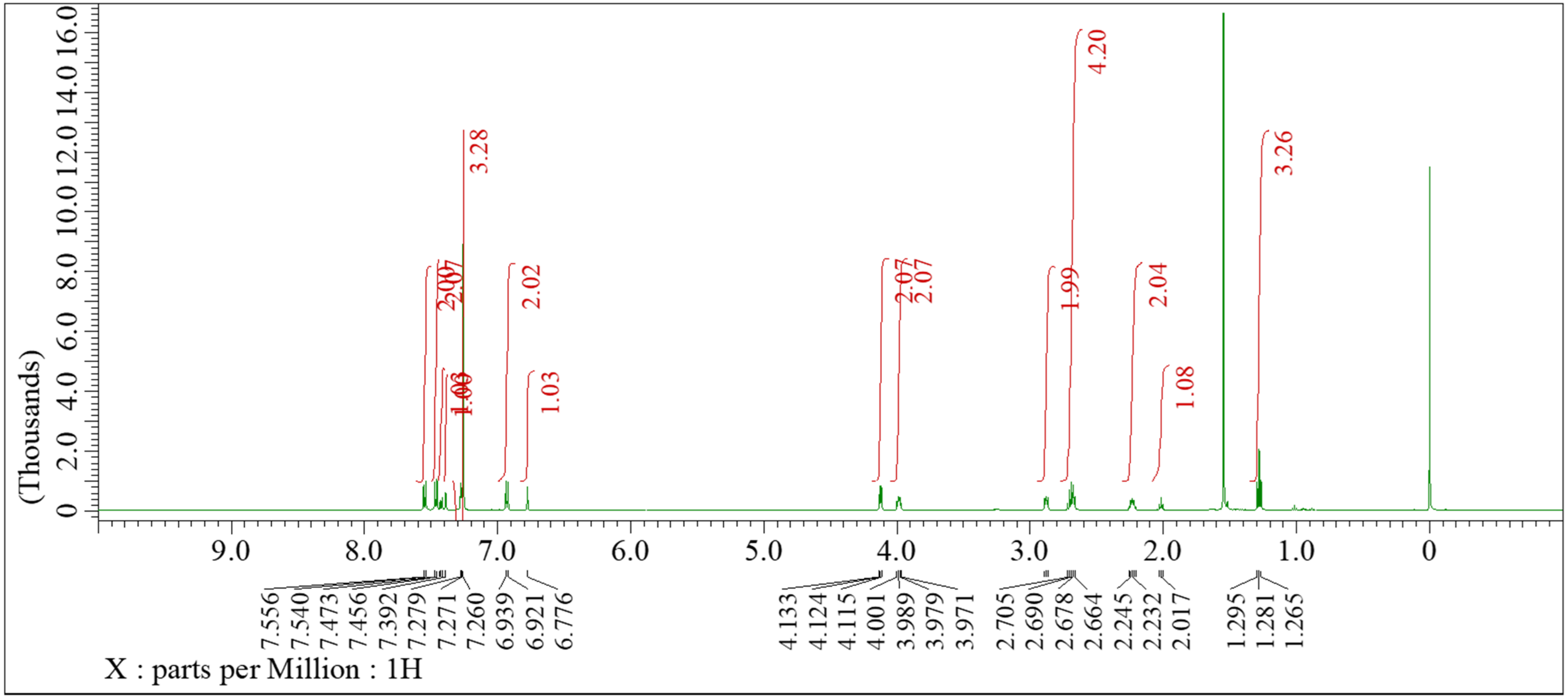

Figure S57. ^{1}H-NMR spectra of **2-(4-(3-(4-ethylphenyl)-6,7-dihydro-5*H*-benzo[7]annulen-8-yl)phenoxy)ethan-1-ol (11a)** (500Hz, $CDCl_3$).

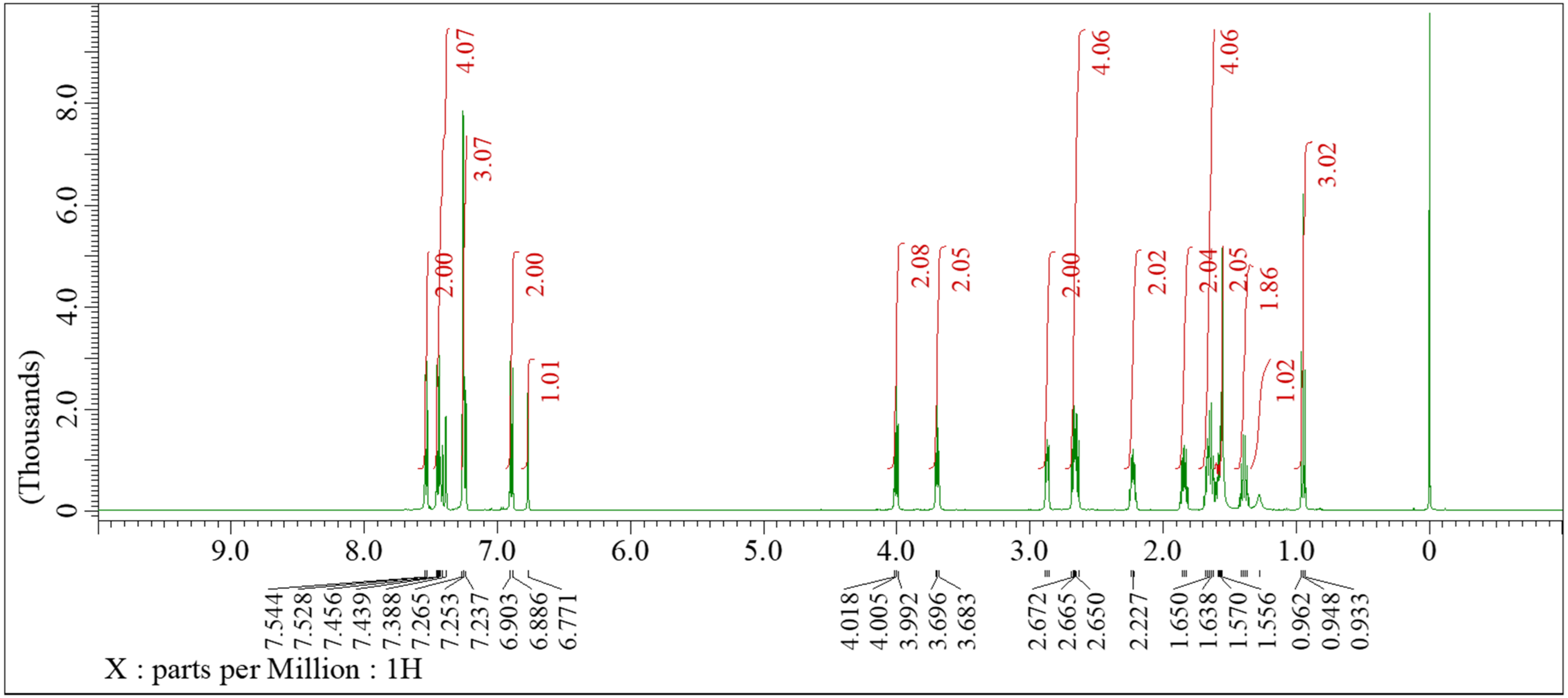

Figure S58. ^{1}H-NMR spectra of **5-(4-(3-(4-butylphenyl)-6,7-dihydro-5*H*-benzo[7]annulen-8-yl)phenoxy)pentan-1-ol (11b)** (500Hz, $CDCl_3$).

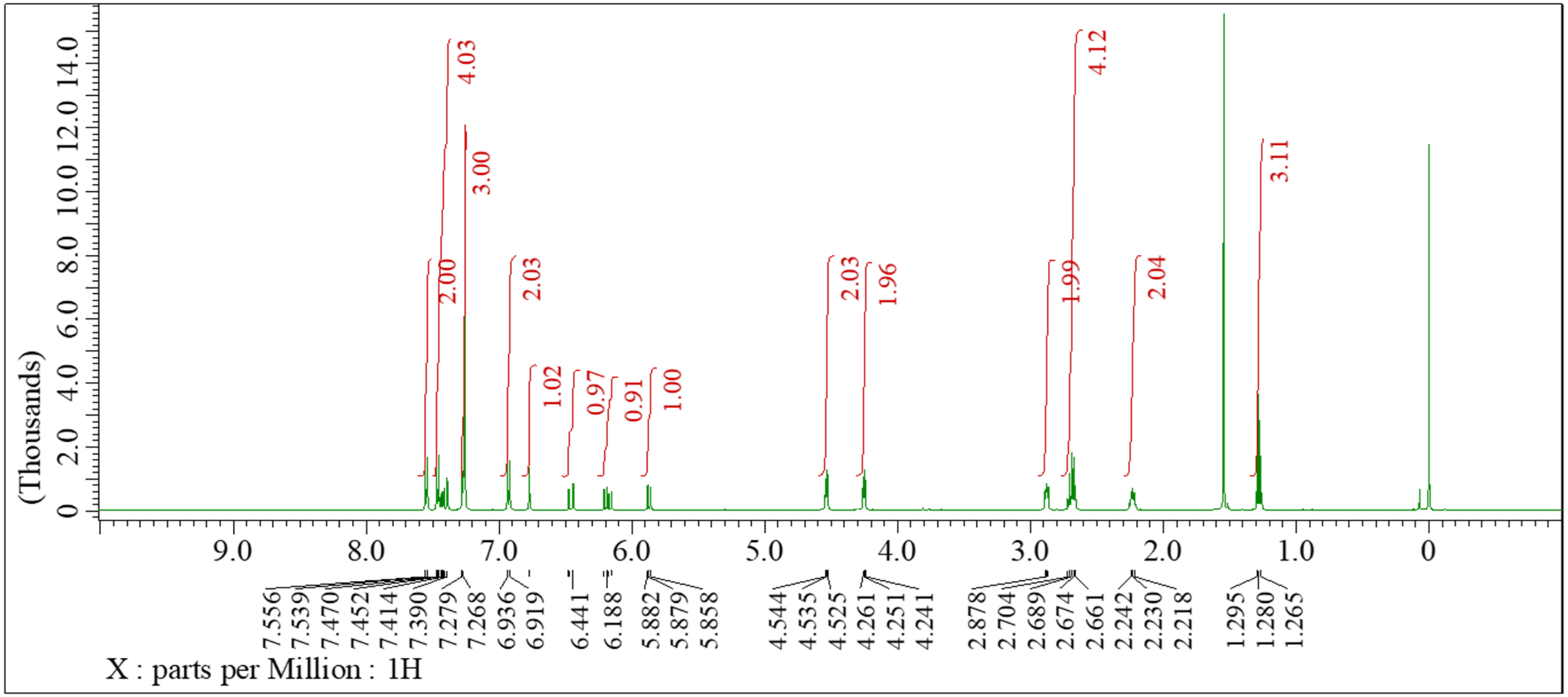

Figure S59. ^{1}H-NMR spectra of **2-(4-(3-(4-ethylphenyl)-6,7-dihydro-5*H*-benzo[7]annulen-8-yl)phenoxy)ethyl acrylate (M1)** (500Hz, $CDCl_3$).

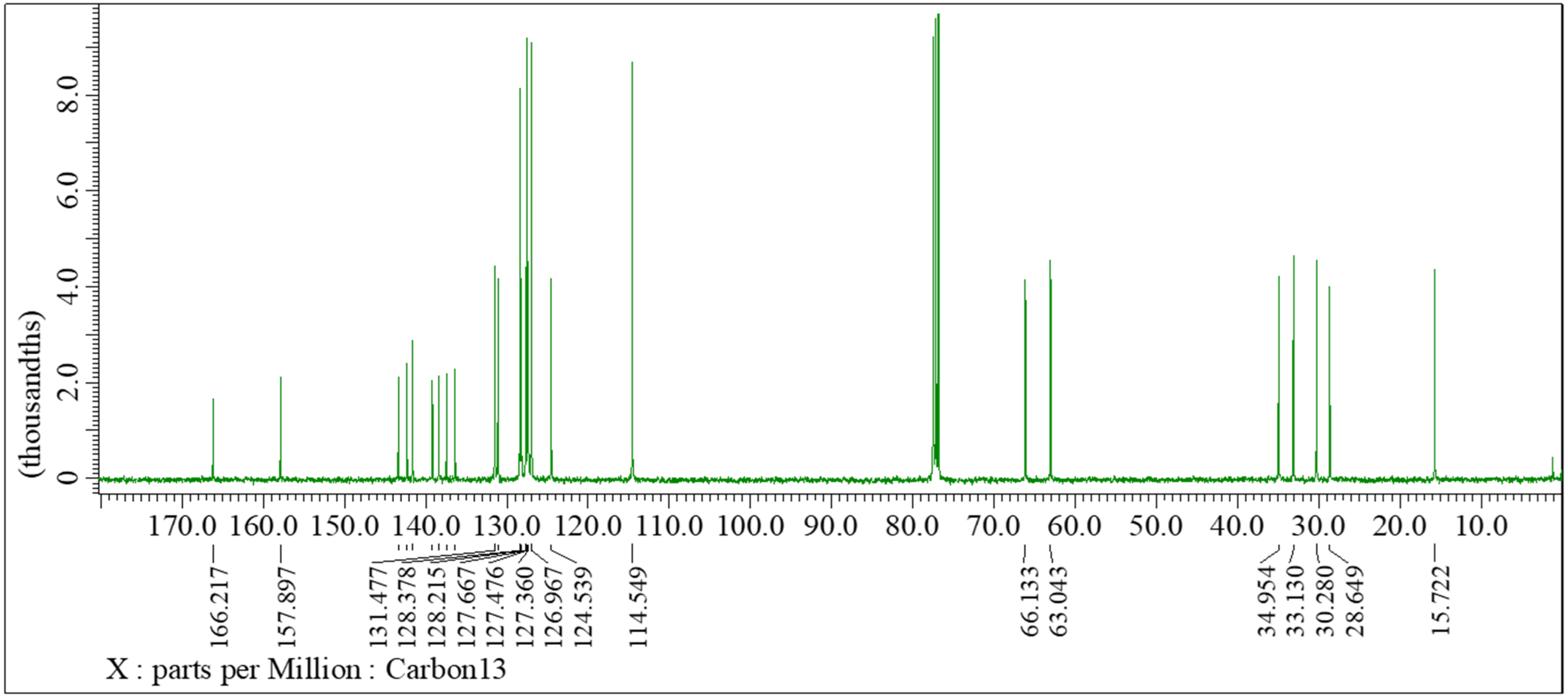

Figure S60. ^{13}C-NMR spectra of **M1** (100Hz, $CDCl_3$).

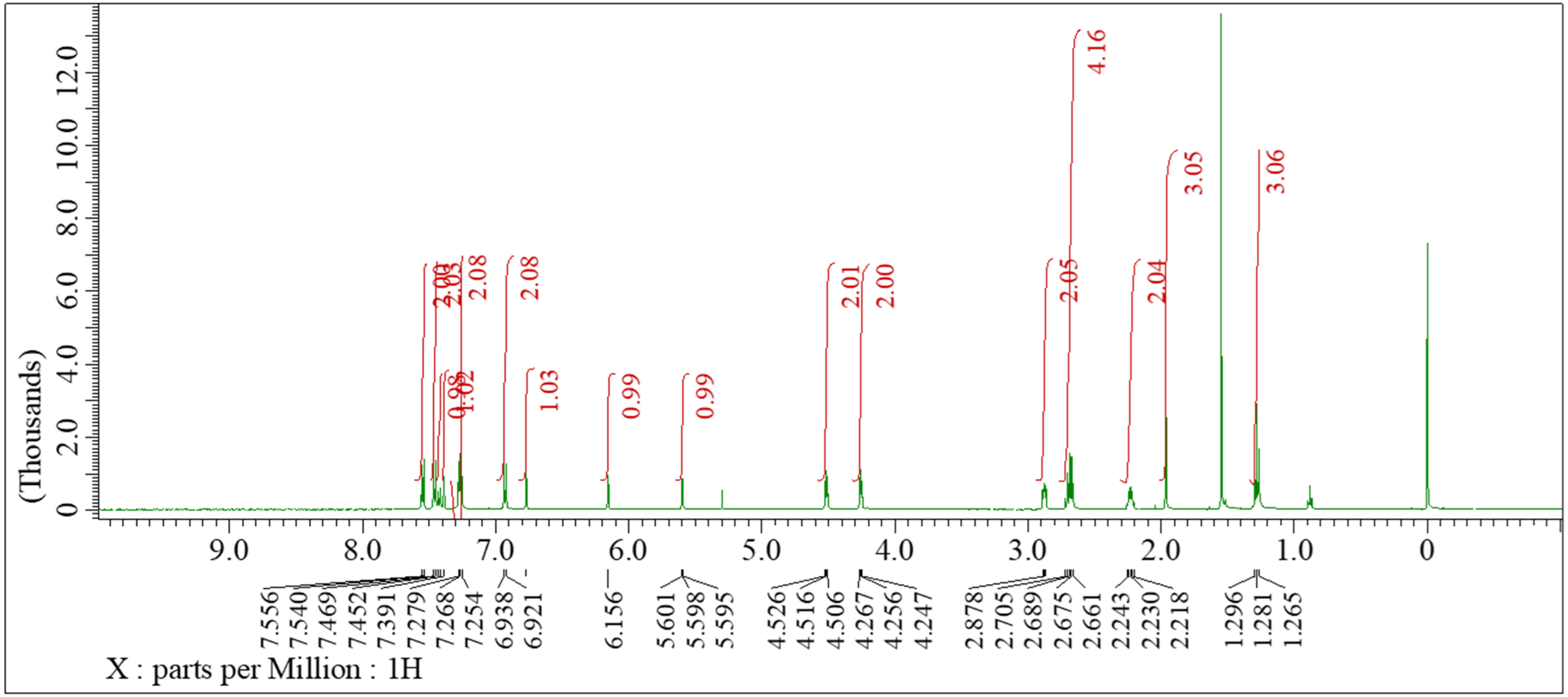

Figure S61. ^{1}H-NMR spectra of **2-(4-(3-(4-ethylphenyl)-6,7-dihydro-5*H*-benzo[7]annulen-8-yl)phenoxy)ethyl methacrylate (M2)** (500Hz, $CDCl_3$).

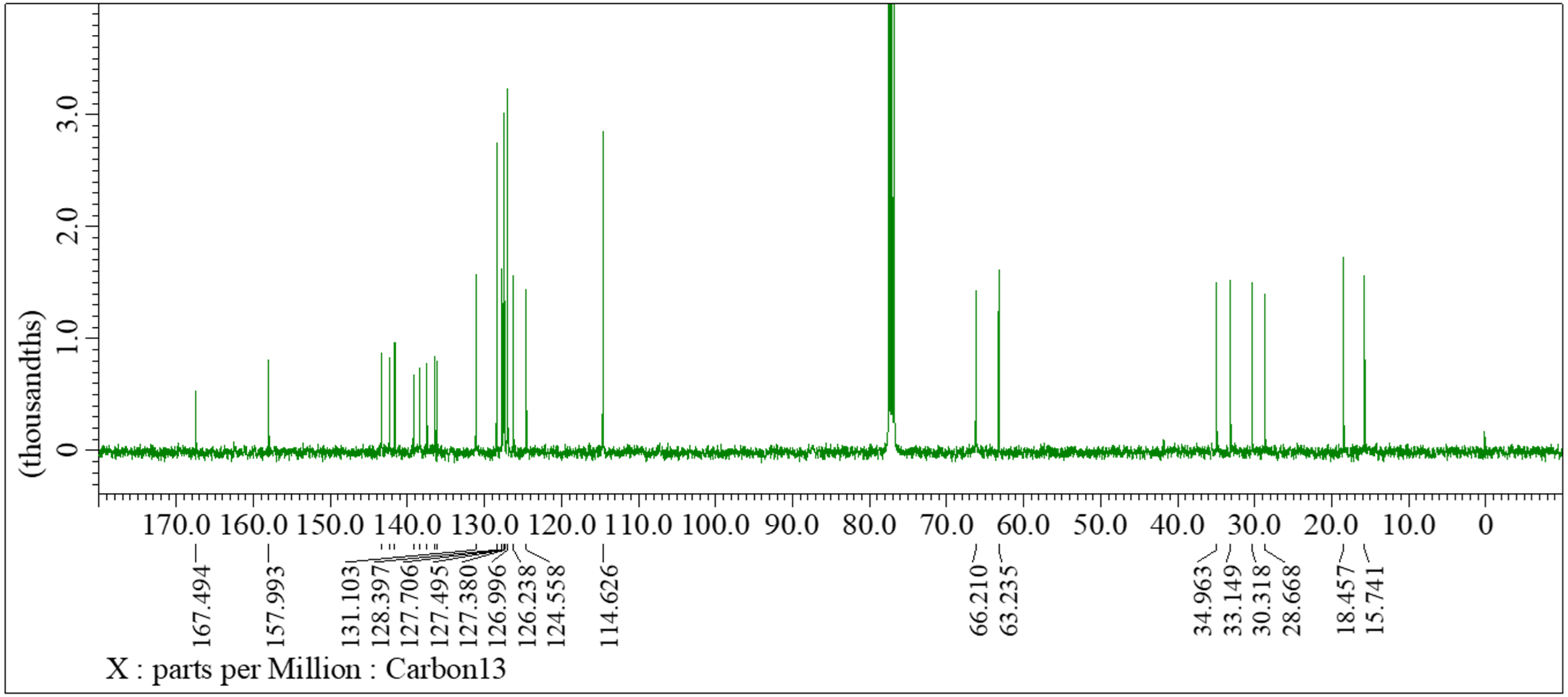

Figure S62. ^{13}C-NMR spectra of **M2** (100Hz, $CDCl_3$).

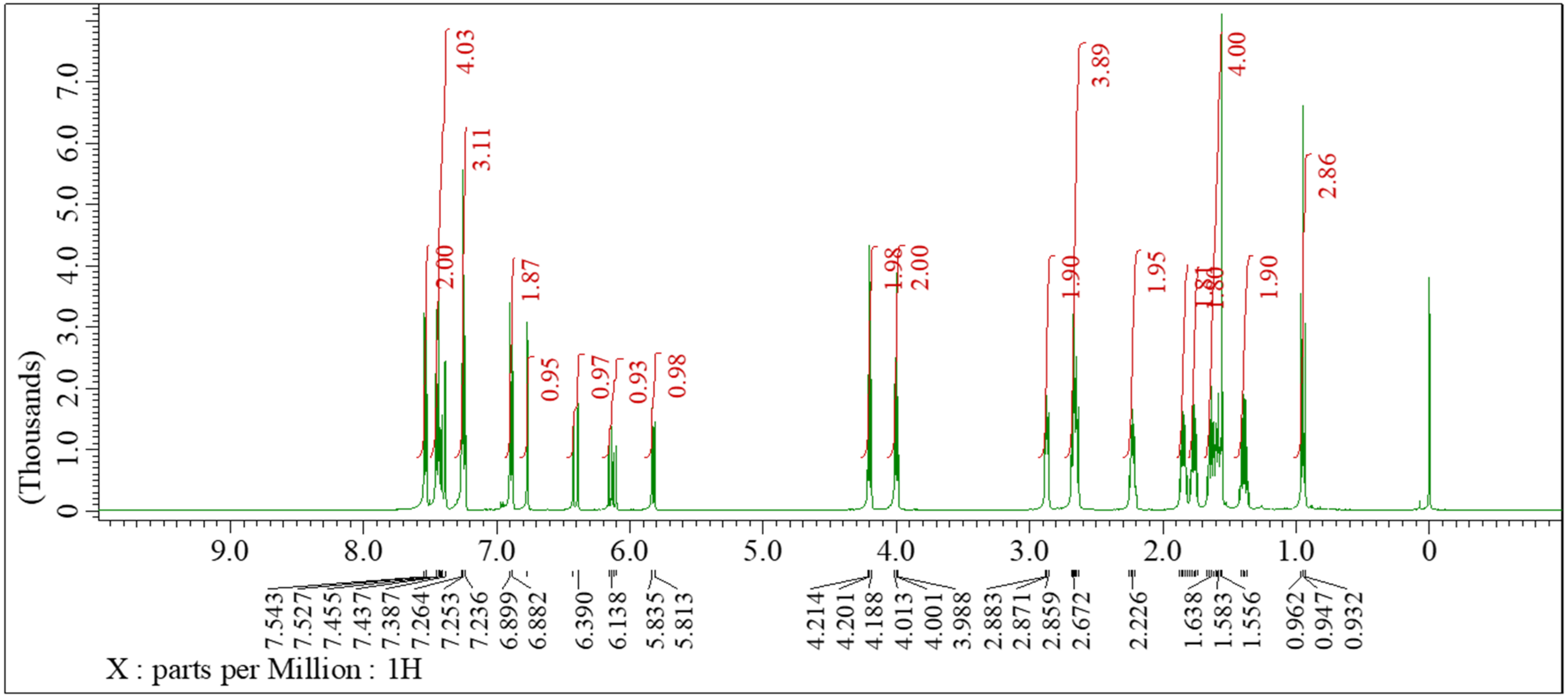

Figure S63. ^{1}H-NMR spectra of **5-(4-(3-(4-butylphenyl)-6,7-dihydro-5*H*-benzo[7]annulen-8-yl)phenoxy)pentyl acrylate (M3)** (500Hz, $CDCl_3$).

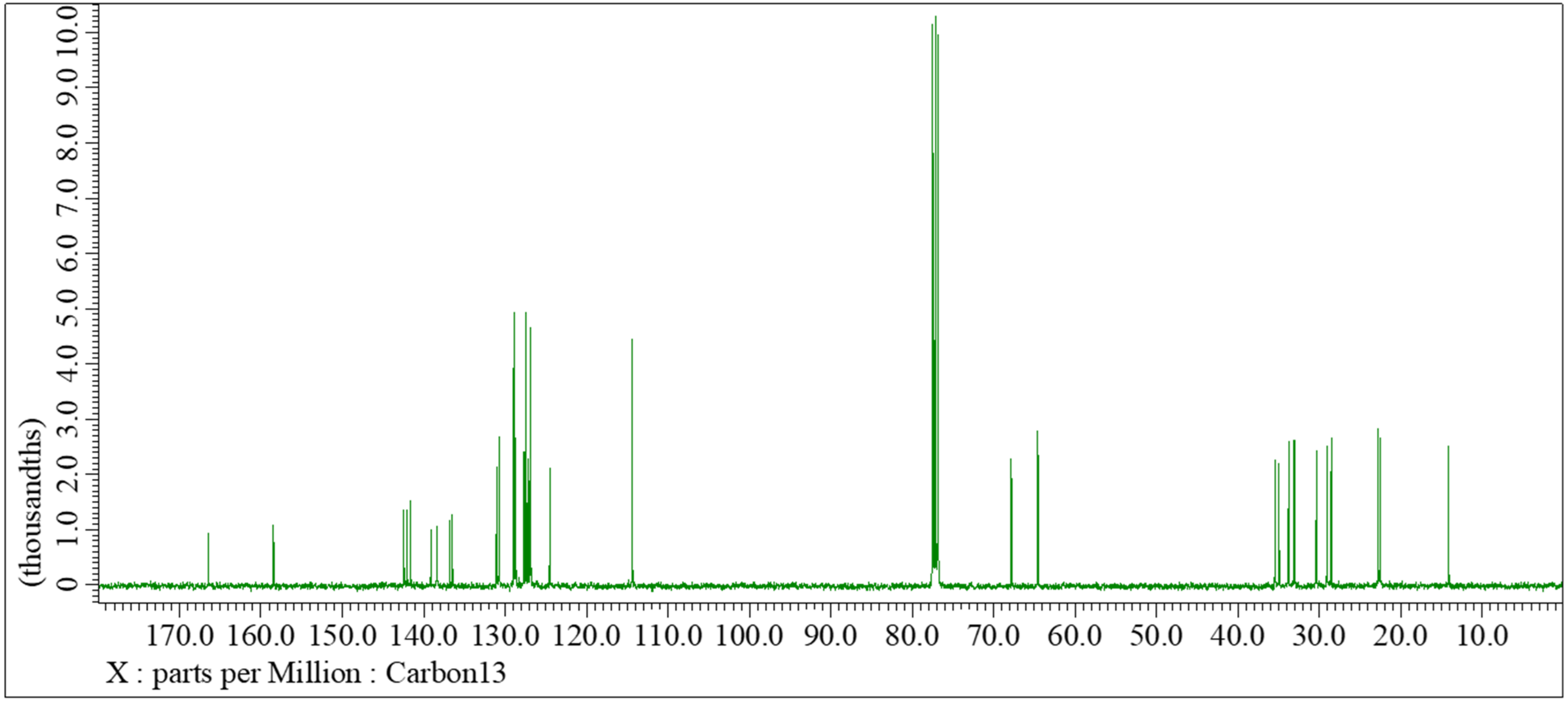

Figure S64. ^{13}C-NMR spectra of **M3** (100Hz, $CDCl_3$).

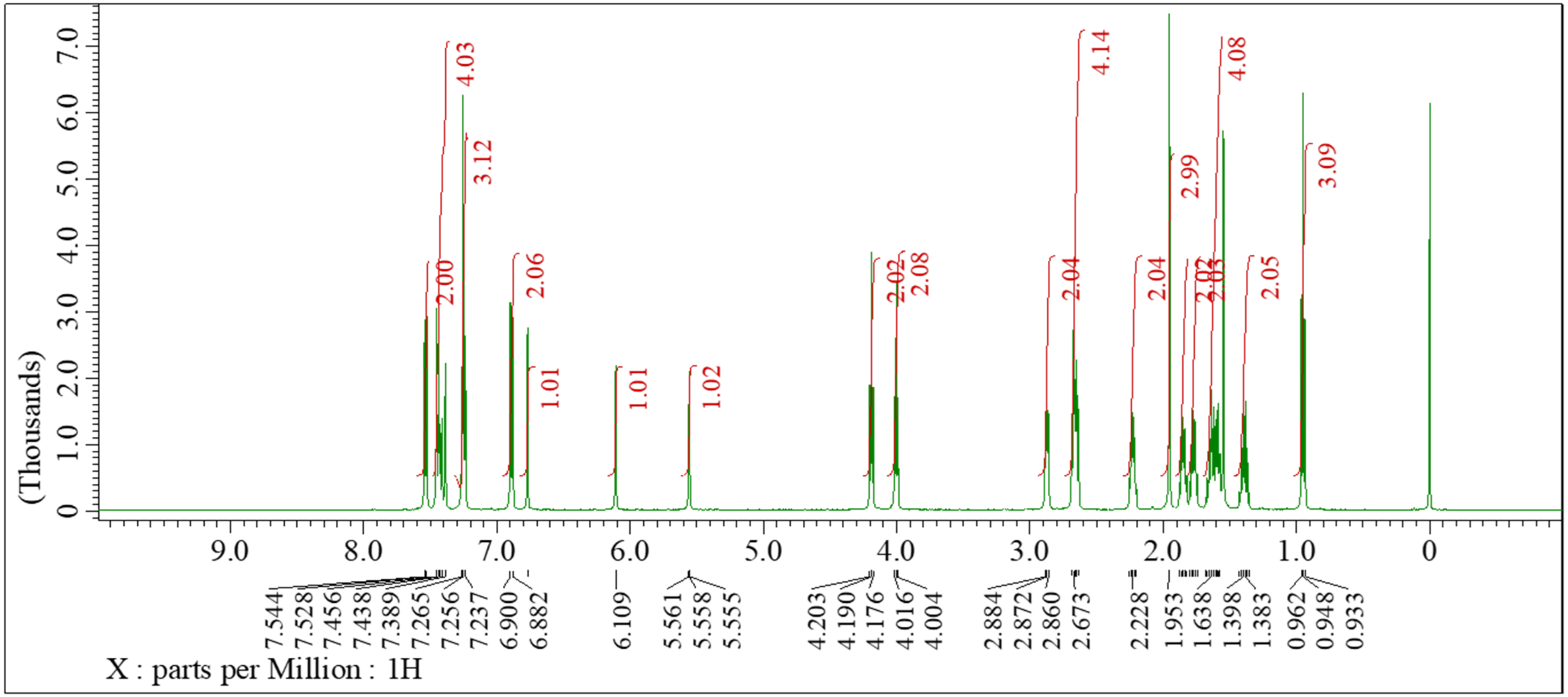

Figure S65. ^{1}H-NMR spectra of **5-(4-(3-(4-butylphenyl)-6,7-dihydro-5*H*-benzo[7]annulen-8-yl)phenoxy)pentyl methacrylate (M4)** (500Hz, $CDCl_3$).

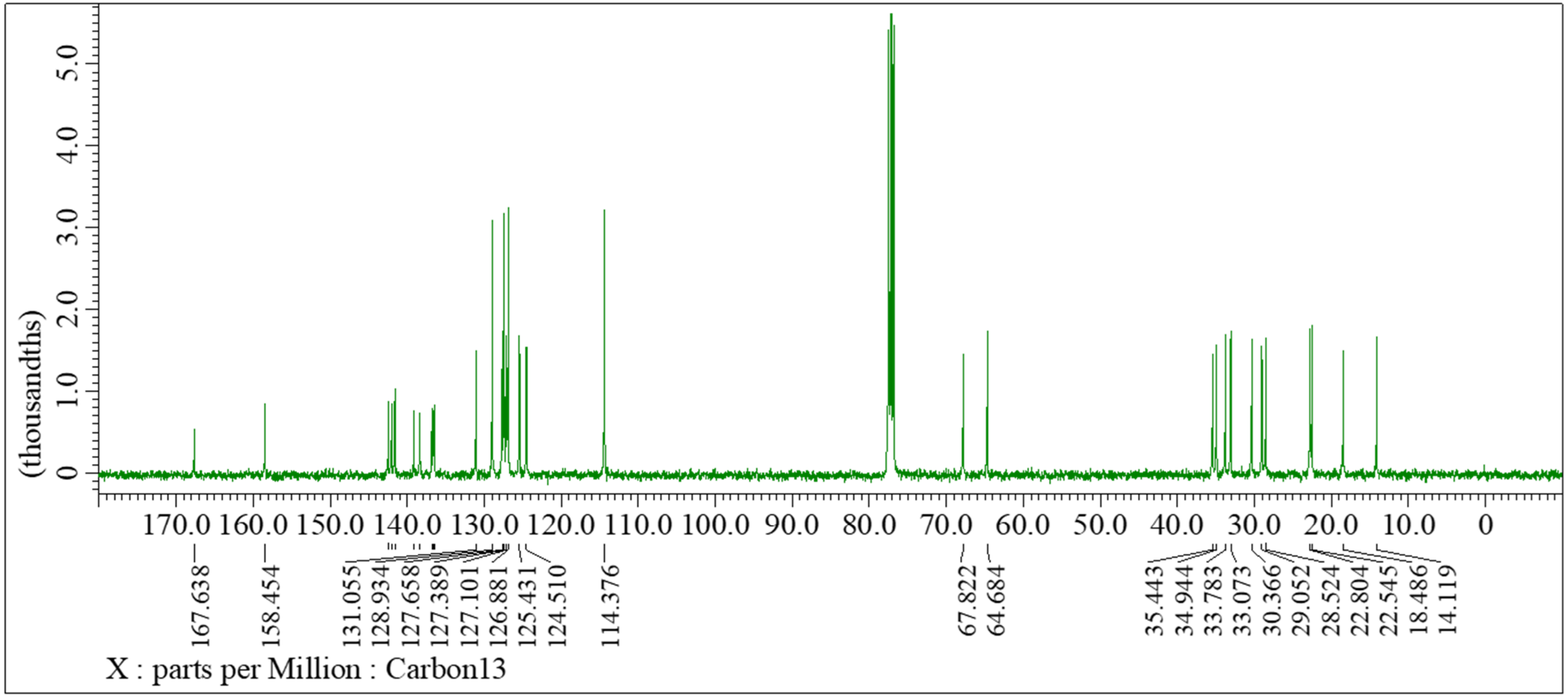

Figure S66. ^{13}C-NMR spectra of **M4** (100Hz, $CDCl_3$).

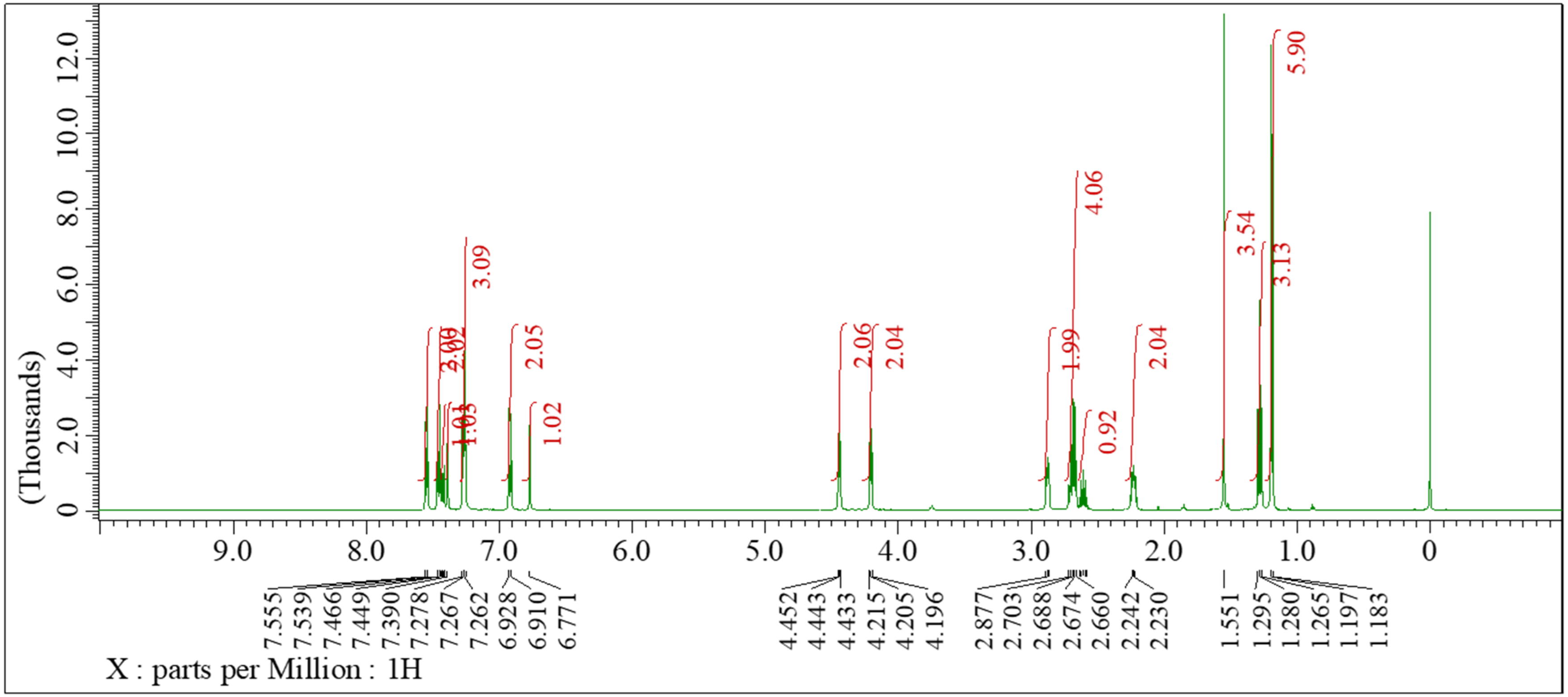

Figure S67. ^{1}H-NMR spectra of **2-(4-(3-(4-ethylphenyl)-6,7-dihydro-5*H*-benzo[7]annulen-8-yl)phenoxy)ethyl isobutyrate (M5)** (500Hz, $CDCl_3$).

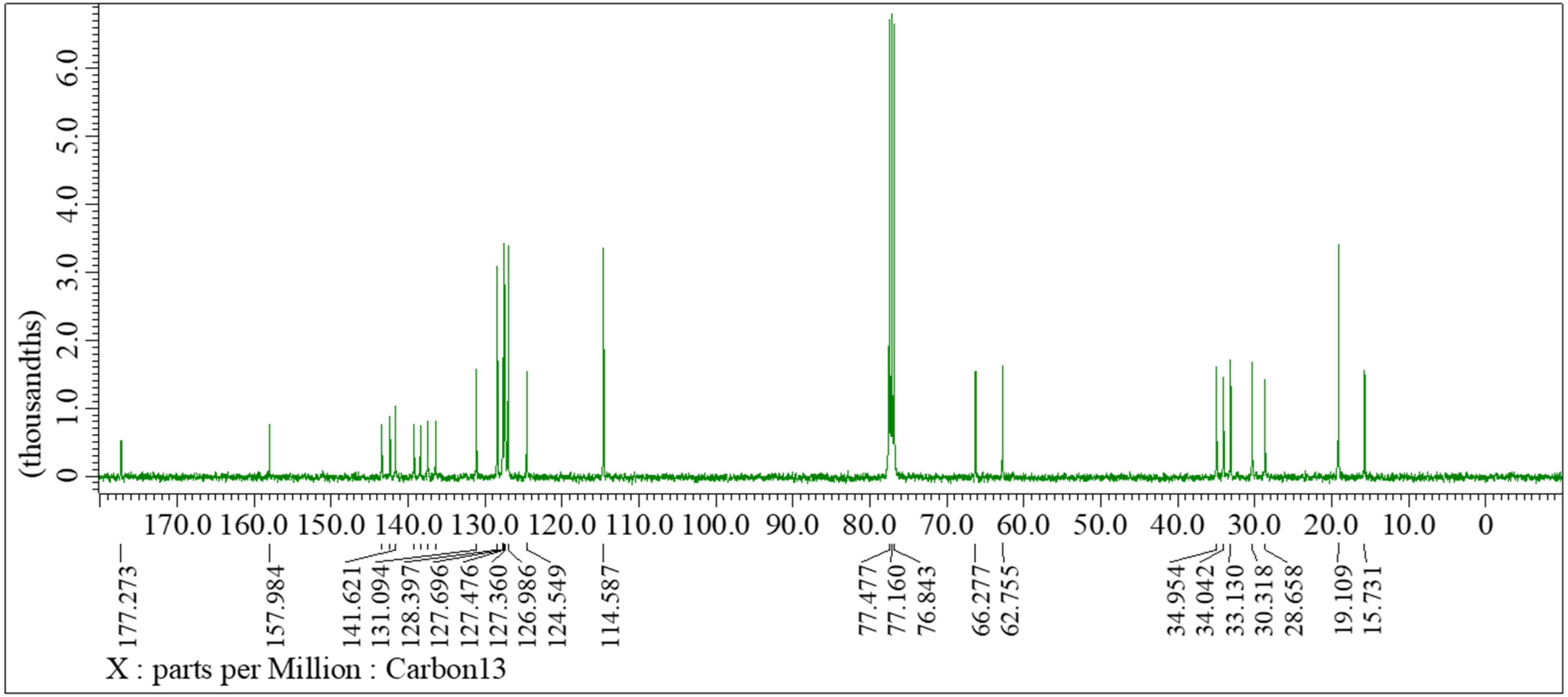

Figure S68 ^{13}C-NMR spectra of **M5** (100Hz, $CDCl_3$).

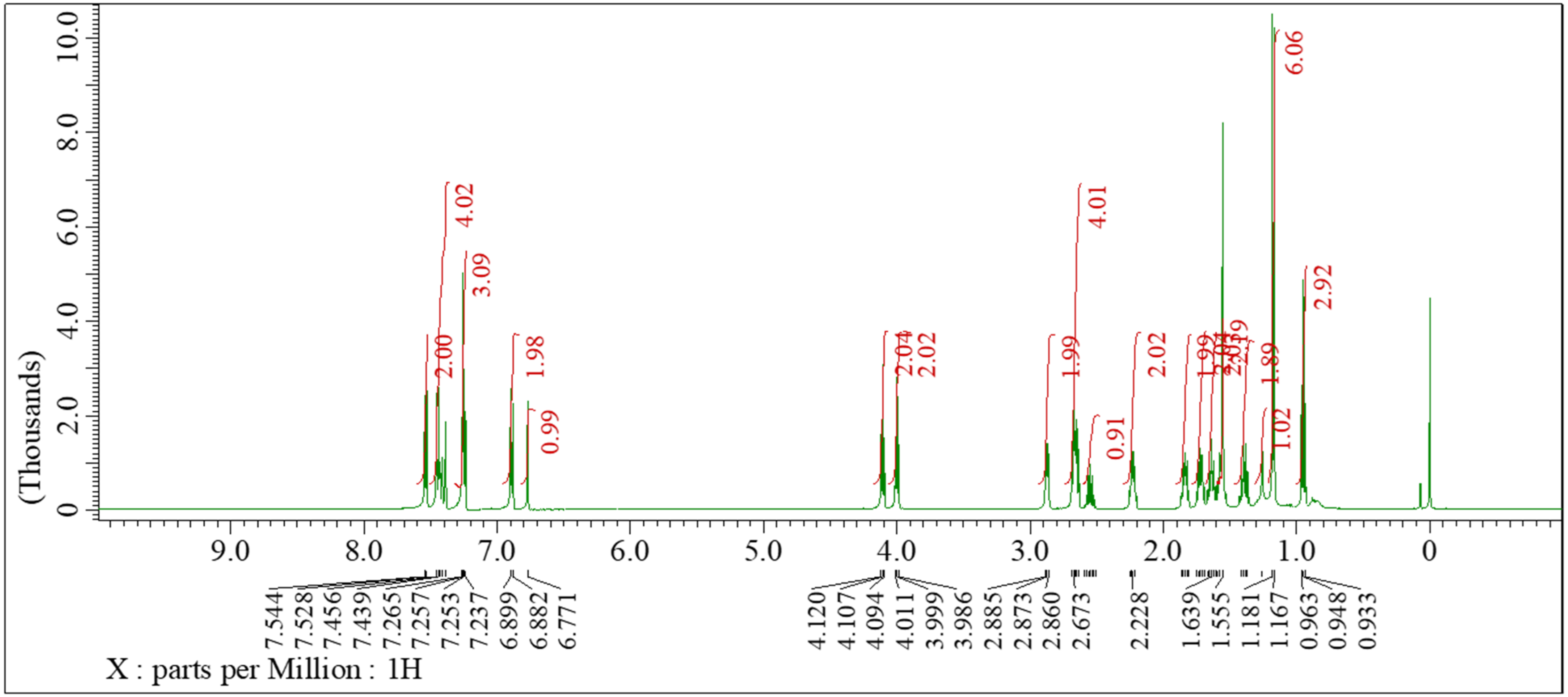

Figure S69. ^{1}H-NMR spectra of **5-(4-(3-(4-butylphenyl)-6,7-dihydro-5*H*-benzo[7]annulen-8-yl)phenoxy)pentyl isobutyrate (M6)** (500Hz, $CDCl_3$).

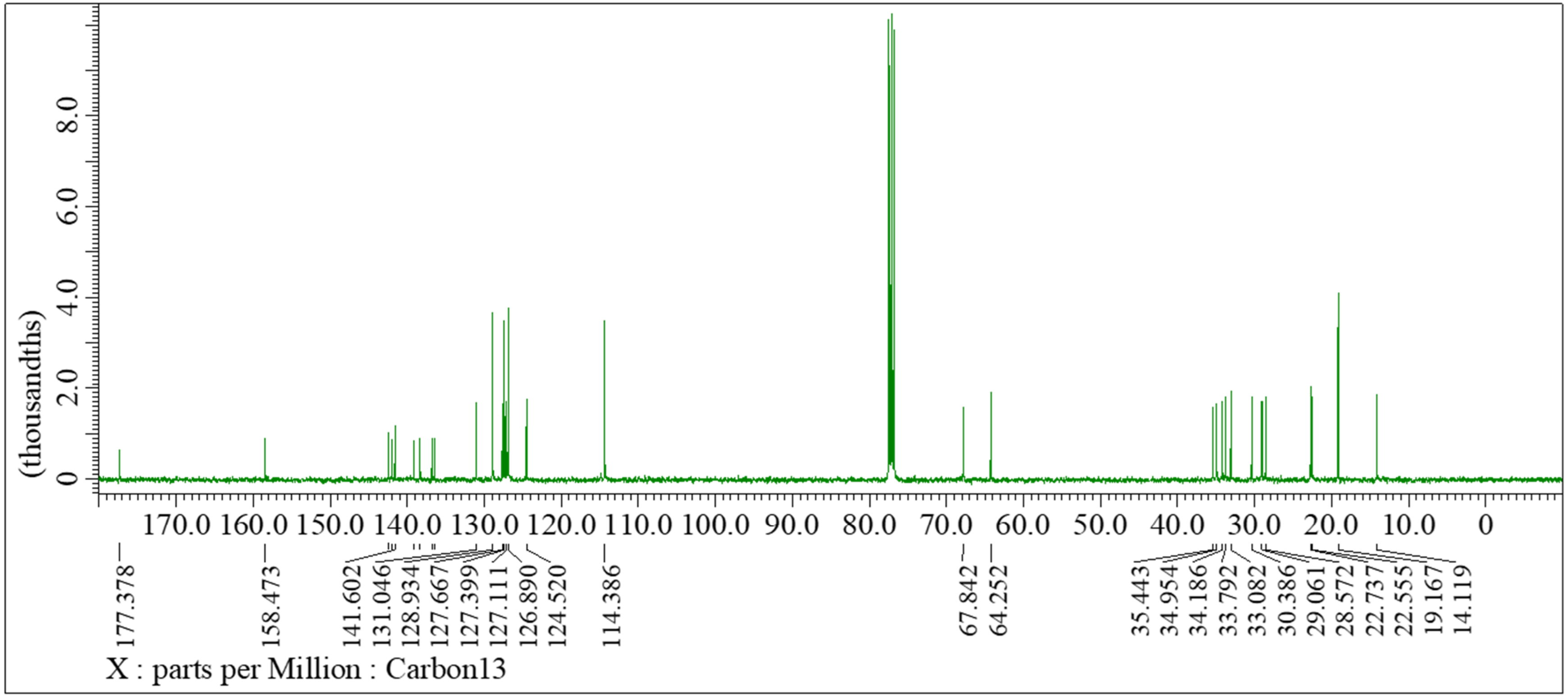

Figure S70. ^{13}C-NMR spectra of **M6** (100Hz, $CDCl_3$).

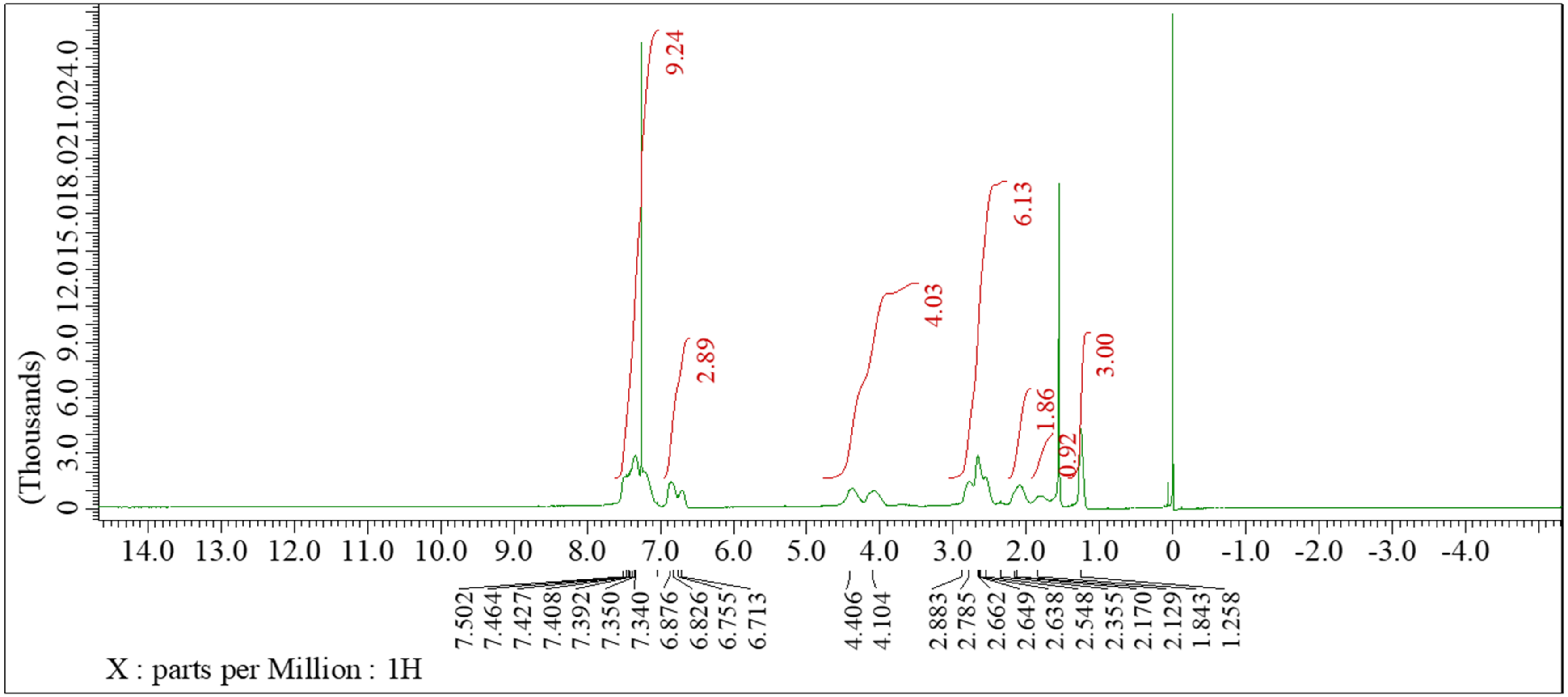

Figure S71. ^{1}H-NMR spectra of **Poly[2-(4-(3-(4-ethylphenyl)-6,7-dihydro-5*H*-benzo[7]annulen-8-yl)phenoxy)ethyl] acrylate (P1)** (500Hz, $CDCl_3$).

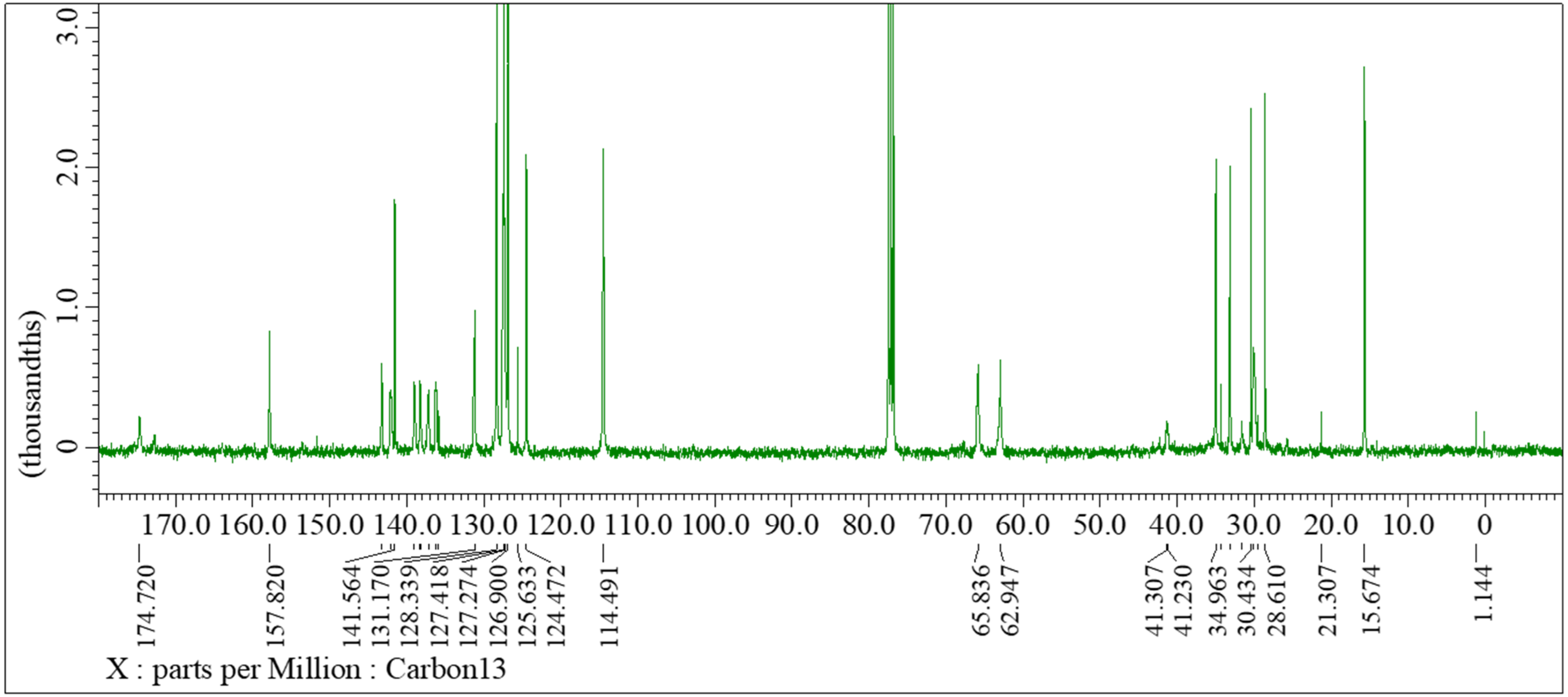

Figure S72. ^{13}C-NMR spectra of **P1** (100Hz, $CDCl_3$).

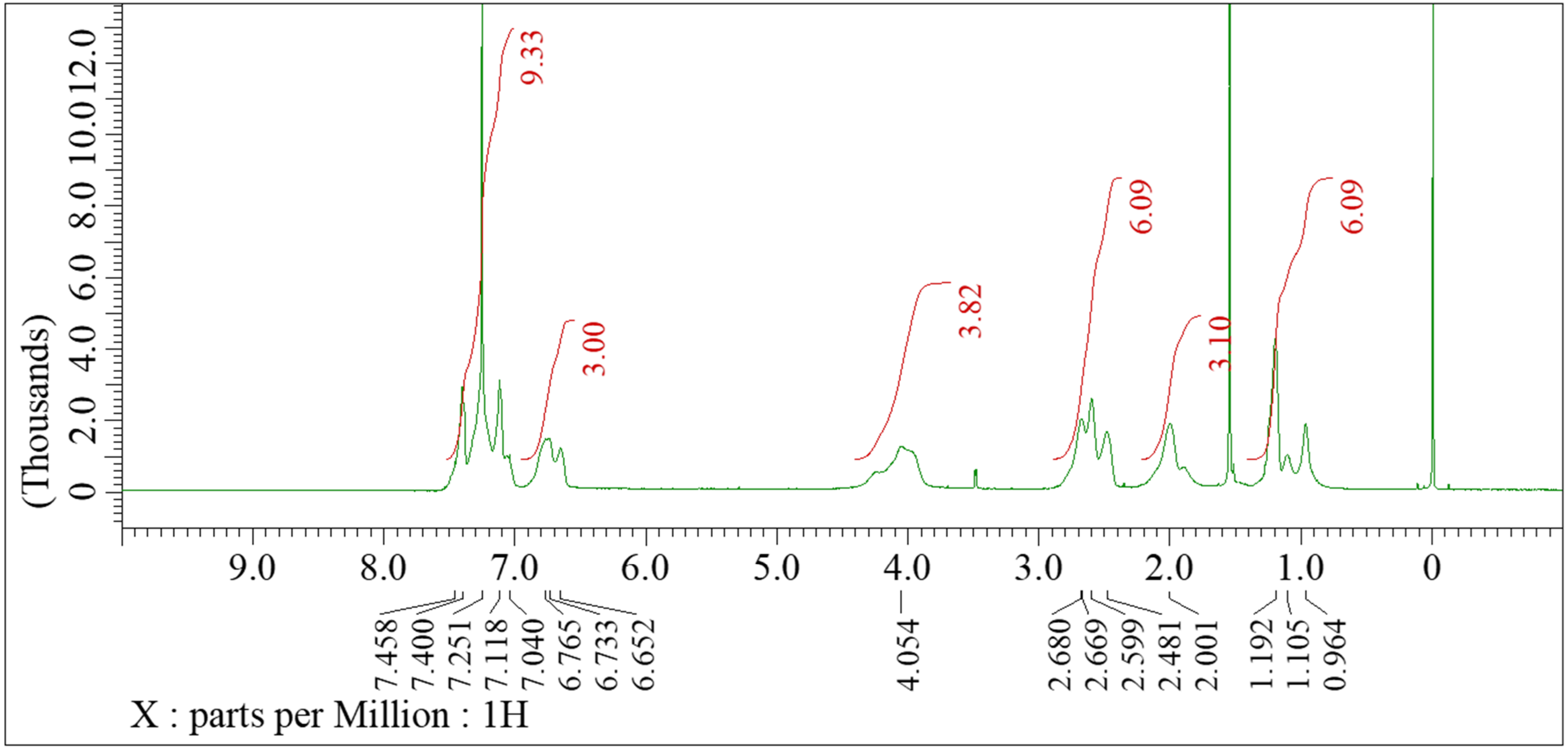

Figure S73. ^{1}H-NMR spectra of **Poly[2-(4-(3-(4-ethylphenyl)-6,7-dihydro-5*H*-benzo[7]annulen-8-yl)phenoxy)ethyl] methacrylate (P2)** (500Hz, $CDCl_3$).

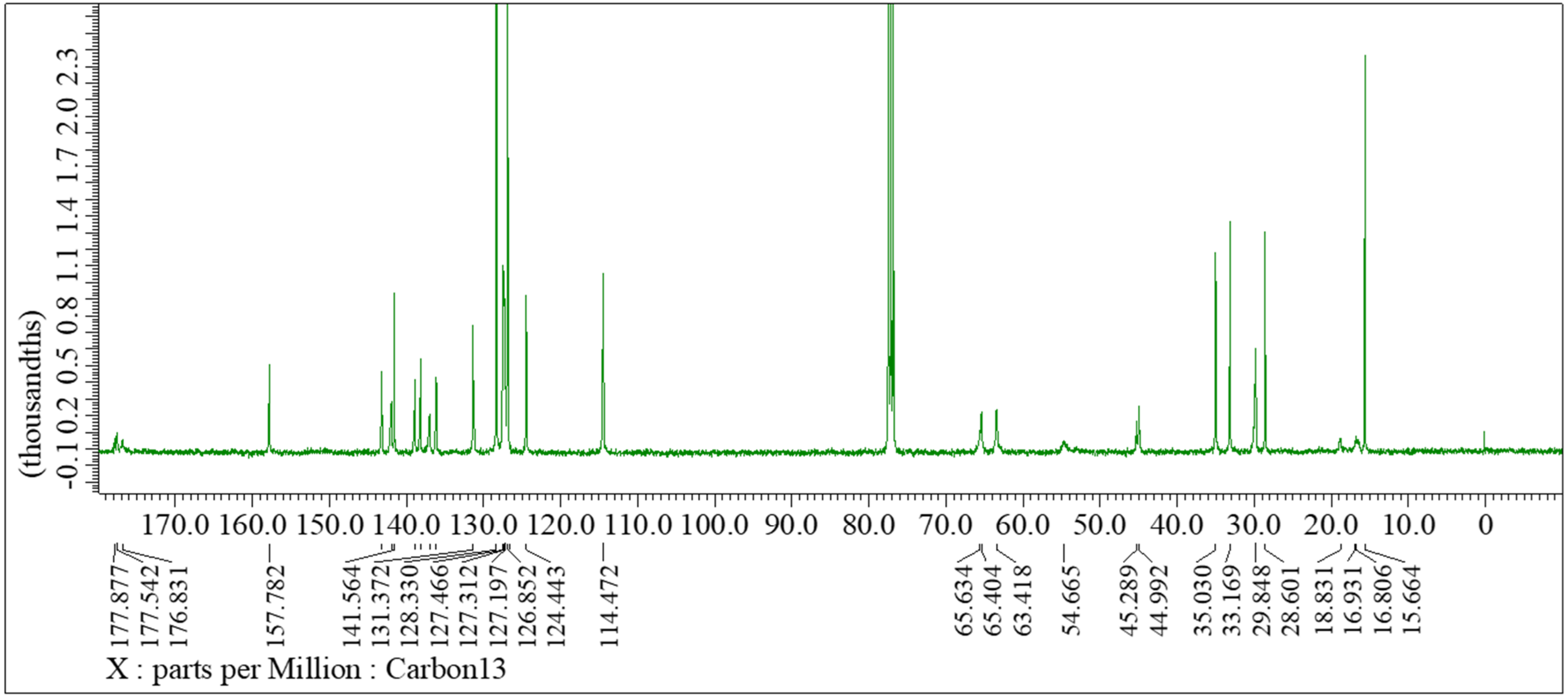

Figure S74. ^{13}C-NMR spectra of **P2** (100Hz, $CDCl_3$).

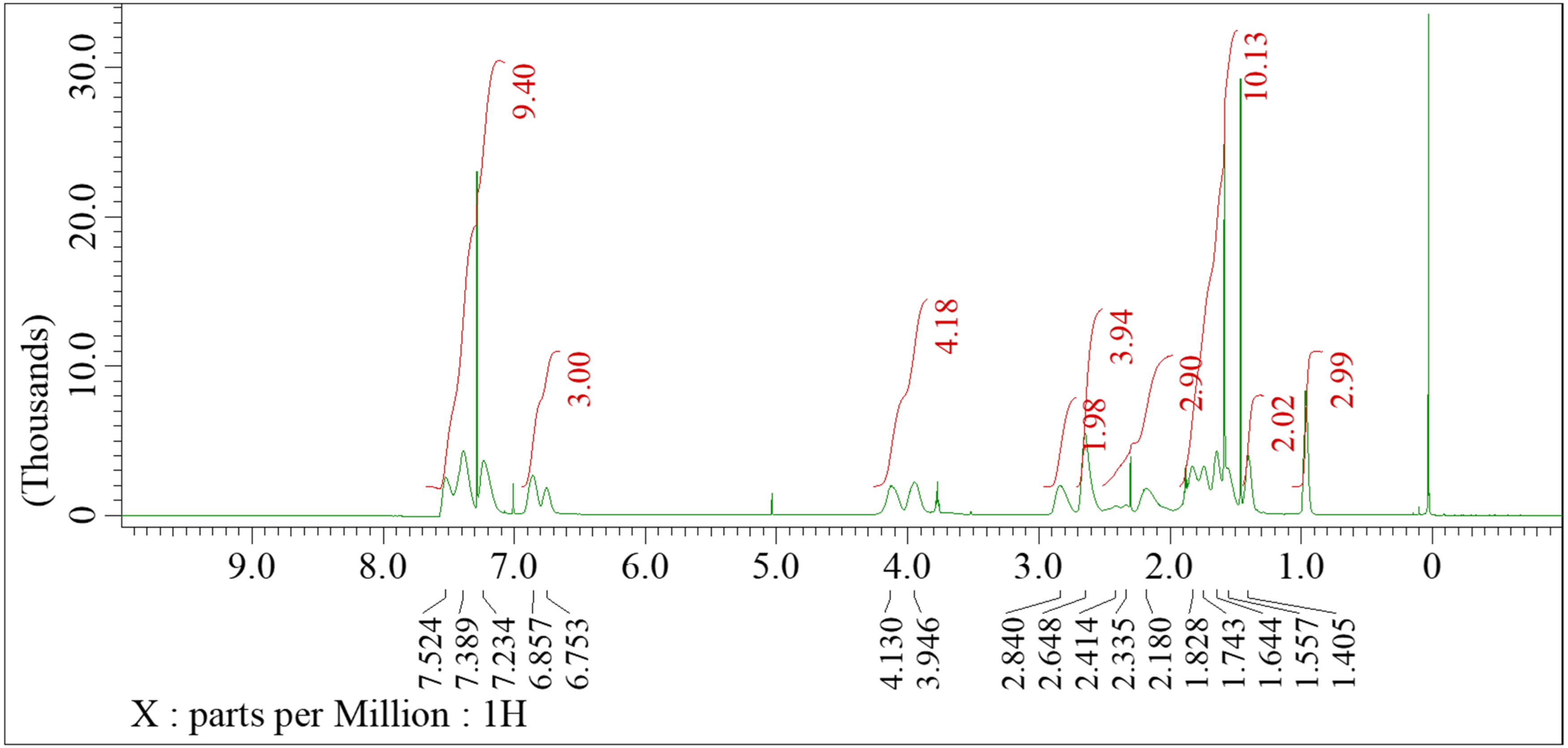

Figure S75. ^{1}H-NMR spectra of **Poly[5-(4-(3-(4-butylphenyl)-6,7-dihydro-5*H*-benzo[7]annulen-8-yl)phenoxy)pentyl] acrylate (P3)** (500Hz, $CDCl_3$).

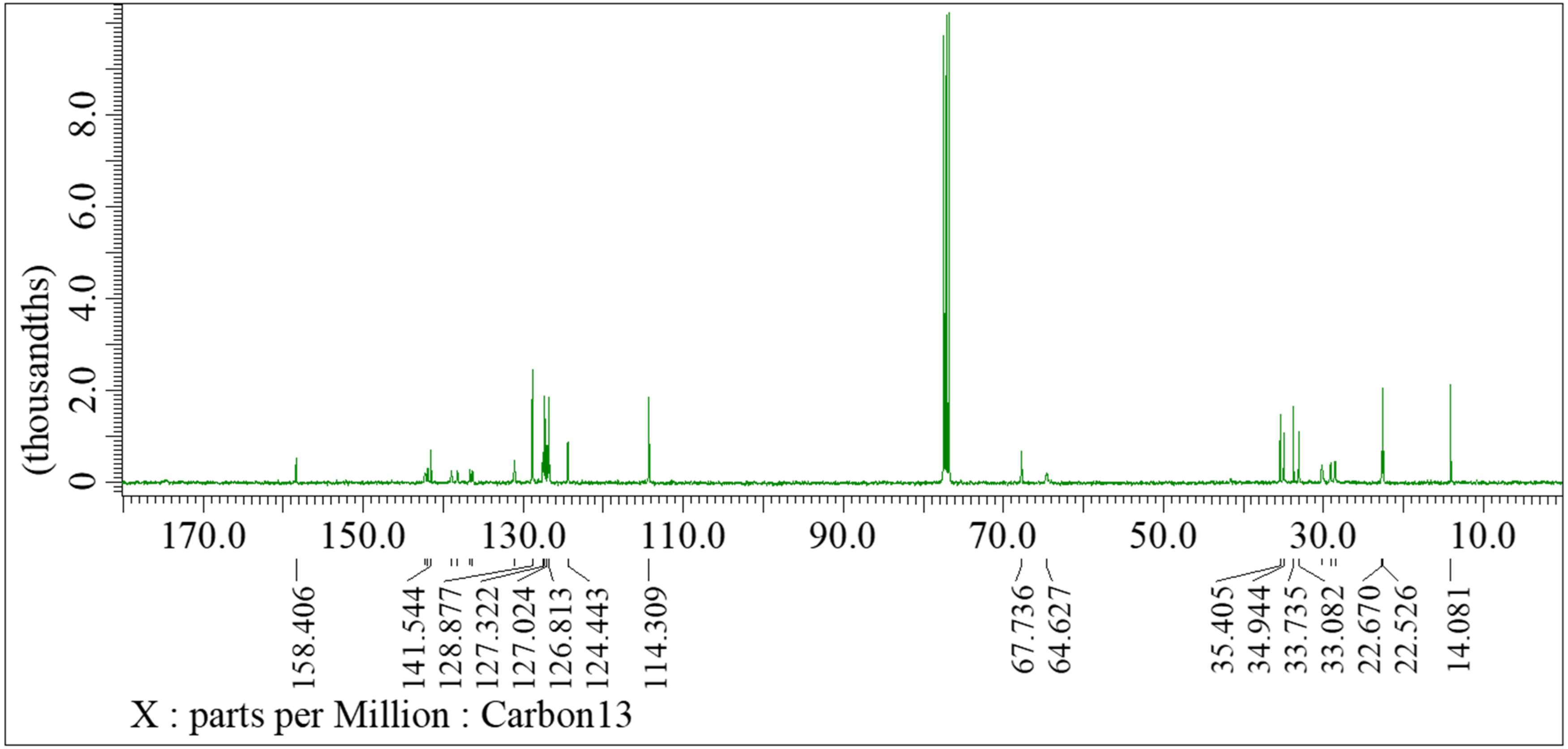

Figure S76. ^{13}C-NMR spectra of **P3** (100Hz, $CDCl_3$).

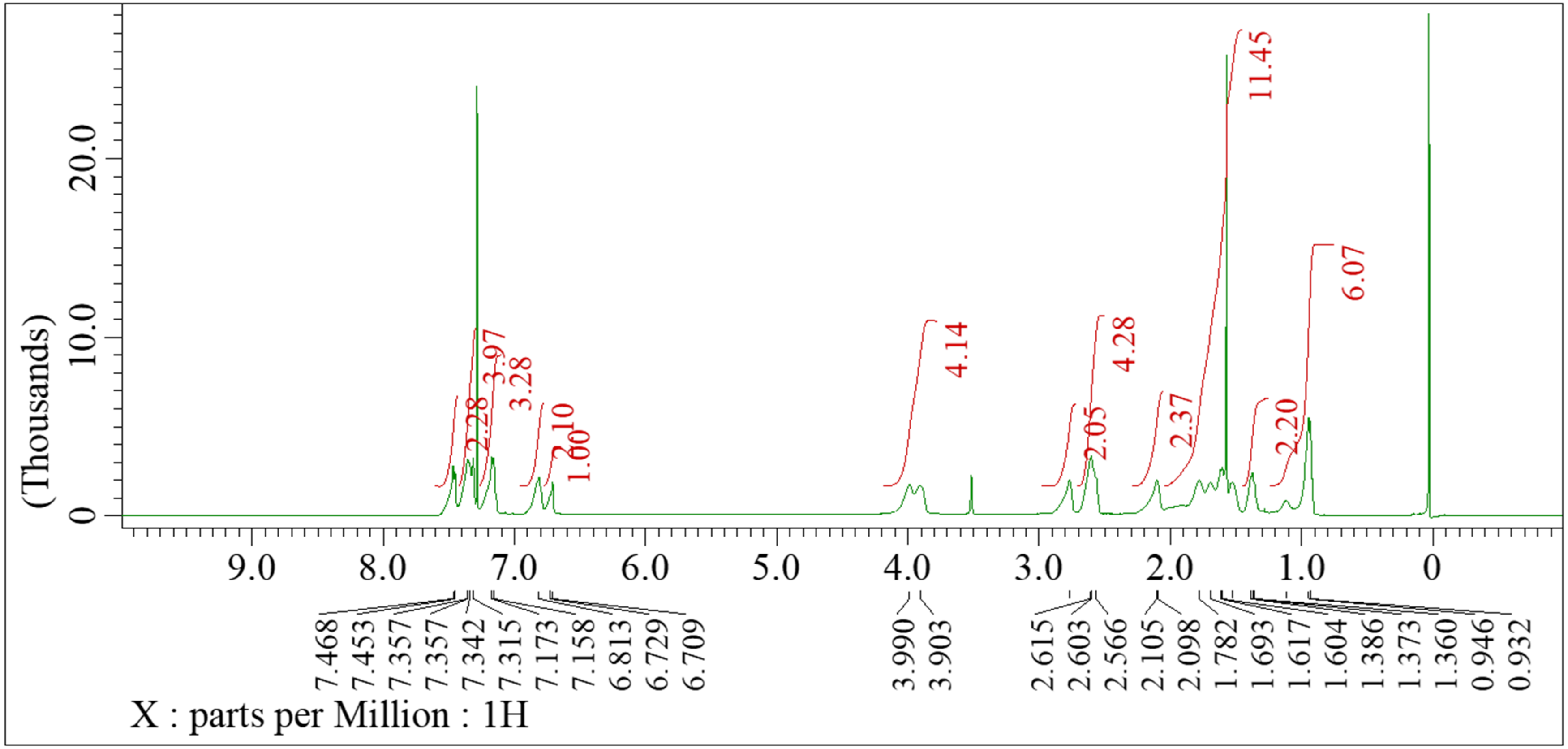

Figure S77. ^{1}H-NMR spectra of **Poly[5-(4-(3-(4-butylphenyl)-6,7-dihydro-5*H*-benzo[7]annulen-8-yl)phenoxy)pentyl] methacrylate (P4)** (500Hz, $CDCl_3$).

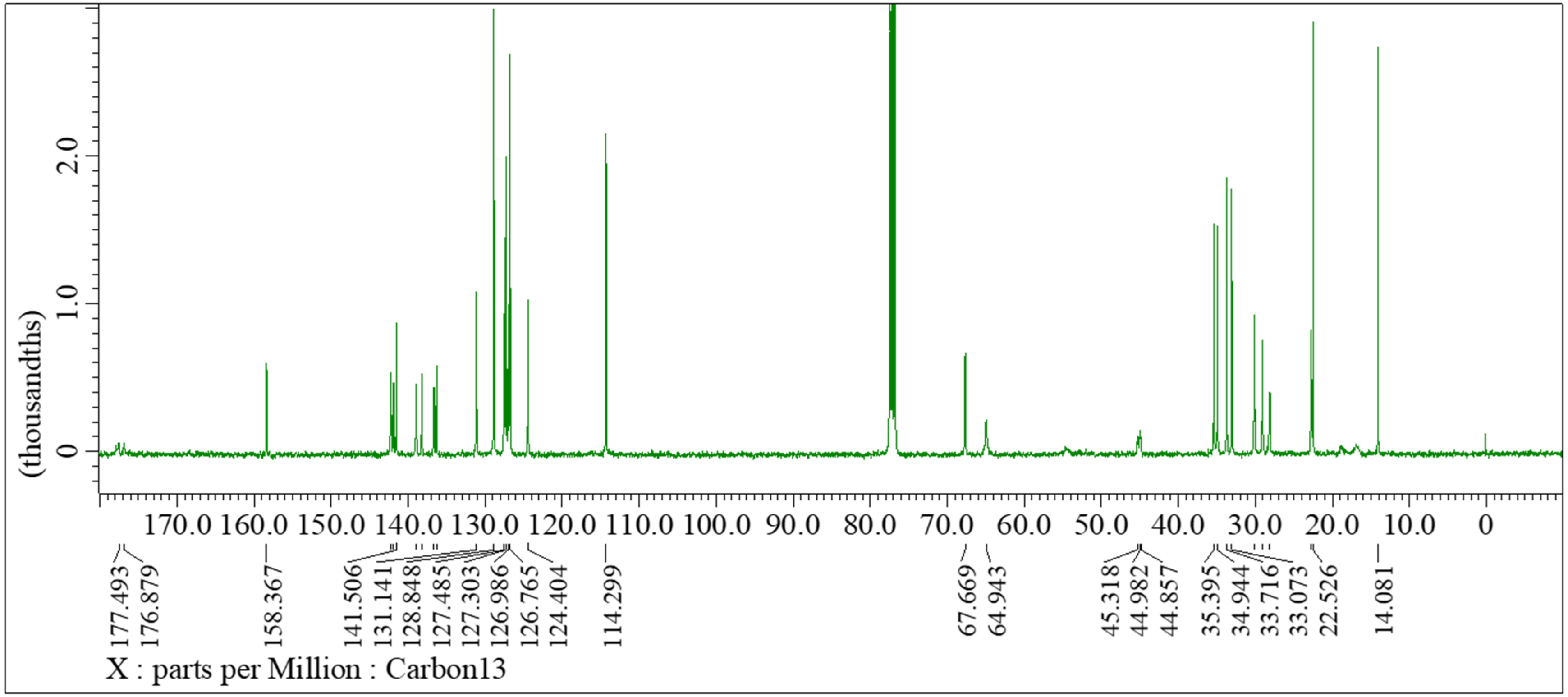

Figure S78. ^{13}C-NMR spectra of **P4** (100Hz, $CDCl_3$).

FT-IR spectra

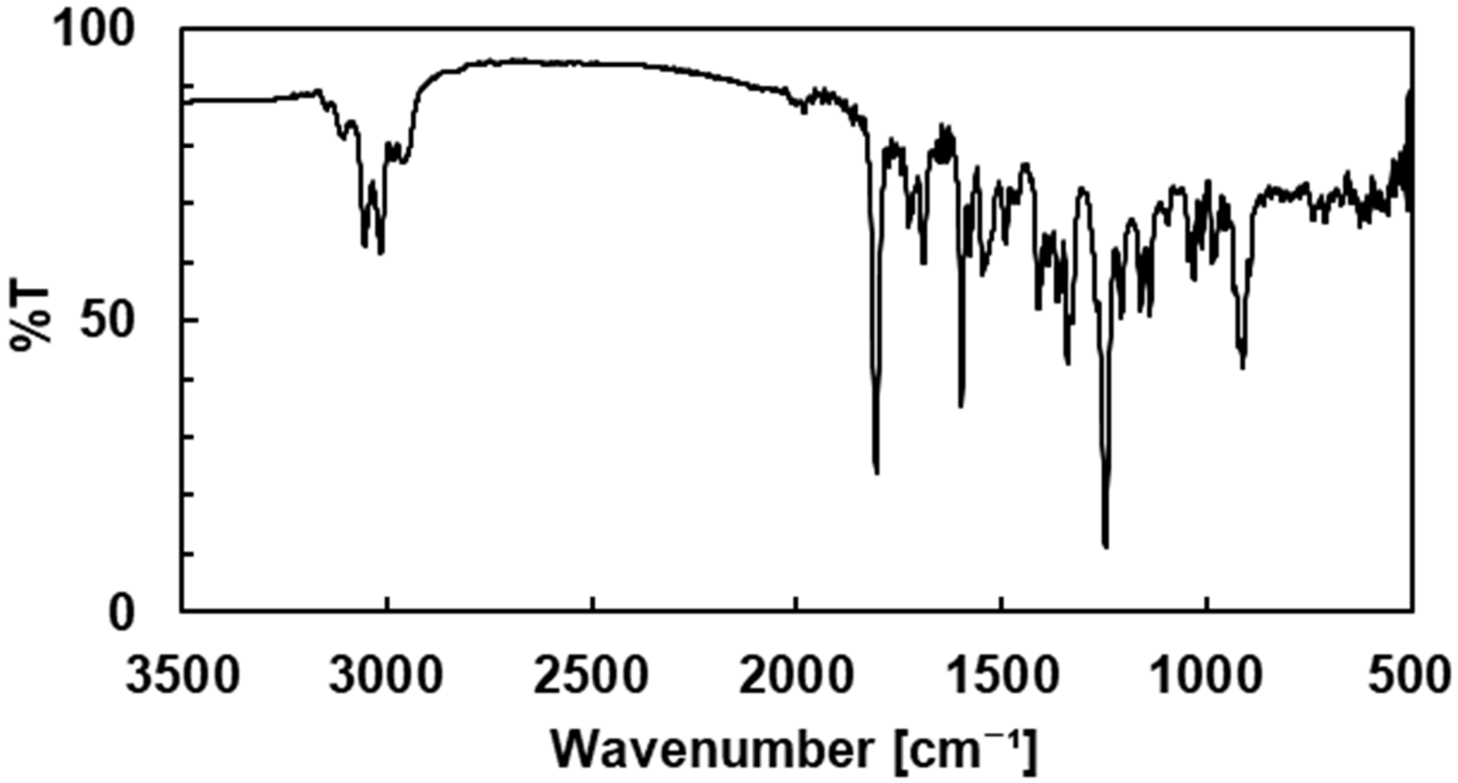

Figure S79. IR spectrum of **M2** in KBr pellets.

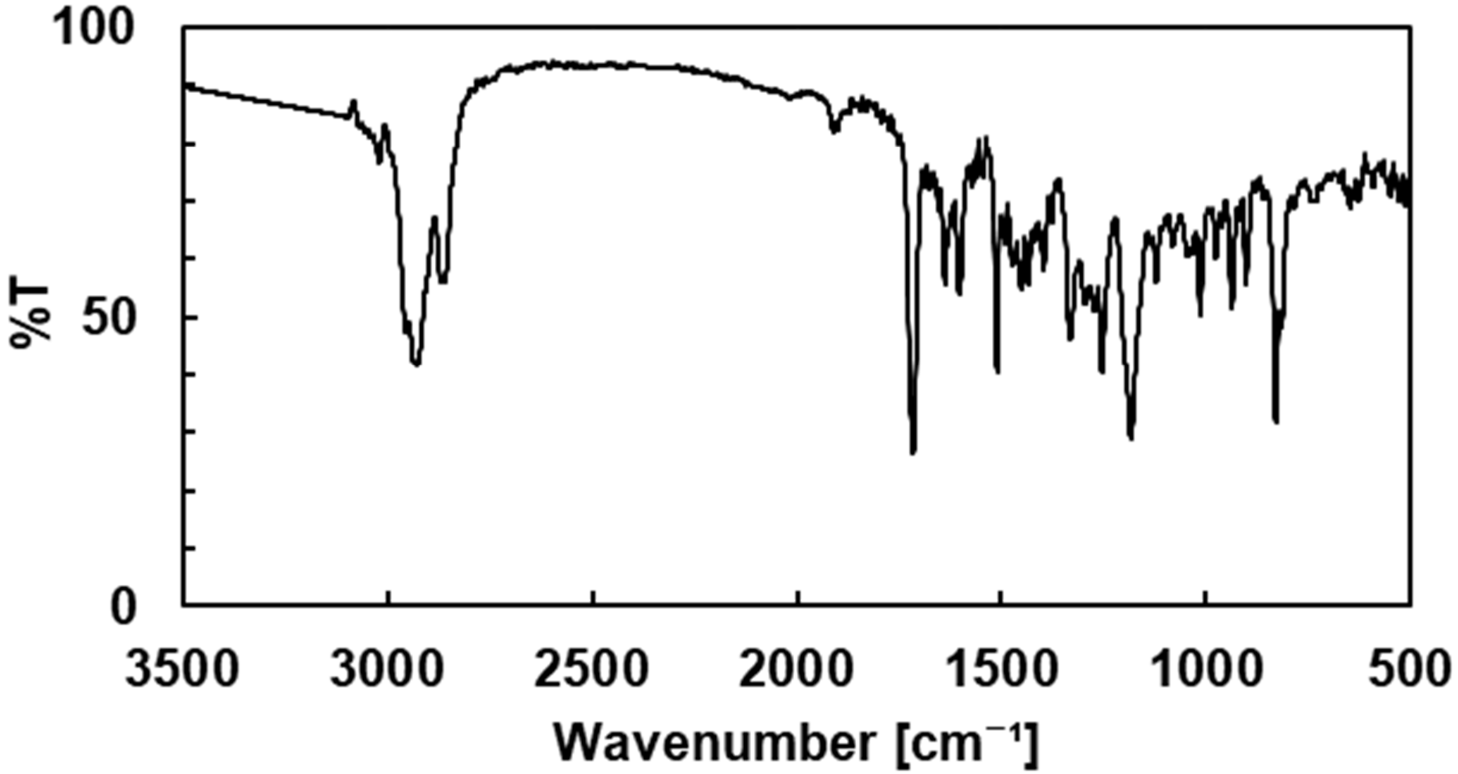

Figure S80. IR spectrum of **M4** in KBr pellets.

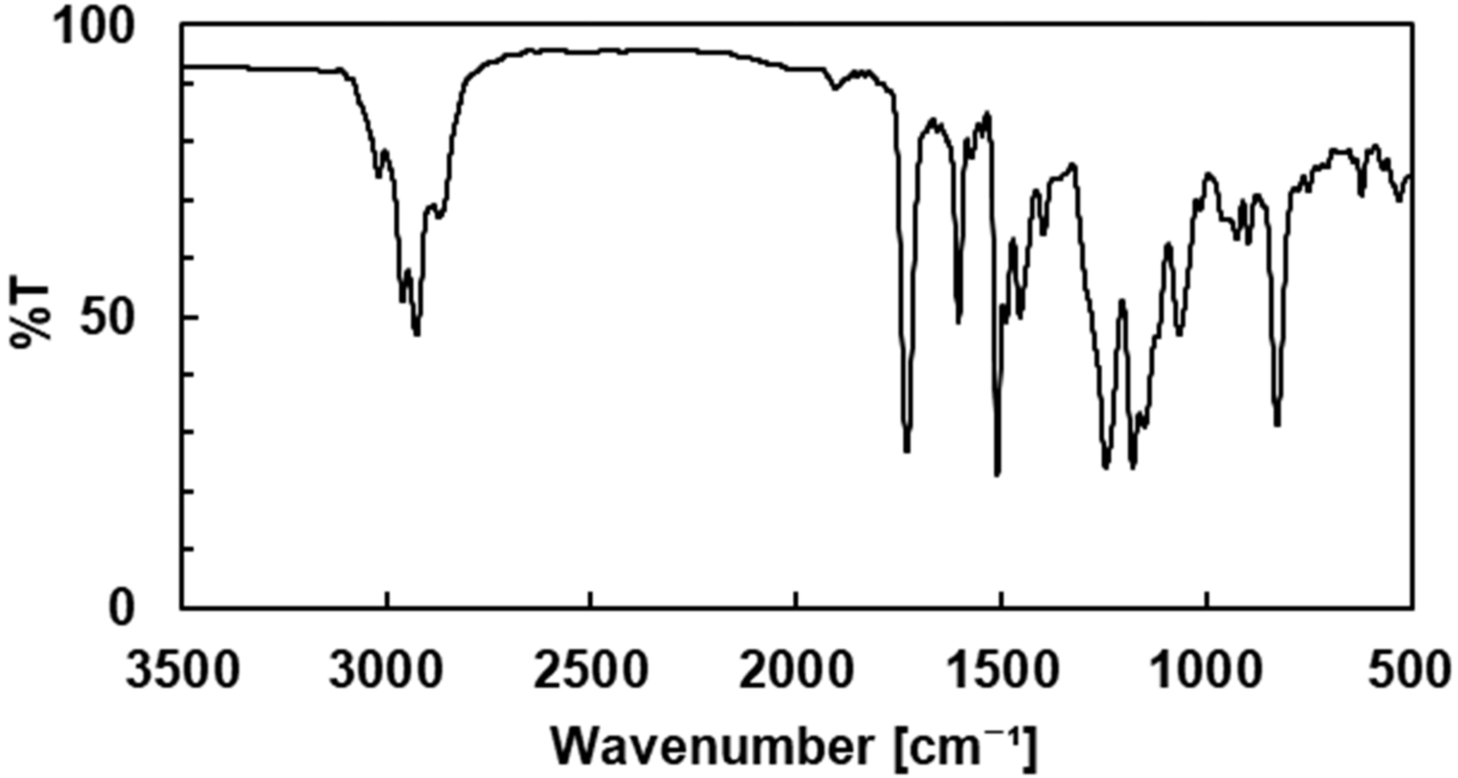

Figure S81. IR spectrum of **P2** in KBr pellets.

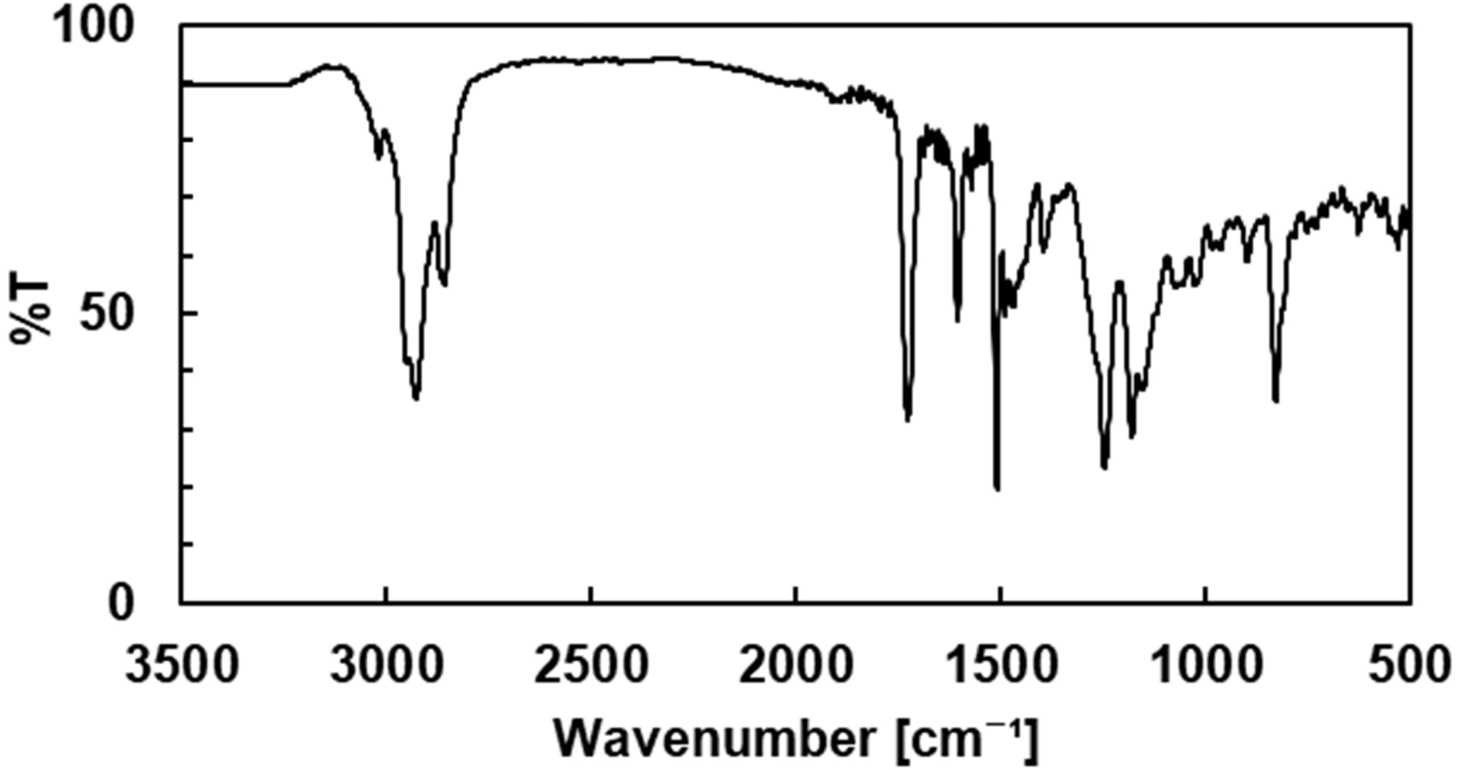

Figure S82. IR spectrum of **P4** in KBr pellets.

High resolution mass spectrometry (HRMS) chart

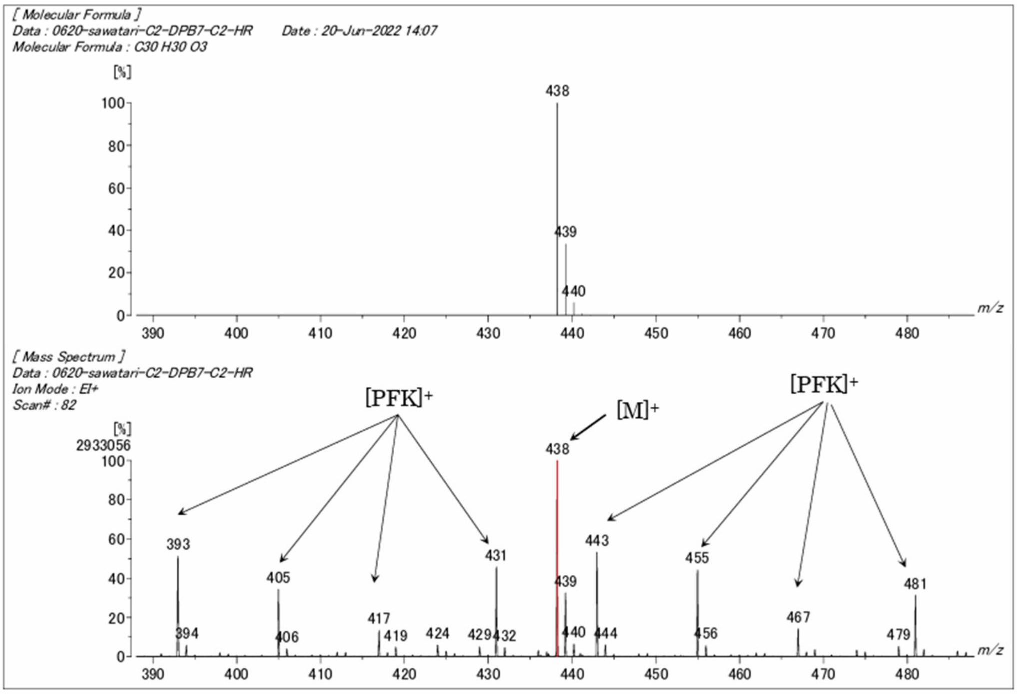

Figure S83. HRMS spectra of **M1** (above: calculated data; below: experimental data).

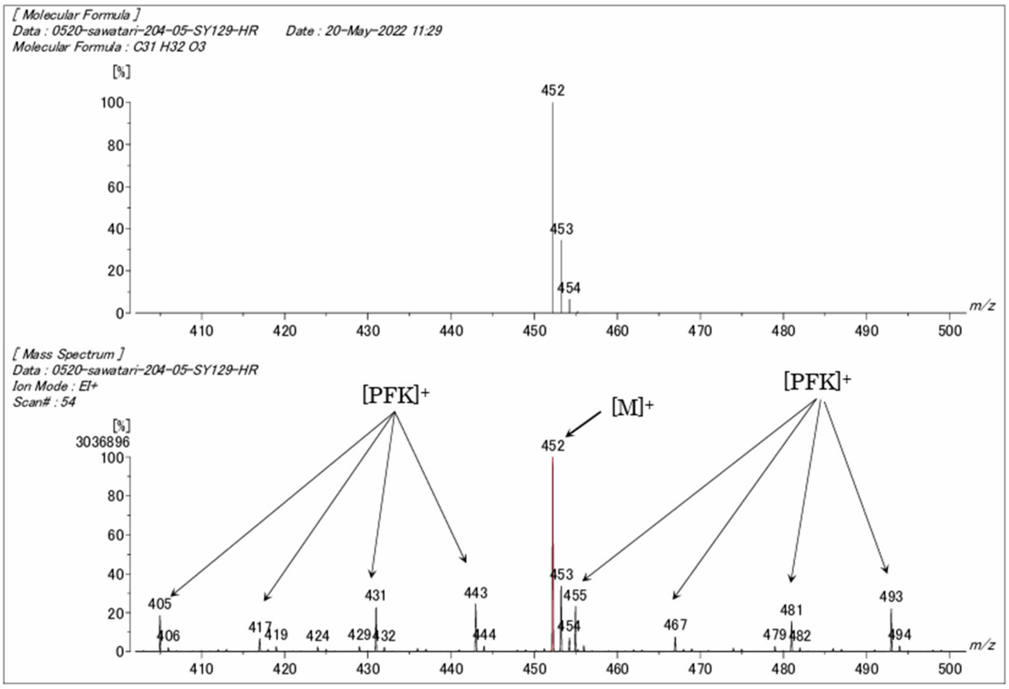

Figure S84. HRMS spectra of **M2** (above: calculated data; below: experimental data).

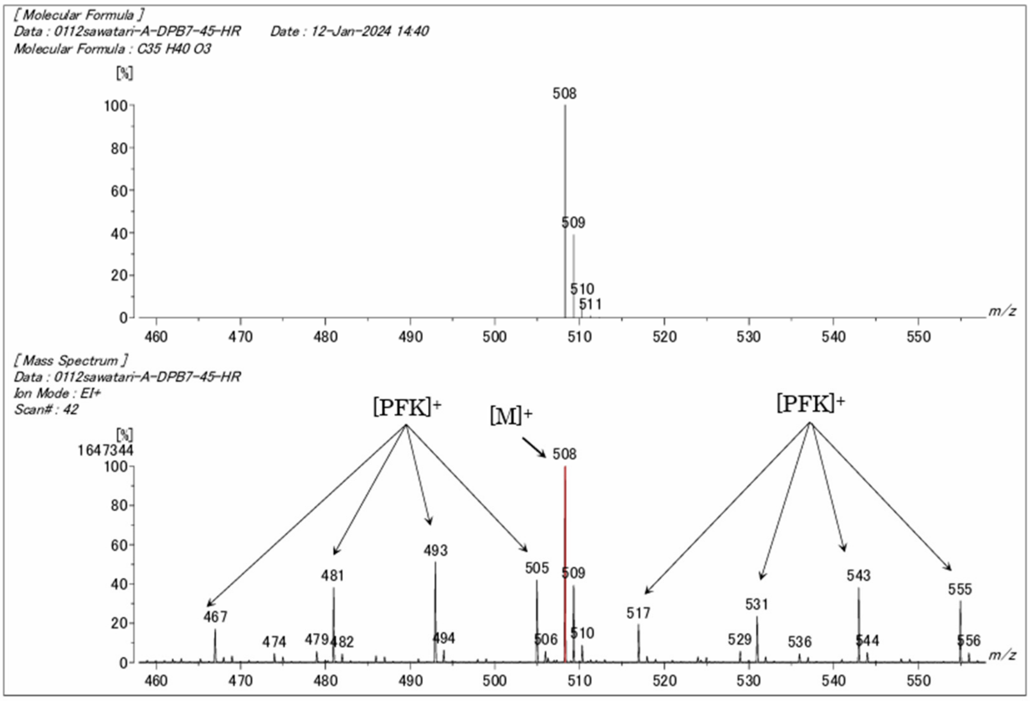

Figure S85. HRMS spectra of **M3** (above: calculated data; below: experimental data).

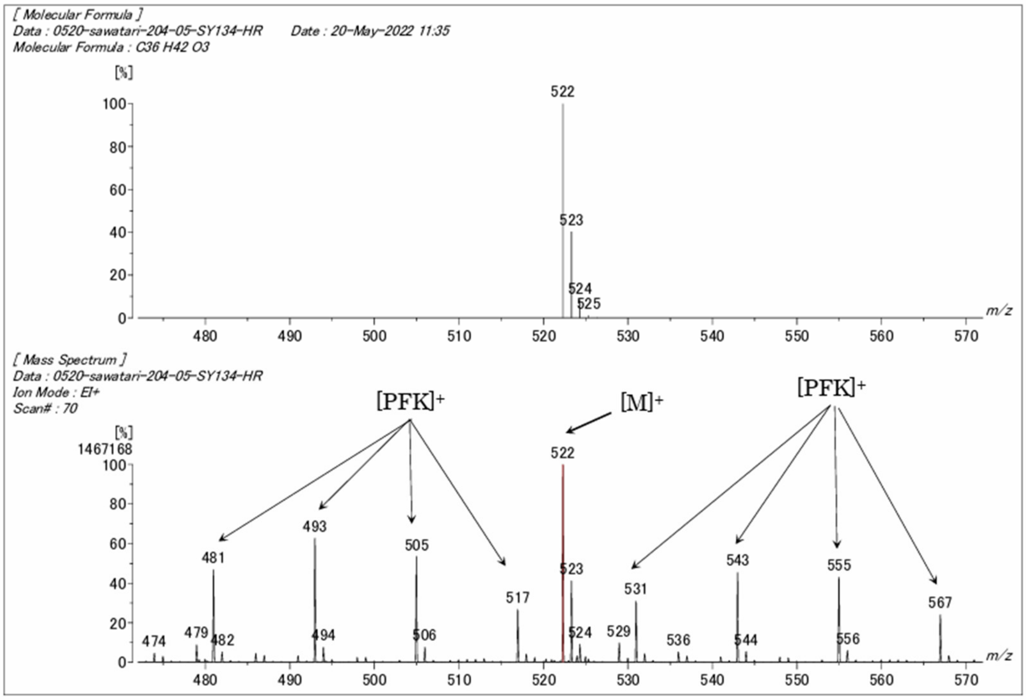

Figure S86. HRMS spectra of **M4** (above: calculated data; below: experimental data).

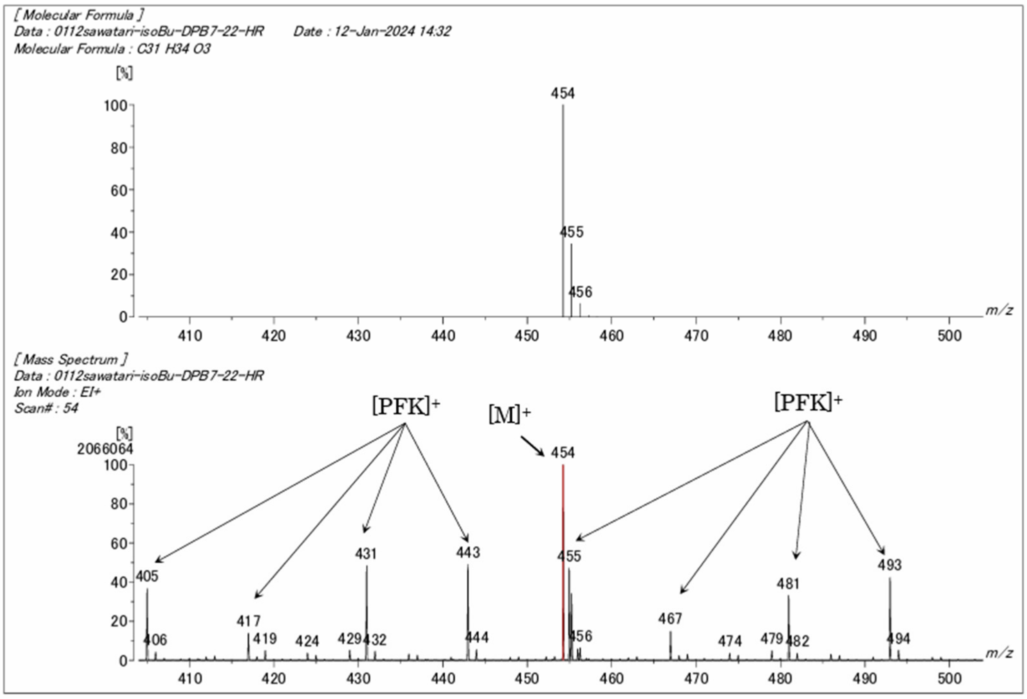

Figure S87. HRMS spectra of **M5** (above: calculated data; below: experimental data).

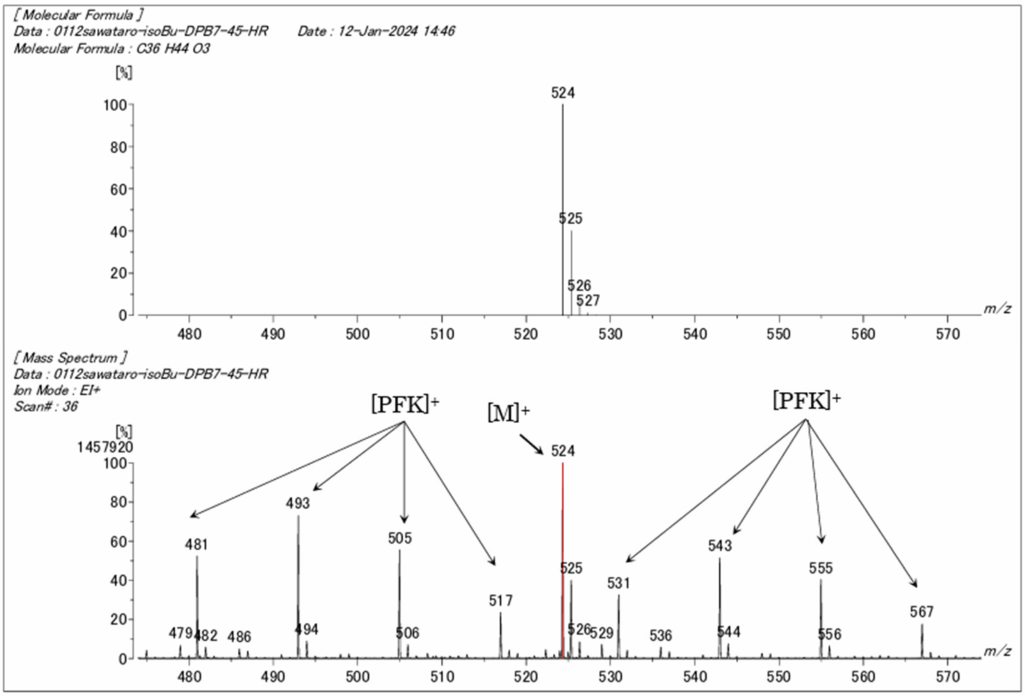

Figure S88. HRMS spectra of **M6** (above: calculated data; below: experimental data).
